# Supplementary material for: A chromosome-scale assembly reveals chromosomal aberrations and exchanges generating genetic diversity in Coffea arabica germplasm
Source: Nat Commun. 2024 Jan 23;15:463. doi: 10.1038/s41467-023-44449-8 (PMC10805892; doi:10.1038/s41467-023-44449-8)
Supplement: Supplementary file 1 — Supplementary Information [file 41467_2023_44449_MOESM1_ESM.pdf]

A chromosome-scale assembly reveals chromosomal aberrations and exchanges generating genetic diversity in *Coffea arabica* germplasm

Scalabrin *et al.*

Supplementary Method 1. Manual curation of the assembly

The scaffolds that were automatically obtained using SALSA[1] were merged or broken according to the procedure described below, which was repeated for 20 cycles, until the convergence of the Hi-C signal was achieved:

a) Scaffolds were aligned towards the Caturra reference genome (GenBank Assembly Accession GCA_003713225.1) with nucmer and the parameter --mum. Alignments were filtered with delta-filter with minimum identity 90% and minimum length 200 Kb (-i 90 -l 200000). Filtered alignments were plotted with mummerplot. Nucmer, delta-filter, and mummerplot are part of the MUMmer package[2].

b) Hi-C reads were aligned with the current version of the genome and visualized with Juicer[3].

c) Visual comparison of the plots against the Caturra reference genome and Hi-C signal was used to identify breakpoints and scaffold junctions. Breakpoints and scaffolds junctions were validated by aligning ONT reads using the NGMLR software[4] with the following parameters "-x ont -i 0.8 -R 0.5 --mismatch -2" and visualized using Integrative Genomics Viewer[5], generating superscaffolds.

Once the final chromosome pseudomolecules were defined, the unplaced scaffolds were aligned against the final chromosome pseudomolecules using the MUMmer package as described above. Each unplaced scaffold that aligned for more than 60% of its length against the homoeologous region in the chromosome pseudomolecules of both homoeologs was removed from the final assembly.

Supplementary Method 2. Dating LTR-retrotransposon insertions

For this analysis, we considered intact LTR-retrotransposons identified by EDTA[6]. LTR sequences were aligned using the EMBOSS Stretcher software[7] with default parameters using the -filter option. The age of retrotransposon insertion was estimated based on LTR divergence using the Kimura Two-Parameter distance method with a substitution rate of 1.3×10^{-8} substitutions per site per year used for annuals[8] and divided by 3 years to account for an approximate generation time of 3 years (duration of the juvenile phase).

Supplementary Method 3. Analysis and phylogeny of the 2,683-bp CRM-derived monomer that has generated the tandem repeated array in Chr7c and Chr7e illustrated in Fig. 2b and in Supplementary Figs. 36-37

Monomeric sequences were extracted with StringDecomposer[9] using one representative monomer from the array in each subgenome as an input. Matches were retained if they showed sequence identity higher than 80 % with the input. Multiple alignment was generated using MAFFT[10]. The phylogenetic tree shown in Supplementary Fig. 36 was constructed using IQ-

TREE[11] and plotted with iTOL[12]. Outliers arising from misalignments and showing a distance higher than 0.1 were removed from the phylogenetic tree.

Supplementary Method 4. Identification of introgressed chromosome segments in individual accessions and haplotype frequency analysis in three groups of introgression lines

In each accession, blocks of consecutive genomic windows showing ≥ 100 homozygous SNPs when compared to the Bourbon reference were considered to carry two copies of non-*C. arabica* haplotypes. Blocks of consecutive genomic windows showing < 100 homozygous SNPs and ≥ 100 heterozygous SNPs with respect to the Bourbon reference were considered to carry one copy of non-*C. arabica* haplotypes. Individual windows that did not surpass those thresholds within a block of windows that surpassed those thresholds were reconsidered lowering both thresholds to 50 SNPs. For the last window that is located at the end of each chromosomal pseudomolecule, which may contain less than 100 Kb of non-repetitive DNA, both thresholds were reduced proportionally to the window size (Source Data and Supplementary Figs. 46-50).

The windows that remained below those thresholds were considered to carry two copies of *C. arabica* haplotypes. A total of 44 accessions in the WGS panel that showed at least one non-*C. arabica* chromosome segment were considered to be introgression lines.

Introgressed haplotype frequency was calculated on a per-window basis separately in three groups of introgression lines (see above and Supplementary Data 1 for further details):

1) 37 introgression lines that could be expected to carry canephora introgression deriving from the Timor hybrid, hereafter also referred to as Híbrido de Timor (HDT)
2) 1 introgression line that could be expected to carry liberica introgression (S288)
3) 6 specimens that were unexpected to carry *Coffea* sp. introgression, namely CHF1, GNG1, GUG3, Kent, SL28 and SL34.

Supplementary Method 5. Analysis of genetic diversity in an extended germplasm panel based on GBS-data and comparison with the WGS panel

We used an extended diversity panel[13] that included cultivated and spontaneous germplasm collectively referred to as Arabica-like based on an *a priori* assignment to the *C. arabica* species and to lineages deriving from its interspecific hybrids, as well as accessions of present-day populations of the diploid progenitor species mainly held at the *ex situ* germplasm repository of CATIE. This analysis was aimed at validating the relevance, conformity and representativeness of the germplasm sample included in the WGS panel and at extending the observations and findings that we obtained using the WGS panel to the widest available intraspecific diversity in *C. arabica*.

As described in our previous work[13], the extended diversity panel contained Arabica-like accessions that were sorted *a priori* in 6 groups: Bourbon/Typica, Landrace cultivated, Survey Ethiopia, Survey Yemen, Canephora introgressed and Liberica introgressed (Supplementary

Data 3). Based on this *a priori* classification, the Bourbon/Typica group was expected to include Yemen-derived cultivars and mutants of pure *C. arabica* origin. The Landrace cultivated group was expected to consist of cultivated germplasm, including African heirloom varieties as well as unknown introductions from Africa and India. As for the African landraces, the detail of the country of sampling was known for most of the accessions. This set included landraces from Ethiopia, Eritrea and Sudan—the area that is thought to overlap with the center of origin of the species. Other landraces were sampled in Congo, Kenya, Madagascar, Reunion (also referred to as Bourbon Island), Malawi and Tanzania. The Survey Ethiopia group included Ethiopian ecotypes maintained at CATIE and originating from FAO prospections in 1964-1965[14], from prospections conducted by the French Office de la recherche scientifique et technique outre-mer (ORSTOM) in 1966[15,16] and from the 'Lejeune survey' or other surveys conducted in Ethiopia in the 1960's. The Survey Yemen group included Yemeni ecotypes maintained at CATIE and originating from FAO prospections in 1964-1965 as described above as well as from a recent prospection conducted by the University of Sana'a.

We called 8,169 SNPs in the entire set of 834 accessions that had less than 20 % of missing genotypic data (Supplementary Data 3). After removing accessions of the diploid progenitor species, we called 1,992 SNPs in the set of 771 Arabica-like accessions. Subsequent to the identification and removal of accessions carrying *Coffea* sp. introgression (see below for the procedure of identification of known and criptic introgression), we genotyped 1,397 SNPs in a panel of 734 *bona fide* accessions of *C. arabica*.

Supplementary Method 6. Identification of criptic *Coffea* sp. introgression in an extended germplasm sample based on GBS-data

No matter their *a priori* classification, accessions of the extended diversity panel were treated based on their location on the PCA bidimensional space, compared to their diploid progenitor species. The first two components of the PCA explained 20.8 % of the variance (Supplementary Fig. 52a**)**. PC1 explained variance originating from *C. canephora* diversity. PC2 explained variance originating from *C. eugenioides* diversity. A total of 771 accessions formed a group of Arabica-like germplasm that clearly separated from present-day populations of their diploid progenitor species (Supplementary Fig. 52a). Arabica-like germplasm included accessions belonging to all groups: Bourbon/Typica, Landrace cultivated, Survey Ethiopia, Survey Yemen, Canephora introgressed and Liberica introgressed. At a closer inspection of the section of the PCA space populated by the Arabica-like germplasm (Supplementary Fig. 52b), the accessions expected to carry canephora or liberica introgression tended to separate from the area occupied by the rest of Arabica-like germplasm. Canephora introgression lines were shifted towards the PCA space occupied by accessions of diploid *C. canephora*. The rest of Arabica-like germplasm showed a continuous dispersion following approximately the diagonal between the PC1 and PC2 axes. When PCA was re-run using the subset of Arabica-like germplasm (excluding accessions of the progenitor species) most of the expected introgression lines separated from the rest of the Arabica-like germplasm (Supplementary Fig. 52c). Expected canephora introgression lines were separated by PC1. Expected liberica introgression lines were separated by PC2. Expected

canephora introgression lines were represented by several accessions that were introduced at CATIE from Colombia as well as by other known Timor hybrid derivatives including two independent entries of ‘Marsellesa’. Expected liberica introgression lines were represented by S288 and S795 as well as by several accessions that were introduced at CATIE from India and were coded with the acromym BA, which stands for the Central Coffee Research Institute (CCRI) in Balehonnur (BA), India. CCRI initiated a systematic collection of coffee genetic resources from coffee plantations in the Balehonnur area in 1925. From this material, breeders at CCRI obtained the selections S288 and S795.

Other accessions that were classified *a priori* as either Bourbon/Typica or landrace cultivated appeared to separate from the rest of the Arabica-like germplasm in a similar way as did introgression lines and were therefore reclassified on the basis of this information (Supplementary Fig. 52e). Some accessions that were expected to carry canephora or liberica introgression were located in close contiguity with the area occupied by the rest of the Arabica-like germplasm (Supplementary Fig. 52d) and other accessions that were expected to carry canephora or liberica introgression overlapped with the area occupied by the rest of the Arabica-like germplasm (Supplementary Fig. 52f). We reasoned that these introgression lines may contain a small residual part of introgressed chromosome segments that are not detected by PCA. The original classification of these introgression lines was maintained with the appended note that introgression, if any, could not be inferred from PCA (Supplementary Fig. 52f).

In order to support the evidence obtained from the PCA and to confirm the presence of introgressed chromosome segments in the expected introgression lines as well as of criptic introgression in Arabica-like germplasm, we considered 5 types of variant sites with respect to the Bourbon reference in each accession:

1) variant sites that were polymorphic in the population of *C. canephora* and were shared with one or more expected canephora introgression lines of Timor hybrid derivatives (Type-1 SNPs)
2) variant sites that were polymorphic in the population of *C. canephora* but were not shared with any expected introgression line of Timor hybrid derivatives (Type-2 SNPs)
3) variant sites that were shared with one or more expected introgression lines of Timor hybrid derivatives but were identical to the Bourbon reference in the population of *C. canephora* (Type-3 SNPs)
4) variant sites that were not shared with the population of *C. canephora* and with expected canephora introgression lines of Timor hybrid derivatives (Type-4 SNPs)
5) variant sites that were shared with one or more expected liberica introgression lines (Type-5 SNPs)

The chromosome plots showing the genomic distribution of Type-1 and Type-3 sites[17] indicated that 17 out of the 20 expected canephora introgression lines of Timor hybrid derivatives carried introgressed chromosome segments that largely overlapped with those found in expected and unexpected canephora introgression lines of the WGS panel (Supplementary Data 3). The remaining 3 expected canephora introgression lines of Timor hybrid derivatives did not carry any

detectable introgression. They included a Marsellesa specimen held at CIRAD and annotated as abnormal phenotype as well as an undetailed Catimor specimen and the accession IPR103 held at CATIE (Supplementary Data 3). We noted that dRAD sequencing captures a non-randomly distributed portion of the genome (see Supplementary Fig. 54e for the distribution of the 5 types of variant sites in one representative accession of *C. canephora*, in which Type-2 variant sites were expected to be scattered evenly and genome-wide). Therefore, we could not determine whether advanced backcross generations in the GBS panel either carry only small introgressed chromosome segments or carry residual introgression only in pericentromeric regions that were not captured by dRAD sequencing or they have reverted to a full *C. arabica* genetic background following the complete purging of introgressed haplotypes.

In a similar way, the chromosome plots showing the genomic distribution of Type-4 and Type-5 sites indicated that 8 (i.e. S-288, S-795, BA-02, BA-03, BA-08, BA-16, BA-21, BA-35) out of the 14 expected liberica introgression lines carried signatures of introgressed chromosome segments[17] (Supplementary Data 3). The remaining 6 expected liberica introgression lines of Timor hybrid derivatives (S-333, BA-10, BA-13, BA-27 (T.02692), BA-27 (T.02760), BA-36), including two independent entries of the accession BA-27, did not carry any detectable introgression[17], which may represent more advanced stages of backcrossing to *C. arabica*.

Supplementary Method 7. Detection of homoeologous copy number variation

We used the χ-scan software for identifying homoeologous copy number variations that may arise from exchanges between hemoeologous chromosomes or chromosomal aberrations of different types such as aneuploidies, deletions and duplications[18]. χ-scan was originally developed for identifying somatic homologous copy number variation among clonal individuals of the same heterozygous genotype based on the identification of what we termed Reduction Of Heterozygosity (ROH). This approach was renamed here Reduction Of Homoeologous Heterozygosity (ROH_H).

Read alignments and SNP calling were performed with the same software and parameters as described in the main text for the detection of homologous SNPs with the exception that DNA reads were aligned to each subgenome of the reference, separately, including chromosome pseudomolecules and unanchored scaffolds. The resulting catalogue contained raw homoeologous SNPs in vcf file format. Raw homoeologous SNPs were filtered using the same procedure that we used for filtering raw homologous SNPs, as described in the main text. Deviations from the variant frequency that is expected under the normal condition of CAN:EUG=2:2 homoeologous copy number (ROH_H) were identified using the chi_reads algorithm of the χ-scan software as described in[18], using sliding windows of variable size containing 500 high quality SNPs with an overlap of 250 SNPs between windows. Depth of Coverage (DOC) was calculated using the command *genomecov* in *bedtools* in 4,467 non-overlapping genomic windows of variable size, containing 100 Kb of non-repetitive DNA. The average DOC value for each window was calculated considering only the coverage of sites in non-repetitive DNA regions within each window. For each accession, average DOC in each

window was normalized to genome-wide average coverage in order to account for among-accessions variation in depth of sequencing and then expressed relative to the same value that was obtained in ‘Bourbon’. For homoeologous exchanges, we retained cases where a significant allelic imbalance based on the χ-scan statistical test was detected on the alignments against both homoeologous chromosomes across a region of at least 200 Kb and a difference in homoeologous SNPs average variant frequency greater than 0.2 was detected. When differences between 0.2 and 0.4 were detected, we assumed the presence of a chimeric event resulting either from somatic mosaicism or from genotype mixtures; when differences between 0.4 and 0.6 were detected we assumed the presence of a heterozygous event; when differences greater than 0.8 were detected we assumed the presence of homozygous events. For all these cases, we also required the presence of normalized depth of coverage ratios that fit the respective expectations (between 1 and 1.5 and between 0.5 and 1 for chimeric events, between 1.5 and 0.5 for heterozygous events, between 2 and 0 for homozygous ones). Aneuploidies were inferred when at least 95 % of windows along a chromosome resulted statistically significant based on χ-scan statistical test with a concordant variant frequency change and reciprocal results were observed on the two subgenomes. Trisomies were identified when the coverage ratio for one of the subgenomes was 1.5 and the homoeologous SNP variant frequencies for the corresponding subgenome were 0.6. For the other subgenome we required a coverage ratio of 1 and an average homoeologous SNP variant frequencies of 0.4. The expectations for monosomies were of a coverage ratio for one of the subgenomes of 0.5 and of homoeologous SNP variant frequencies for the corresponding subgenome 0.33. For the other subgenome, we required a coverage ratio of 1 and an average homoeologous SNP variant frequencies of 0.66. When the significance criterion of at least 95 % of windows along a chromosome was statistically significant according to the χ-scan statistical test with a concordant variant frequency change and reciprocal results were observed on the two subgenomes, we assumed the presence of a genetic chimerism due to either somatic mosaicism or genotype mixtures, regardless of the expected thresholds for coverage ratio and homoeologous variant frequencies were met. We also detected large deletions or duplications using the same criteria that we described for aneuploidies, and requiring events to be larger than 1 Mbp but not encompassing a whole chromosome.

In order to exclude that low genome-wide coverage could affect specificity and sensitivity of this analysis and to ensure that ROH_H and DOC thresholds based on theoretical expectations are valid to sort homoeologous unbalances into discrete categories across a wide range of among-samples variation in read coverage, we performed a subsampling experiment using 2 out of the 4 *C. arabica* accessions that were resequenced at high coverage in this study (Supplementary Table 10).

Libraries were prepared using the Celero™ DNA-Seq kit (Tecan, Männedorf, Switzerland) following the manufacturer’s instructions. Libraries were quantified using a Qubit 2.0 Fluorometer (Invitrogen, Carlsbad, CA, USA) and quality checked using an Agilent 2100 Bioanalyzer High Sensitivity DNA assay (Agilent technologies, Santa Clara, CA, USA). Libraries were sequenced on a NovaSeq 6000 (Illumina, San Diego, CA, USA) in a paired-end 150 bp mode. WGS reads were aligned with each subgenome of the reference, separately, including chromosome pseudomolecules and unanchored scaffolds.

First, detection of homoeologous copy number variation was performed in the 4 accessions using full coverage with the analytical pipeline and the ROH_H and DOC thresholds as described above. Once a homoeologous exchange was identified in the accession ET47, the analysis was repeated by simulating low genome-wide coverages. We extracted from the .bam file random samples of aligned reads that simulated average coverages of 4X, 6X, 8X and 10X and compared the χ-scan output with that obtained using the whole sequencing yield that corresponded to a coverage of 44X aligned reads[17]. Even at as low a coverage as 4X we could identify the same events (a CAN:EUG=4:0 homoeologous copy number variation at the bottom of Chr7 and a CAN:EUG=1:3 homoeologous copy number variation at the bottom of Chr10) that were found using the whole coverage of 44X aligned reads (Supplementary Fig. 60) without detecting any additional event. Then, we selected one of the 3 accessions that did not show homoeologous copy number variation using their full coverage (Kenya-SL28). We extracted from the .bam file random samples of aligned reads that simulated average coverages of 2X, 4X, 6X and 8X and compared the χ-scan output with that obtained using the whole sequencing yield that corresponded to a coverage of 34X aligned reads[17]. Even at as low a coverage as 2X the specimen Kenya-SL28 did not show homoeologous copy number variation, confirming that specificity of the assay does not decrease at low genome-wide coverages.

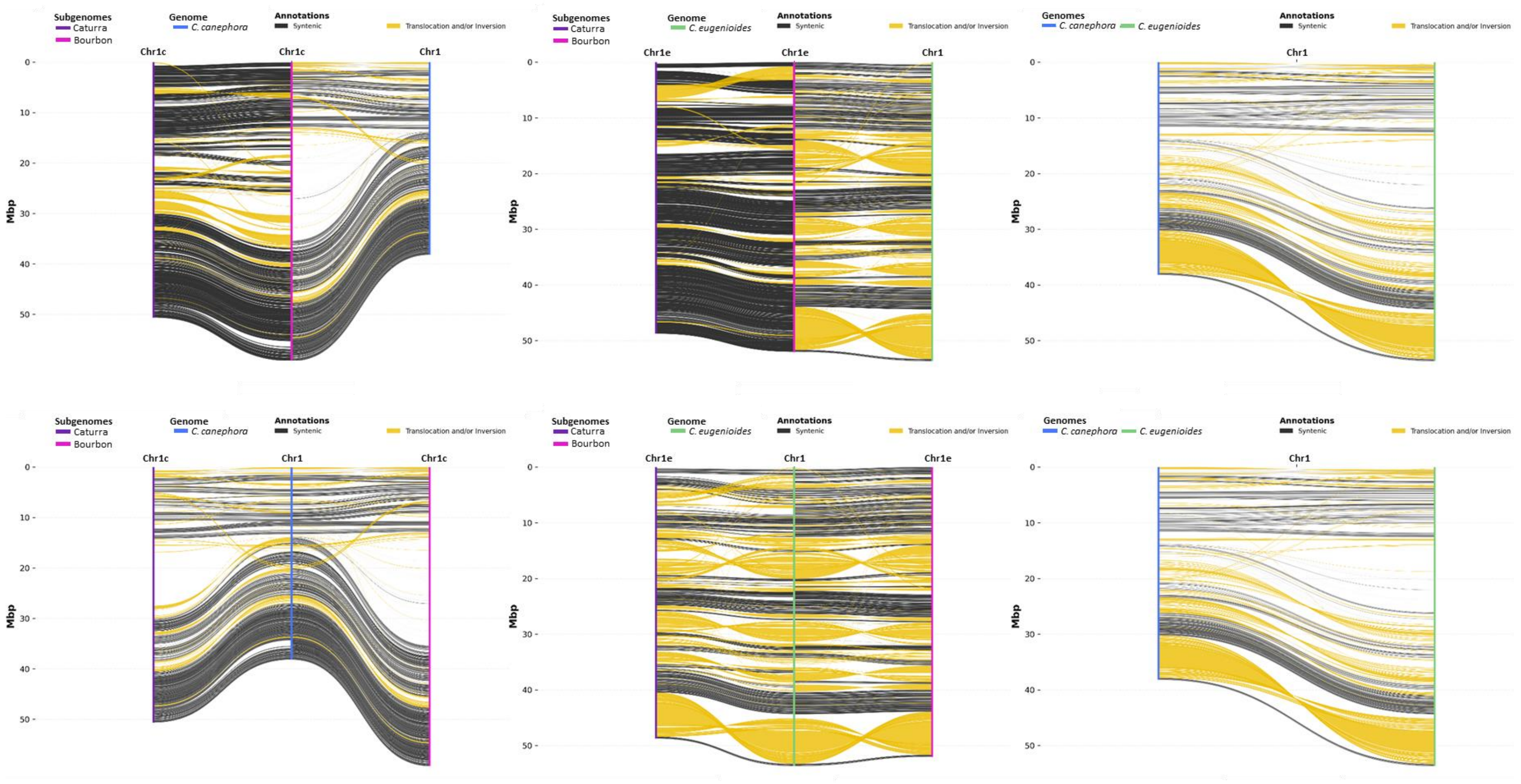

Supplementary Fig. 1. Collinearity among homologous and homoeologous chromosomes 1 (Chr1) among *Coffea* genome assemblies. In the upper panels, the ‘Bourbon’ assembly of this paper (middle) is compared with the assembly of *C. arabica* ‘Caturra’ (left, GenBank Assembly Accession GCA_003713225.1) and the assemblies of a present-day representative of the diploid progenitors species (right, GenBank Assembly Accession GCA_900059795.1 for *C. canephora* and GenBank Assembly Accession GCA_003713205.1 for *C. eugenioides*). The pairwise comparison between the homoeologous chromosomes in the diploid species is shown in the ideograms to the right. In the lower panels, the assemblies of a present-day representative of the diploid progenitors species (middle, GenBank Assembly Accession GCA_900059795.1 for *C. canephora* and GenBank Assembly Accession GCA_003713205.1 for *C. eugenioides*) are compared to the ‘Bourbon’ assembly of this paper (right) and the assembly of *C. arabica* ‘Caturra’ (left, GenBank Assembly Accession GCA_003713225.1). The pairwise comparison between the homoeologous chromosomes in the diploid species is shown in the ideograms to the right. Y-axes indicate million base pairs (Mbp).

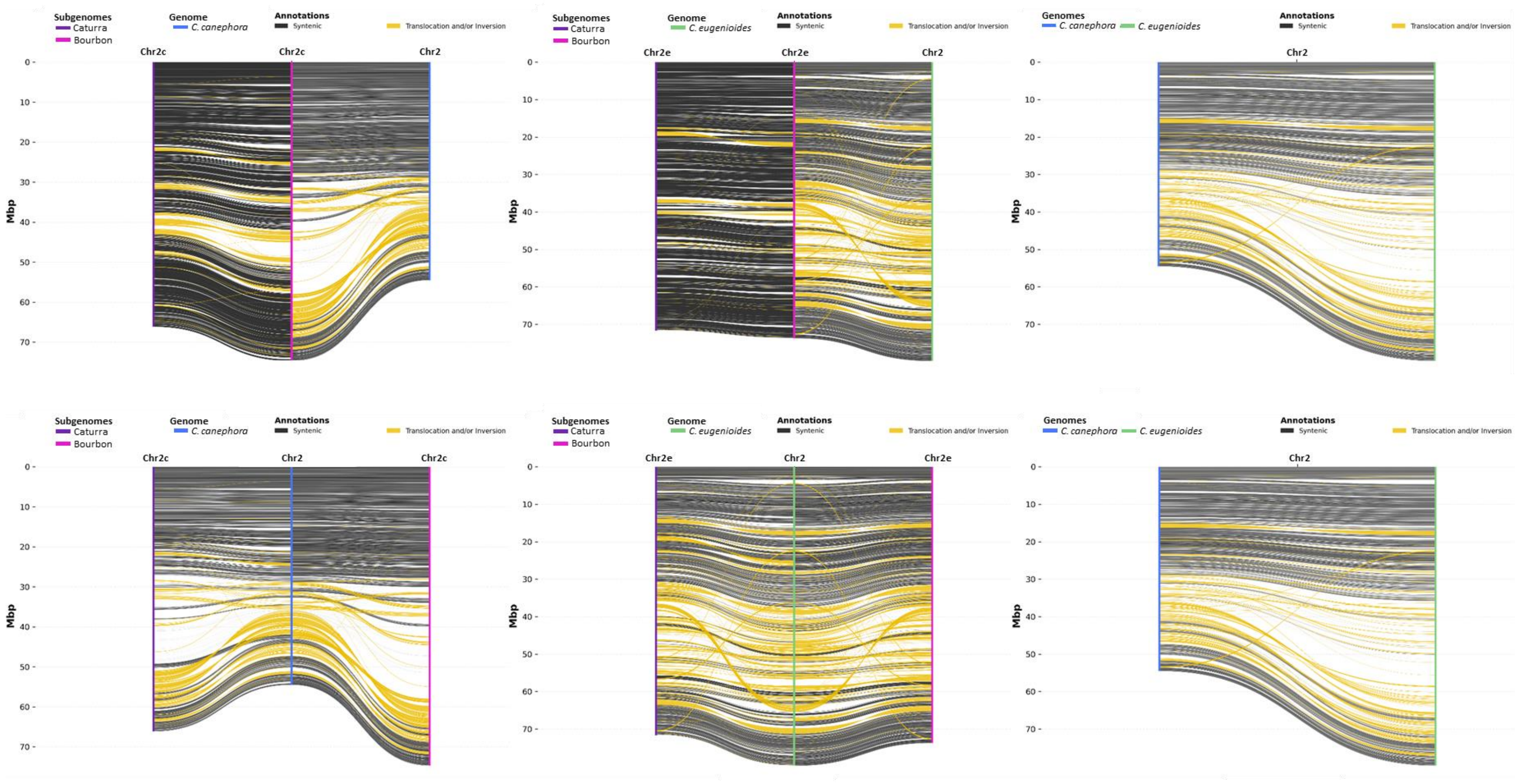

Supplementary Fig. 2. Collinearity among homologous and homoeologous chromosomes 2 (Chr2) among *Coffea* genome assemblies. In the upper panels, the ‘Bourbon’ assembly of this paper (middle) is aligned with the assembly of *C. arabica* ‘Caturra’ (left, GenBank Assembly Accession GCA_003713225.1) and the assemblies of a present-day representative of the diploid progenitors species (right, GenBank Assembly Accession GCA_900059795.1 for *C. canephora* and GenBank Assembly Accession GCA_003713205.1 for *C. eugenioides*). The pairwise comparison between the homoeologous chromosomes in the diploid species is shown in the ideograms to the right. In the lower panels, the assemblies of a present-day representative of the diploid progenitors species (middle, GenBank Assembly Accession GCA_900059795.1 for *C. canephora* and GenBank Assembly Accession GCA_003713205.1 for *C. eugenioides*) are compared to the ‘Bourbon’ assembly of this paper (right) and the assembly of *C. arabica* ‘Caturra’ (left, GenBank Assembly Accession GCA_003713225.1). The pairwise comparison between the homoeologous chromosomes in the diploid species is shown in the ideograms to the right. Y-axes indicate million base pairs (Mbp).

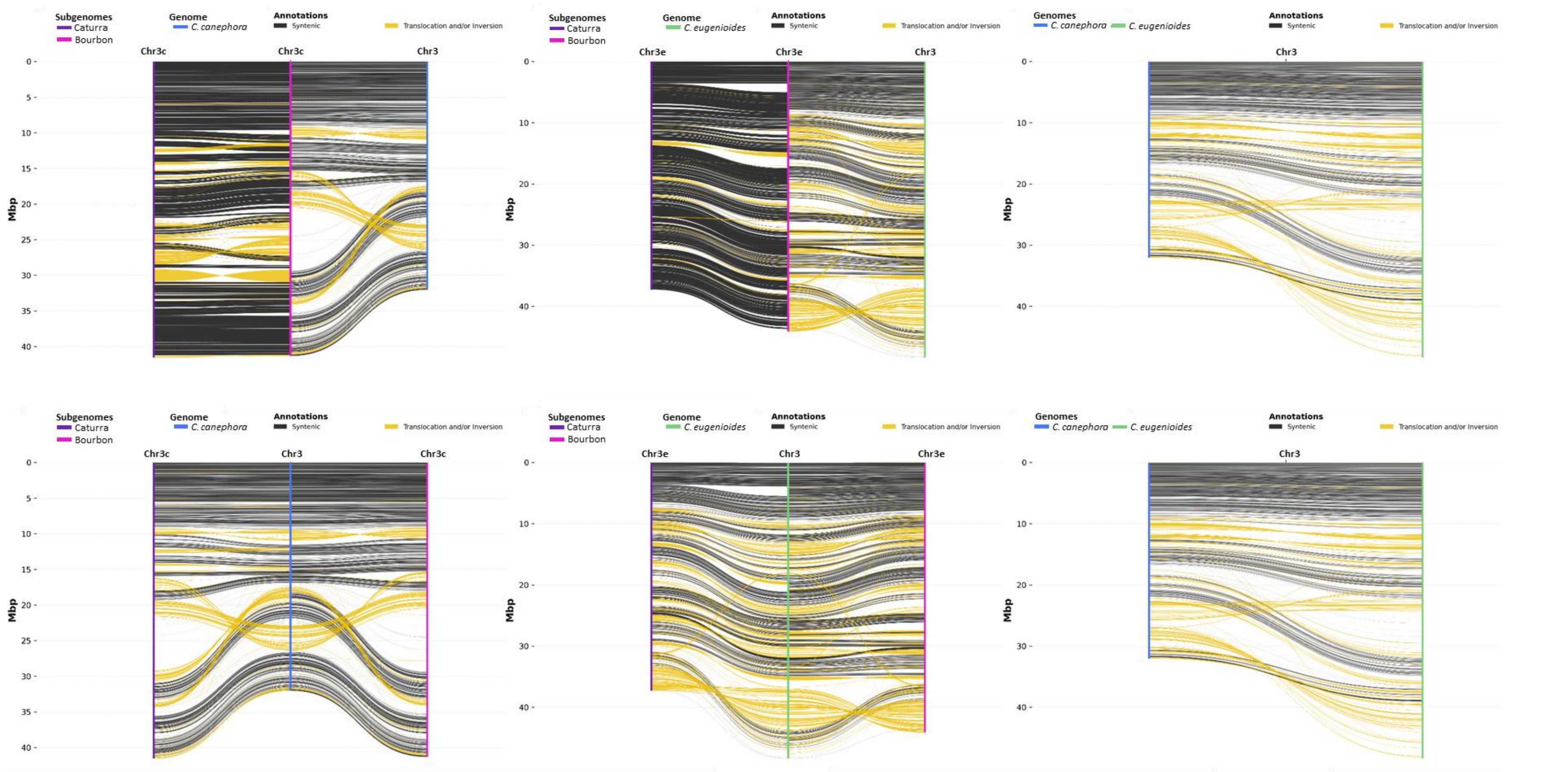

Supplementary Fig. 3. Collinearity among homologous and homoeologous chromosomes 3 (Chr3) among *Coffea* genome assemblies. In the upper panels, the ‘Bourbon’ assembly of this paper (middle) is aligned with the assembly of *C. arabica* ‘Caturra’ (left, GenBank Assembly Accession GCA_003713225.1) and the assemblies of a present-day representative of the diploid progenitors species (right, GenBank Assembly Accession GCA_900059795.1 for *C. canephora* and GenBank Assembly Accession GCA_003713205.1 for *C. eugenioides*). The pairwise comparison between the homoeologous chromosomes in the diploid species is shown in the ideograms to the right. In the lower panels, the assemblies of a present-day representative of the diploid progenitors species (middle, GenBank Assembly Accession GCA_900059795.1 for *C. canephora* and GenBank Assembly Accession GCA_003713205.1 for *C. eugenioides*) are compared to the ‘Bourbon’ assembly of this paper (right) and the assembly of *C. arabica* ‘Caturra’ (left, GenBank Assembly Accession GCA_003713225.1). The pairwise comparison between the homoeologous chromosomes in the diploid species is shown in the ideograms to the right. Y-axes indicate million base pairs (Mbp).

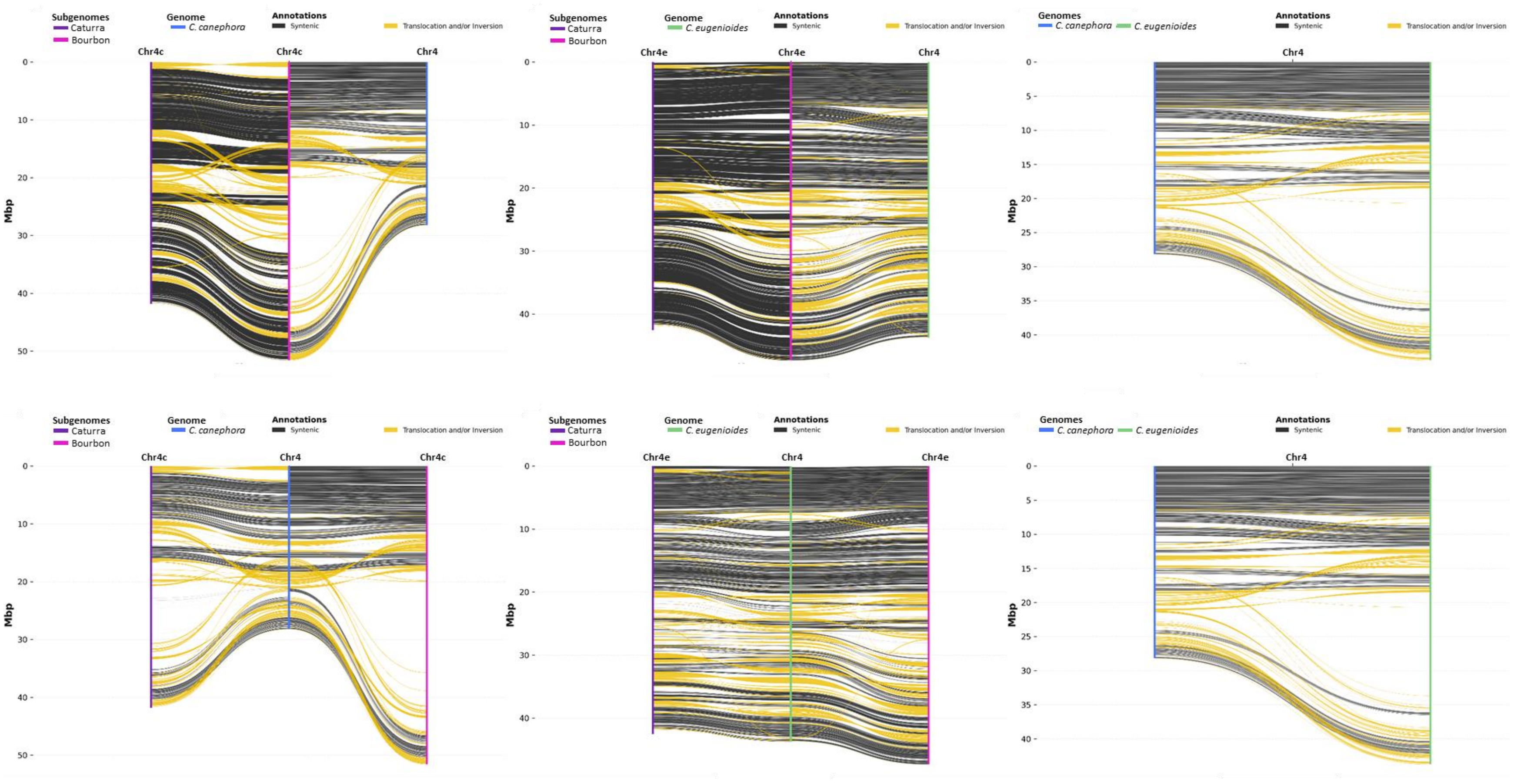

Supplementary Fig. 4. Collinearity among homologous and homoeologous chromosomes 4 (Chr4) among *Coffea* genome assemblies. In the upper panels, the 'Bourbon' assembly of this paper (middle) is aligned with the assembly of *C. arabica* 'Caturra' (left, GenBank Assembly Accession GCA_003713225.1) and the assemblies of a present-day representative of the diploid progenitors species (right, GenBank Assembly Accession GCA_900059795.1 for *C. canephora* and GenBank Assembly Accession GCA_003713205.1 for *C. eugenioides*). The pairwise comparison between the homoeologous chromosomes in the diploid species is shown in the ideograms to the right. In the lower panels, the assemblies of a present-day representative of the diploid progenitors species (middle, GenBank Assembly Accession GCA_900059795.1 for *C. canephora* and GenBank Assembly Accession GCA_003713205.1 for *C. eugenioides*) are compared to the 'Bourbon' assembly of this paper (right) and the assembly of *C. arabica* 'Caturra' (left, GenBank Assembly Accession GCA_003713225.1). The pairwise comparison between the homoeologous chromosomes in the diploid species is shown in the ideograms to the right. Y-axes indicate million base pairs (Mbp).

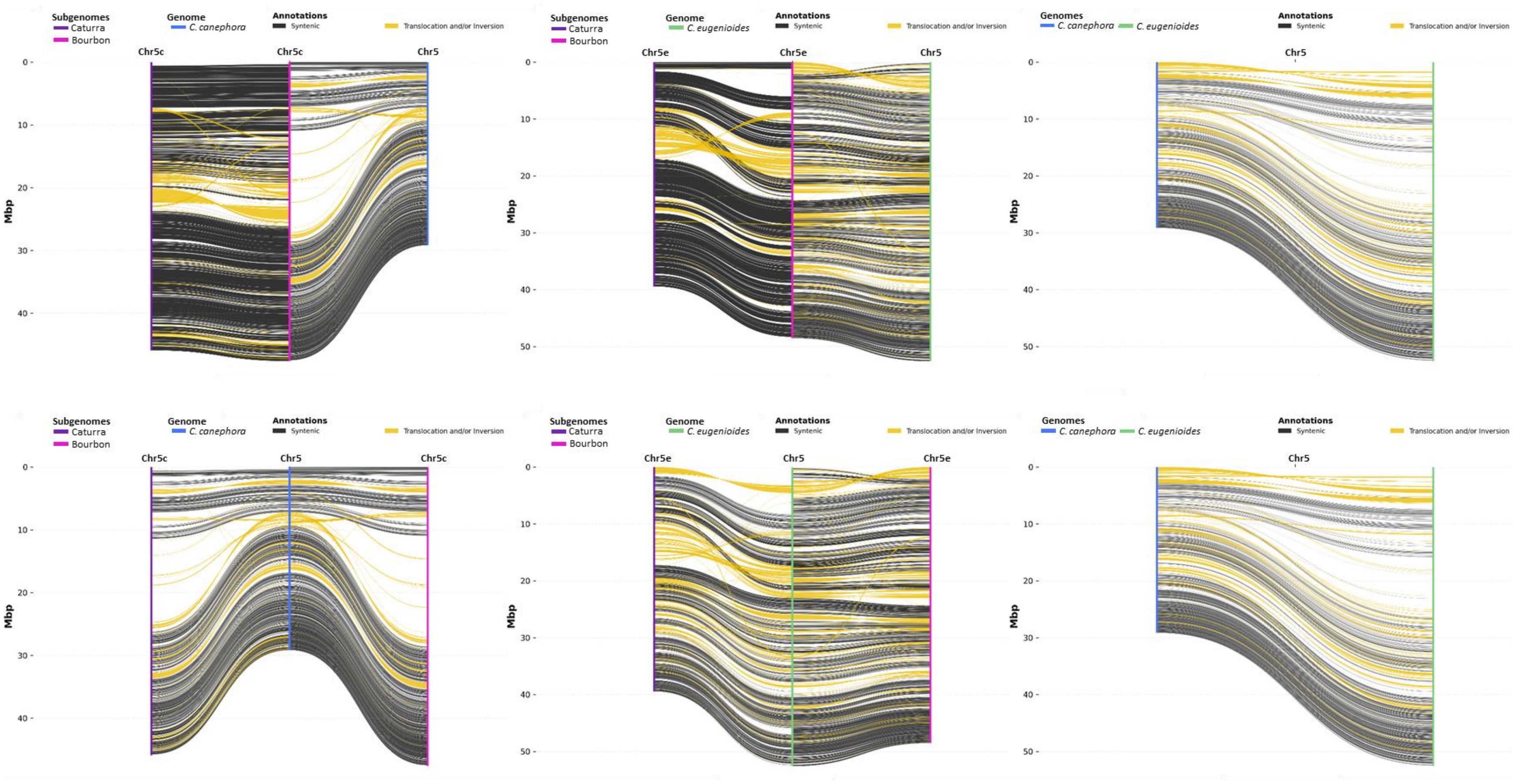

Supplementary Fig. 5. Collinearity among homologous and homoeologous chromosomes 5 (Chr5) among *Coffea* genome assemblies. In the upper panels, the 'Bourbon' assembly of this paper (middle) is aligned with the assembly of *C. arabica* 'Caturra' (left, GenBank Assembly Accession GCA_003713225.1) and the assemblies of a present-day representative of the diploid progenitors species (right, GenBank Assembly Accession GCA_900059795.1 for *C. canephora* and GenBank Assembly Accession GCA_003713205.1 for *C. eugenioides*). The pairwise comparison between the homoeologous chromosomes in the diploid species is shown in the ideograms to the right. In the lower panels, the assemblies of a present-day representative of the diploid progenitors species (middle, GenBank Assembly Accession GCA_900059795.1 for *C. canephora* and GenBank Assembly Accession GCA_003713205.1 for *C. eugenioides*) are compared to the 'Bourbon' assembly of this paper (right) and the assembly of *C. arabica* 'Caturra' (left, GenBank Assembly Accession GCA_003713225.1). The pairwise comparison between the homoeologous chromosomes in the diploid species is shown in the ideograms to the right. Y-axes indicate million base pairs (Mbp).

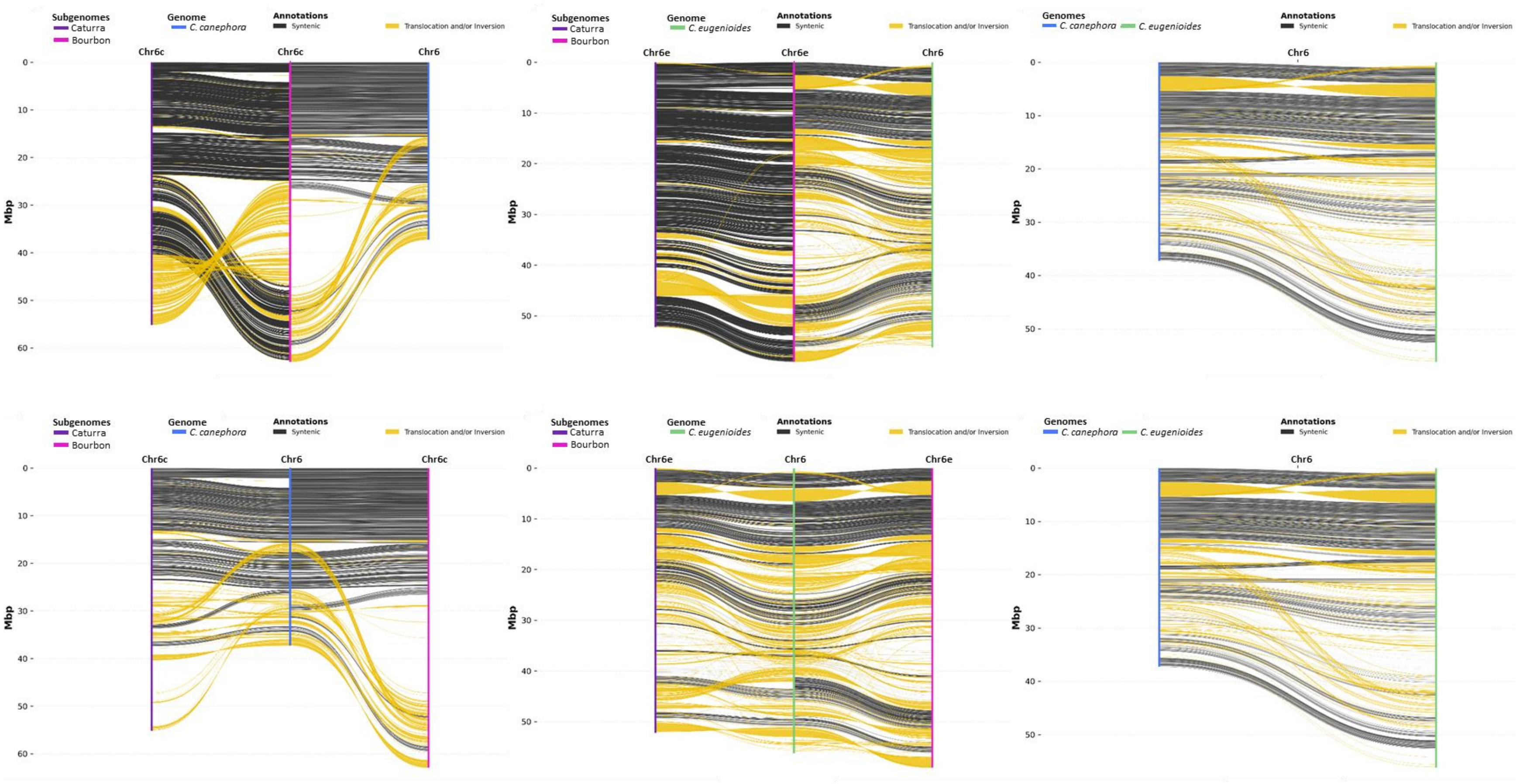

Supplementary Fig. 6. Collinearity among homologous and homoeologous chromosomes 6 (Chr6) among *Coffea* genome assemblies. In the upper panels, the ‘Bourbon’ assembly of this paper (middle) is aligned with the assembly of *C. arabica* ‘Caturra’ (left, GenBank Assembly Accession GCA_003713225.1) and the assemblies of a present-day representative of the diploid progenitors species (right, GenBank Assembly Accession GCA_900059795.1 for *C. canephora* and GenBank Assembly Accession GCA_003713205.1 for *C. eugenioides*). The pairwise comparison between the homoeologous chromosomes in the diploid species is shown in the ideograms to the right. In the lower panels, the assemblies of a present-day representative of the diploid progenitors species (middle, GenBank Assembly Accession GCA_900059795.1 for *C. canephora* and GenBank Assembly Accession GCA_003713205.1 for *C. eugenioides*) are compared to the ‘Bourbon’ assembly of this paper (right) and the assembly of *C. arabica* ‘Caturra’ (left, GenBank Assembly Accession GCA_003713225.1). The pairwise comparison between the homoeologous chromosomes in the diploid species is shown in the ideograms to the right. Y-axes indicate million base pairs (Mbp).

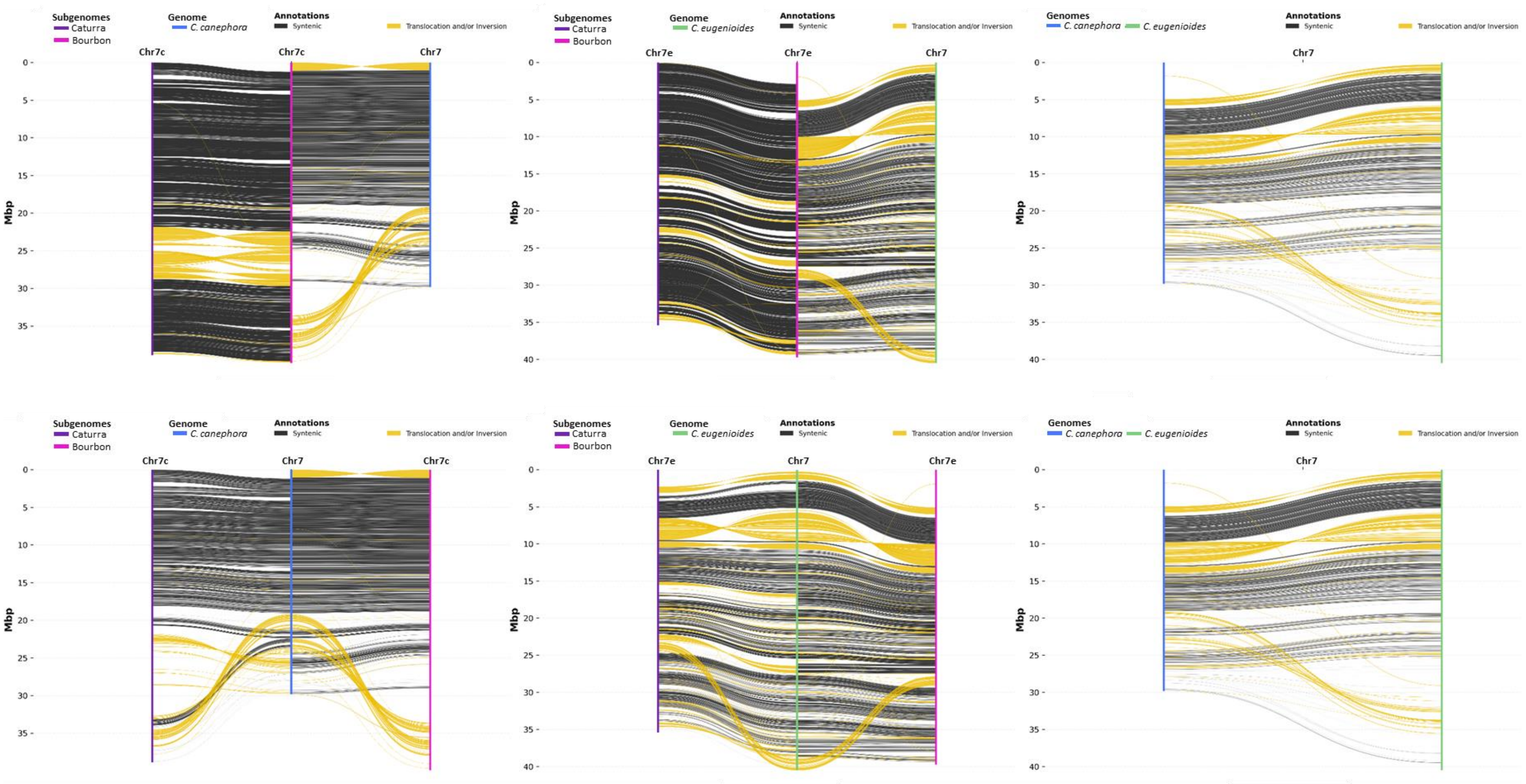

Supplementary Fig. 7. Collinearity among homologous and homoeologous chromosomes 7 (Chr7) among *Coffea* genome assemblies. In the upper panels, the ‘Bourbon’ assembly of this paper (middle) is aligned with the assembly of *C. arabica* ‘Caturra’ (left, GenBank Assembly Accession GCA_003713225.1) and the assemblies of a present-day representative of the diploid progenitors species (right, GenBank Assembly Accession GCA_900059795.1 for *C. canephora* and GenBank Assembly Accession GCA_003713205.1 for *C. eugenioides*). The pairwise comparison between the homoeologous chromosomes in the diploid species is shown in the ideograms to the right. In the lower panels, the assemblies of a present-day representative of the diploid progenitors species (middle, GenBank Assembly Accession GCA_900059795.1 for *C. canephora* and GenBank Assembly Accession GCA_003713205.1 for *C. eugenioides*) are compared to the ‘Bourbon’ assembly of this paper (right) and the assembly of *C. arabica* ‘Caturra’ (left, GenBank Assembly Accession GCA_003713225.1). The pairwise comparison between the homoeologous chromosomes in the diploid species is shown in the ideograms to the right. Y-axes indicate million base pairs (Mbp).

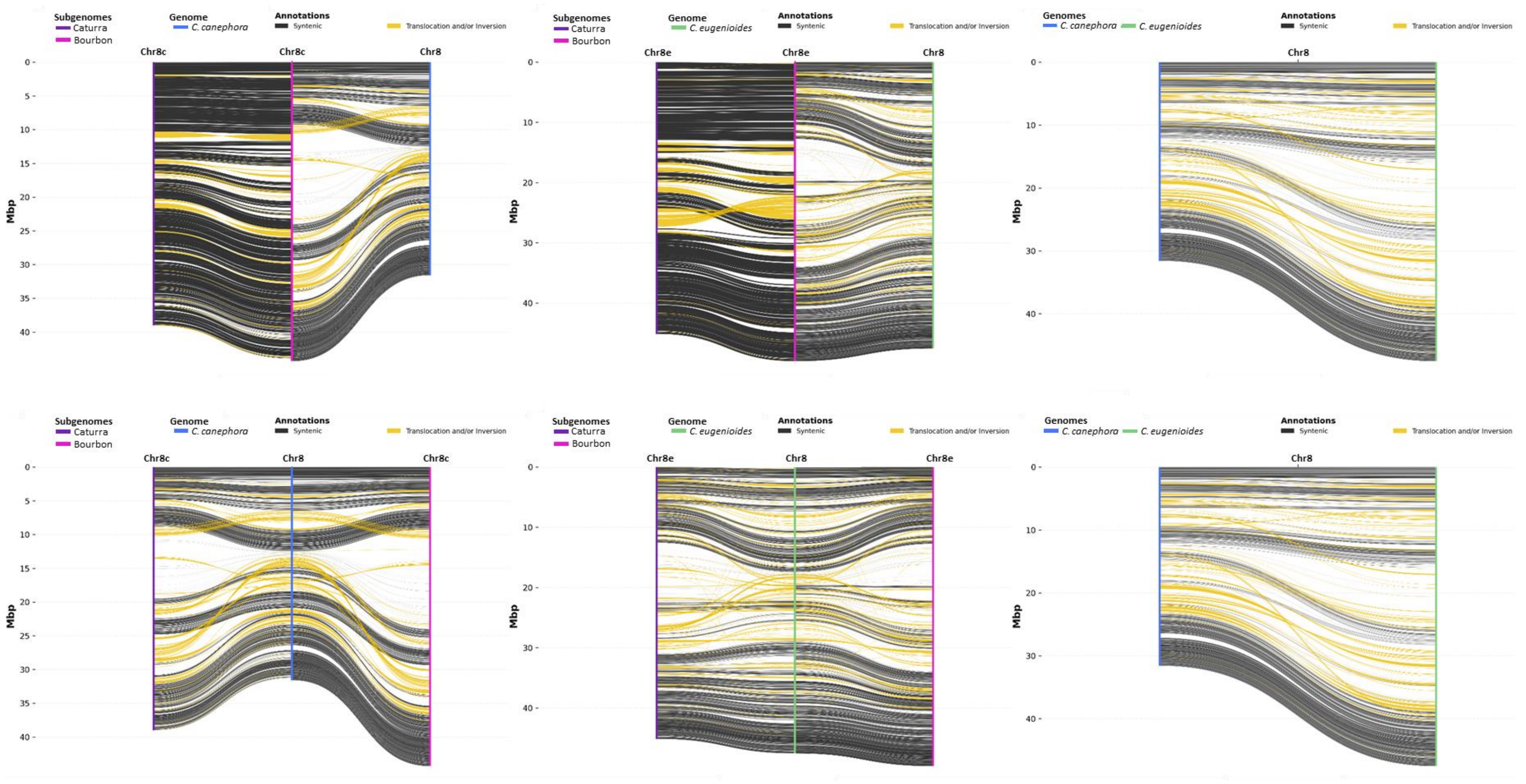

Supplementary Fig. 8. Collinearity among homologous and homoeologous chromosomes 8 (Chr8) among *Coffea* genome assemblies. In the upper panels, the ‘Bourbon’ assembly of this paper (middle) is aligned with the assembly of *C. arabica* ‘Caturra’ (left, GenBank Assembly Accession GCA_003713225.1) and the assemblies of a present-day representative of the diploid progenitors species (right, GenBank Assembly Accession GCA_900059795.1 for *C. canephora* and GenBank Assembly Accession GCA_003713205.1 for *C. eugenioides*). The pairwise comparison between the homoeologous chromosomes in the diploid species is shown in the ideograms to the right. In the lower panels, the assemblies of a present-day representative of the diploid progenitors species (middle, GenBank Assembly Accession GCA_900059795.1 for *C. canephora* and GenBank Assembly Accession GCA_003713205.1 for *C. eugenioides*) are compared to the ‘Bourbon’ assembly of this paper (right) and the assembly of *C. arabica* ‘Caturra’ (left, GenBank Assembly Accession GCA_003713225.1). The pairwise comparison between the homoeologous chromosomes in the diploid species is shown in the ideograms to the right. Y-axes indicate million base pairs (Mbp).

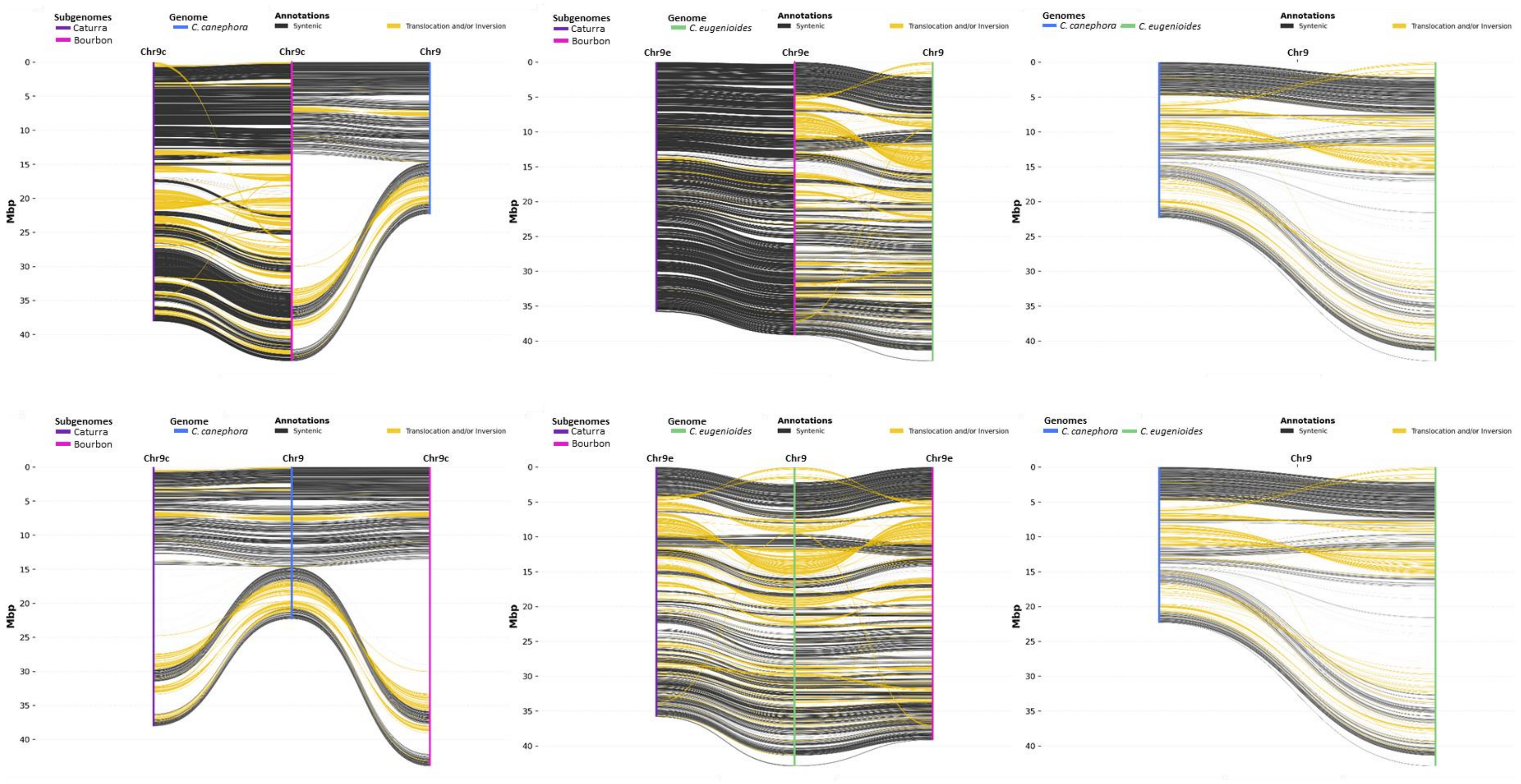

Supplementary Fig. 9. Collinearity among homologous and homoeologous chromosomes 9 (Chr9) among *Coffea* genome assemblies. In the upper panels, the ‘Bourbon’ assembly of this paper (middle) is aligned with the assembly of *C. arabica* ‘Caturra’ (left, GenBank Assembly Accession GCA_003713225.1) and the assemblies of a present-day representative of the diploid progenitors species (right, GenBank Assembly Accession GCA_900059795.1 for *C. canephora* and GenBank Assembly Accession GCA_003713205.1 for *C. eugenioides*). The pairwise comparison between the homoeologous chromosomes in the diploid species is shown in the ideograms to the right. In the lower panels, the assemblies of a present-day representative of the diploid progenitors species (middle, GenBank Assembly Accession GCA_900059795.1 for *C. canephora* and GenBank Assembly Accession GCA_003713205.1 for *C. eugenioides*) are compared to the ‘Bourbon’ assembly of this paper (right) and the assembly of *C. arabica* ‘Caturra’ (left, GenBank Assembly Accession GCA_003713225.1). The pairwise comparison between the homoeologous chromosomes in the diploid species is shown in the ideograms to the right. Y-axes indicate million base pairs (Mbp).

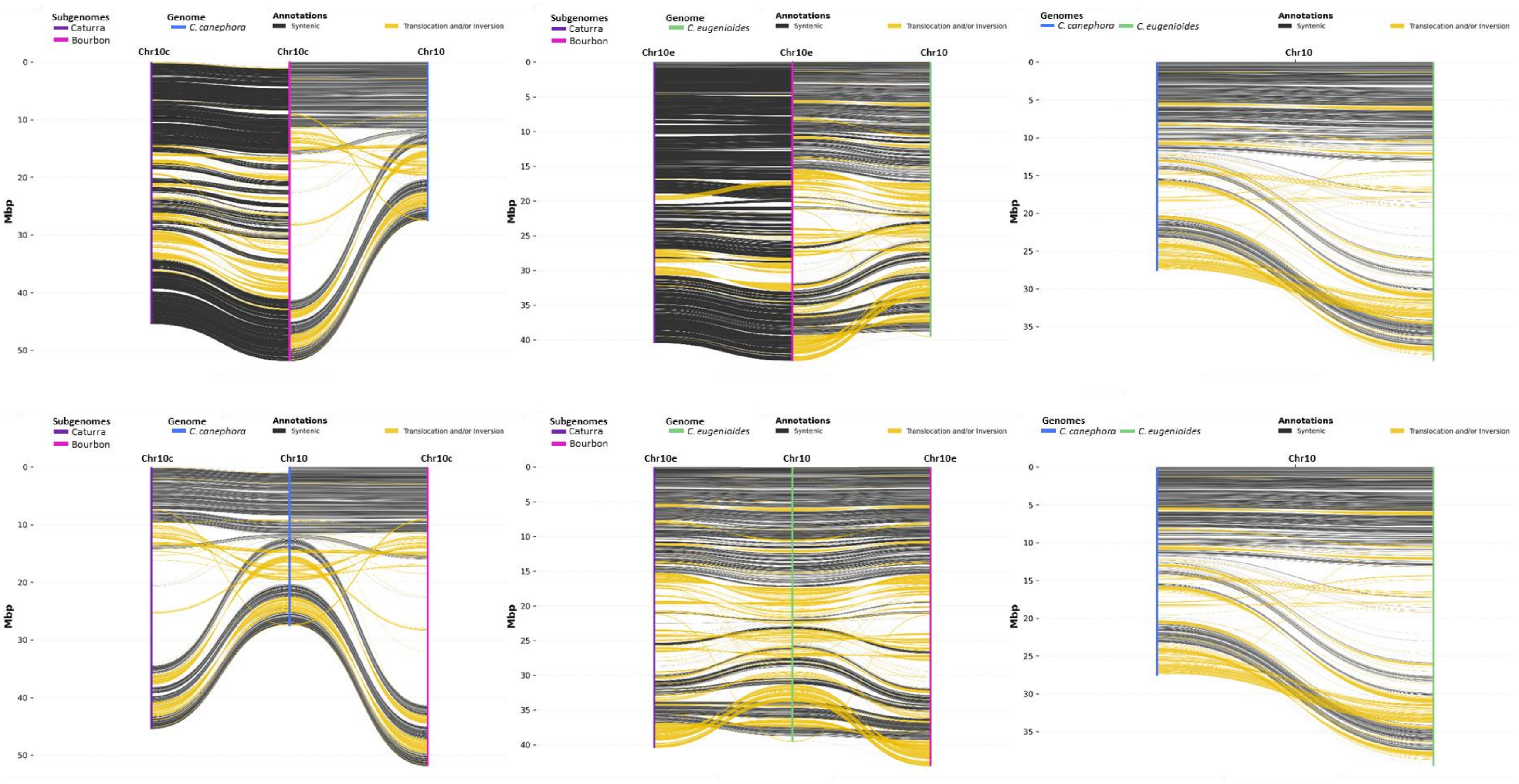

Supplementary Fig. 10. Collinearity among homologous and homoeologous chromosomes 10 (Chr10) among *Coffea* genome assemblies. In the upper panels, the ‘Bourbon’ assembly of this paper (middle) is aligned with the assembly of *C. arabica* ‘Caturra’ (left, GenBank Assembly Accession GCA_003713225.1) and the assemblies of a present-day representative of the diploid progenitors species (right, GenBank Assembly Accession GCA_900059795.1 for *C. canephora* and GenBank Assembly Accession GCA_003713205.1 for *C. eugenioides*). The pairwise comparison between the homoeologous chromosomes in the diploid species is shown in the ideograms to the right. In the lower panels, the assemblies of a present-day representative of the diploid progenitors species (middle, GenBank Assembly Accession GCA_900059795.1 for *C. canephora* and GenBank Assembly Accession GCA_003713205.1 for *C. eugenioides*) are compared to the ‘Bourbon’ assembly of this paper (right) and the assembly of *C. arabica* ‘Caturra’ (left, GenBank Assembly Accession GCA_003713225.1). The pairwise comparison between the homoeologous chromosomes in the diploid species is shown in the ideograms to the right. Y-axes indicate million base pairs (Mbp).

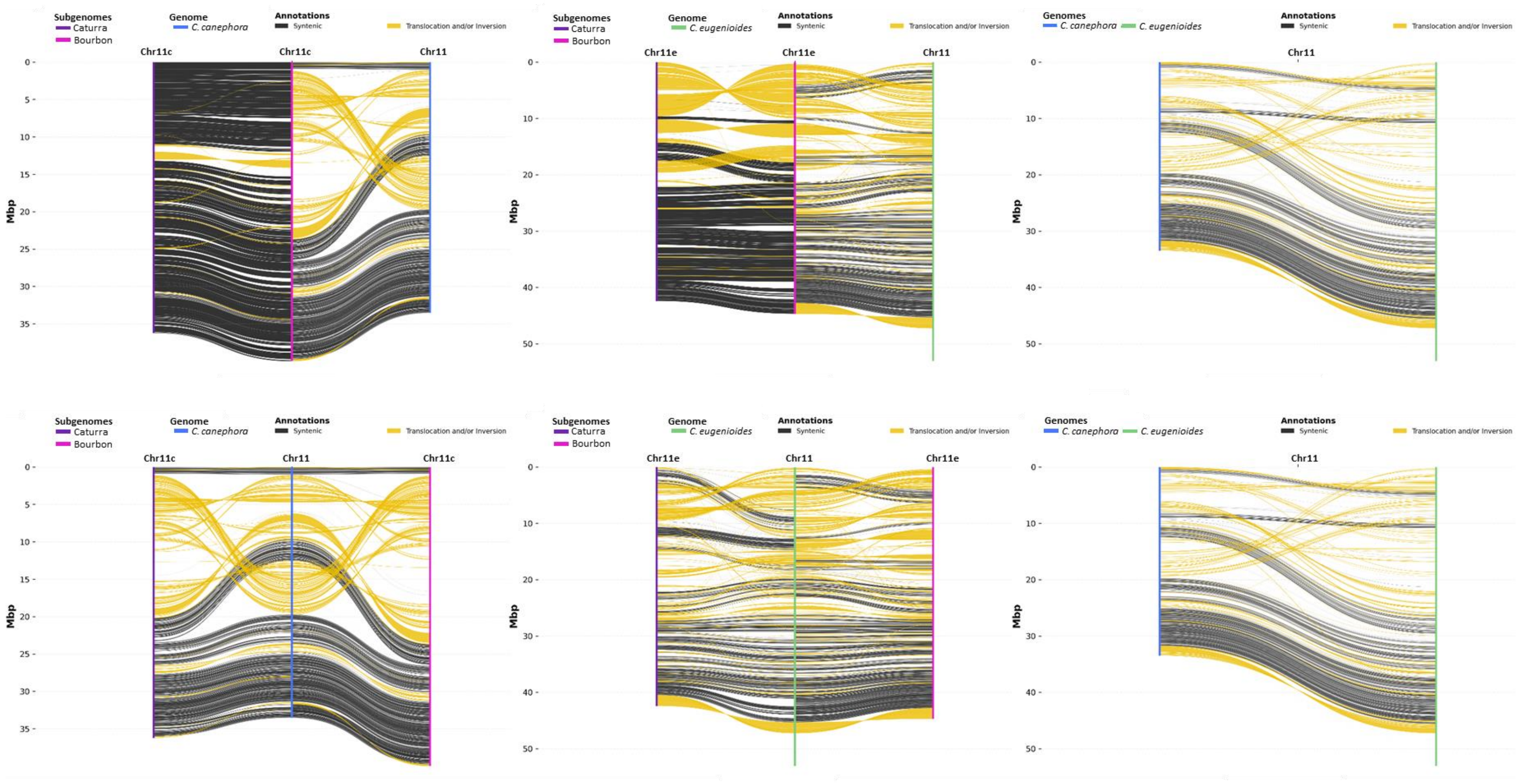

Supplementary Fig. 11. Collinearity among homologous and homoeologous chromosomes 11 (Chr11) among *Coffea* genome assemblies. In the upper panels, the ‘Bourbon’ assembly of this paper (middle) is aligned with the assembly of *C. arabica* ‘Caturra’ (left, GenBank Assembly Accession GCA_003713225.1) and the assemblies of a present-day representative of the diploid progenitors species (right, GenBank Assembly Accession GCA_900059795.1 for *C. canephora* and GenBank Assembly Accession GCA_003713205.1 for *C. eugenioides*). The pairwise comparison between the homoeologous chromosomes in the diploid species is shown in the ideograms to the right. In the lower panels, the assemblies of a present-day representative of the diploid progenitors species (middle, GenBank Assembly Accession GCA_900059795.1 for *C. canephora* and GenBank Assembly Accession GCA_003713205.1 for *C. eugenioides*) are compared to the ‘Bourbon’ assembly of this paper (right) and the assembly of *C. arabica* ‘Caturra’ (left, GenBank Assembly Accession GCA_003713225.1). The pairwise comparison between the homoeologous chromosomes in the diploid species is shown in the ideograms to the right. Y-axes indicate million base pairs (Mbp).

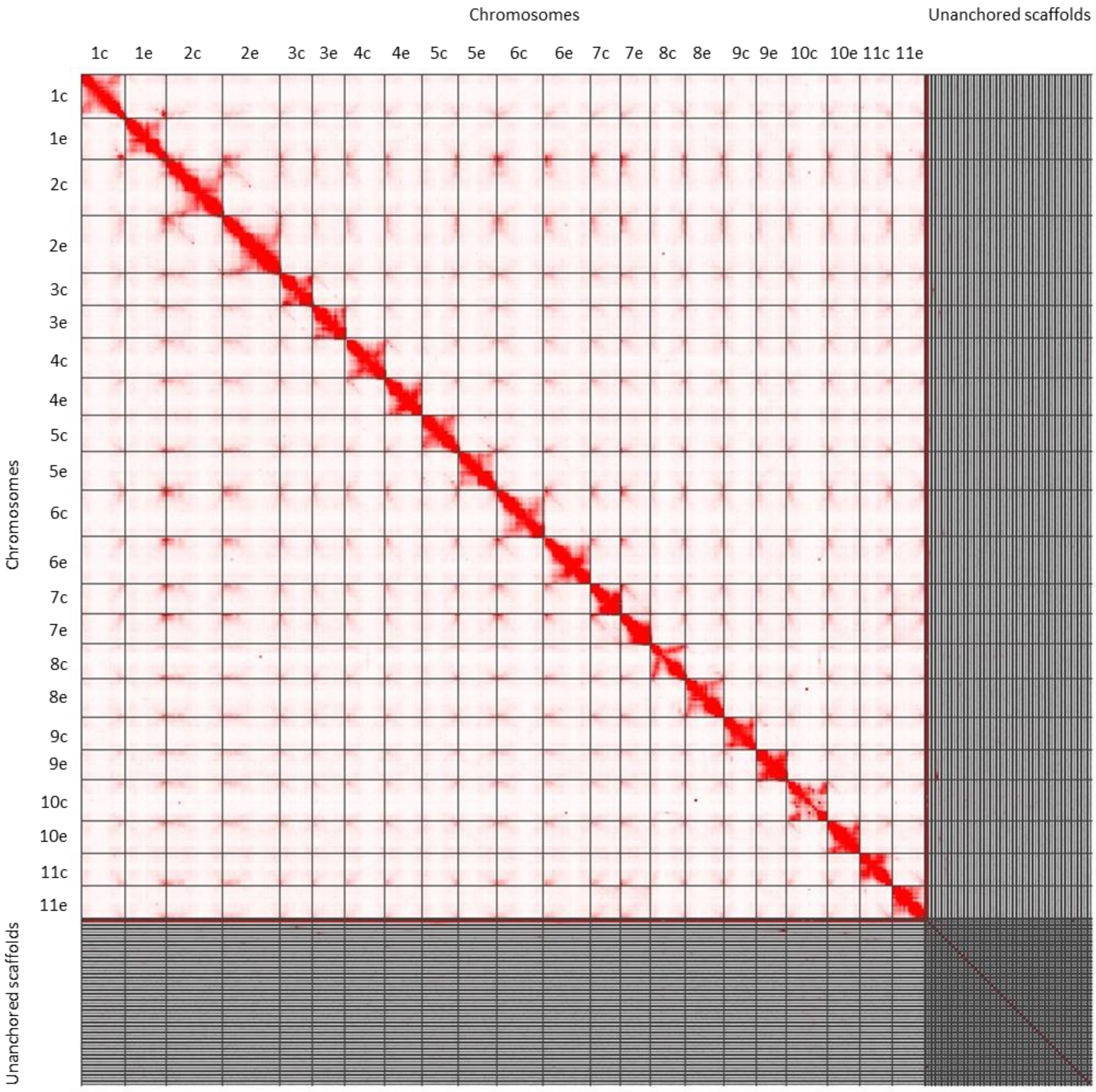

Supplementary Fig. 12. Hi-C contact map. The interactions map shows consistency between the intrachromosomal order and orientation of the 'Bourbon' genomic sequence and the frequency of chromatin interactions in the nuclei of 'Bourbon' young leaves. Colour intensity is proportional to the interaction frequency.

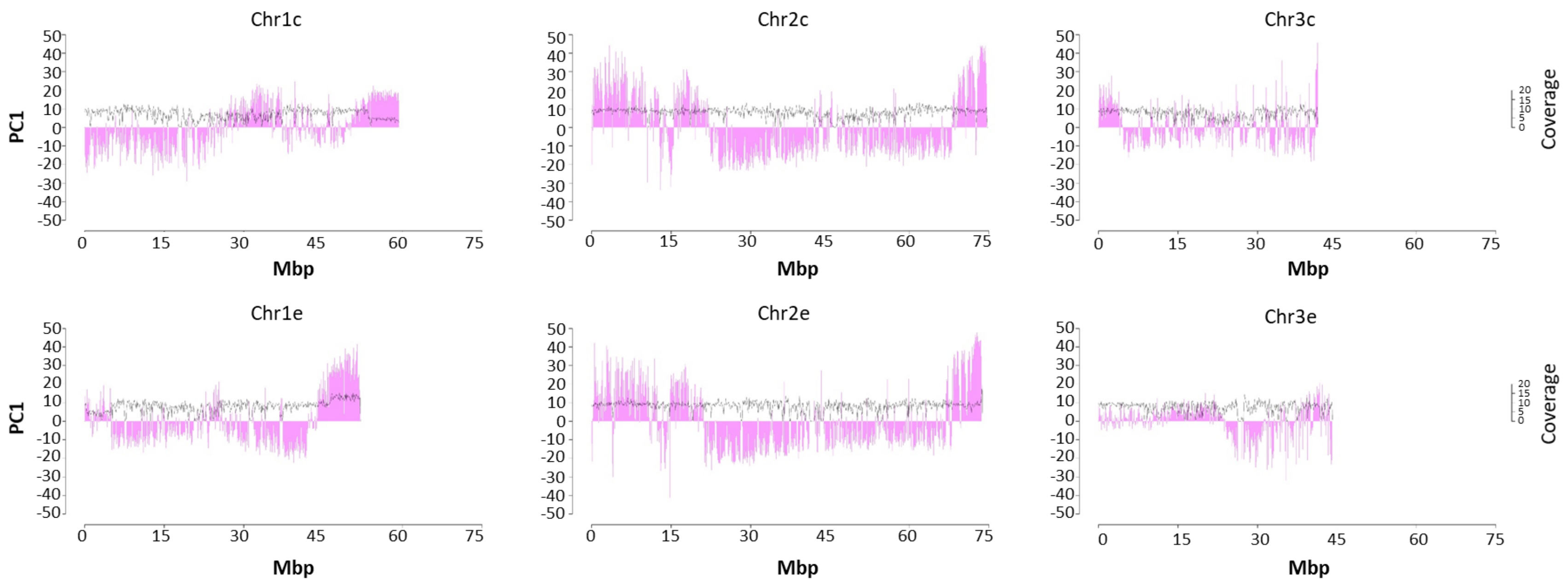

Supplementary Fig. 13. Chromatin organisation in *C. arabica* chromosomes Chr1, Chr2 and Chr3. The pink histogram shows the first principal component (PC1) indicating the assignment to either the A (positive values on the primary y-axis) or to the B compartment (negative values on the primary y-axis). PC1 values were calculated from full chromosome distance-normalized interaction matrices at 50 Kb resolution in non-overlapping and fixed genomic windows of 100 Kb. The black line represents Hi-C read coverage (secondary y-axis). X-axes indicate million base pairs (Mbp). Source data are provided as a Source Data file.

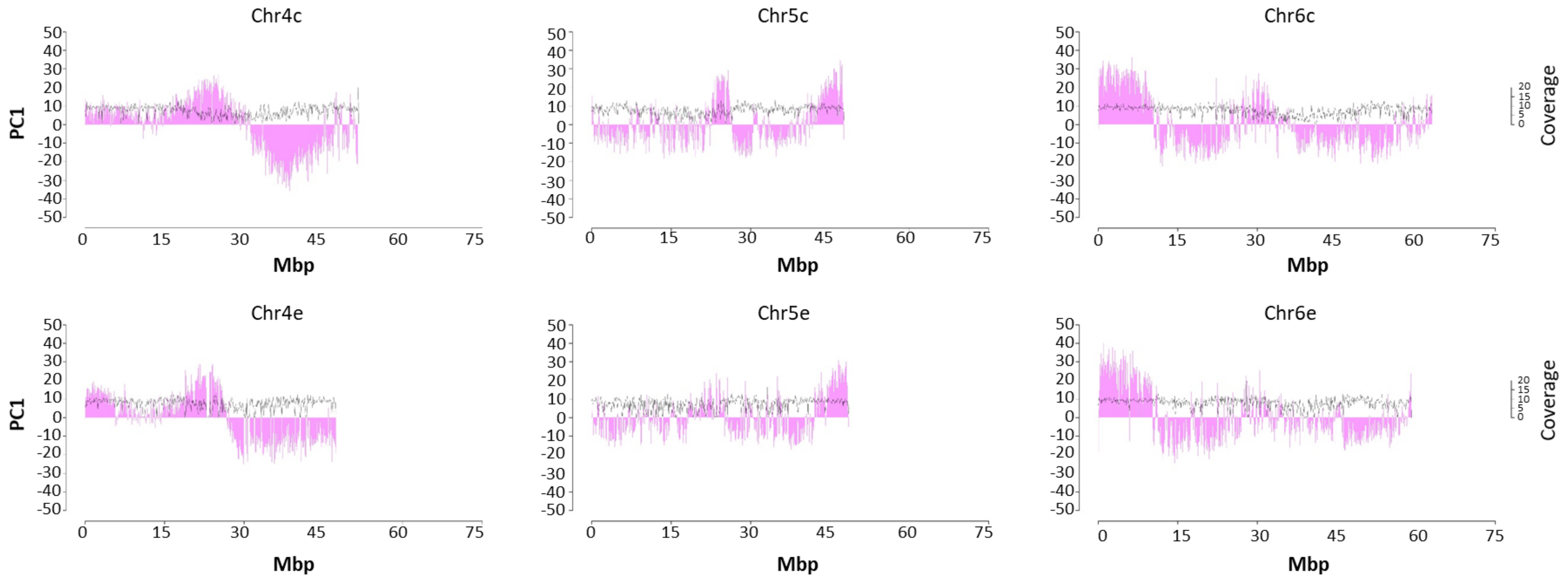

Supplementary Fig. 14. Chromatin organisation in *C. arabica* chromosomes Chr4, Chr5 and Chr6. The pink histogram shows the first principal component (PC1) indicating the assignment to either the A (positive values on the primary y-axis) or to the B compartment (negative values on the primary y-axis). PC1 values were calculated from full chromosome distance-normalized interaction matrices at 50 Kb resolution in non-overlapping and fixed genomic windows of 100 Kb. The black line represents Hi-C read coverage (secondary y-axis). X-axes indicate million base pairs (Mbp). Source data are provided as a Source Data file.

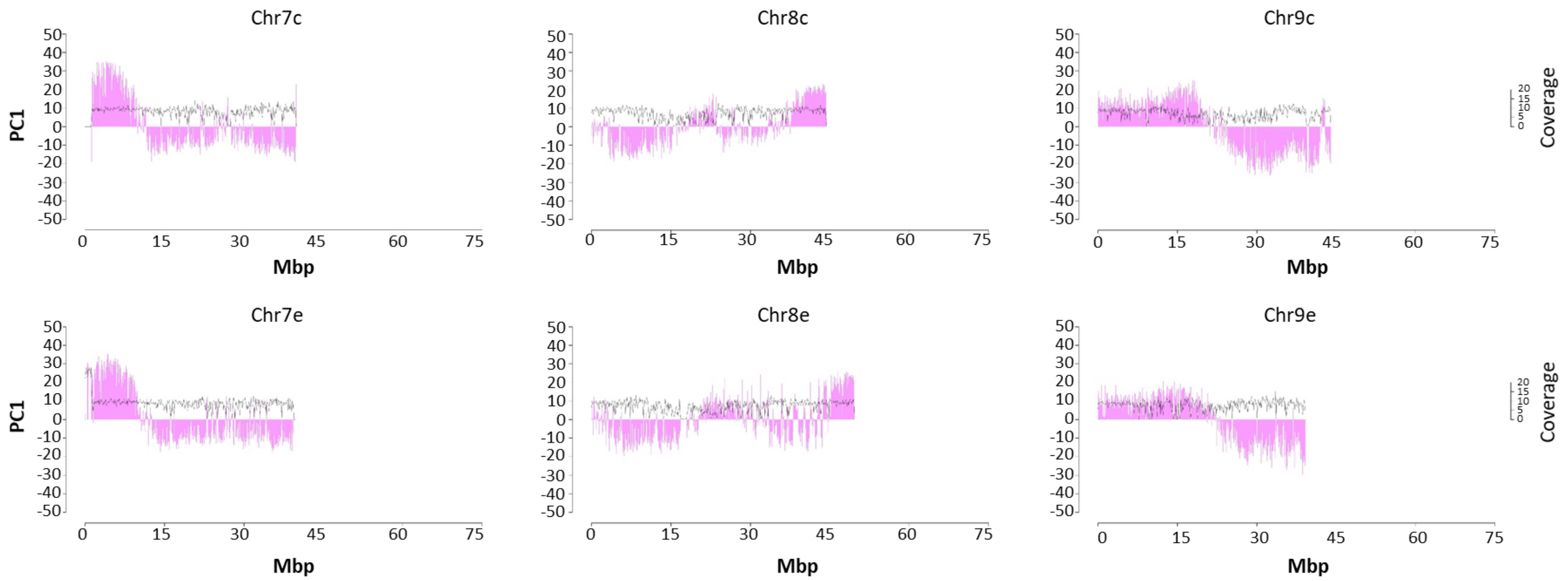

Supplementary Fig. 15. Chromatin organisation in *C. arabica* chromosomes Chr7, Chr8 and Chr9. The pink histogram shows the first principal component (PC1) indicating the assignment to either the A (positive values on the primary y-axis) or to the B compartment (negative values on the primary y-axis). PC1 values were calculated from full chromosome distance-normalized interaction matrices at 50 Kb resolution in non-overlapping and fixed genomic windows of 100 Kb. The black line represents Hi-C read coverage (secondary y-axis). X-axes indicate million base pairs (Mbp). Source data are provided as a Source Data file.

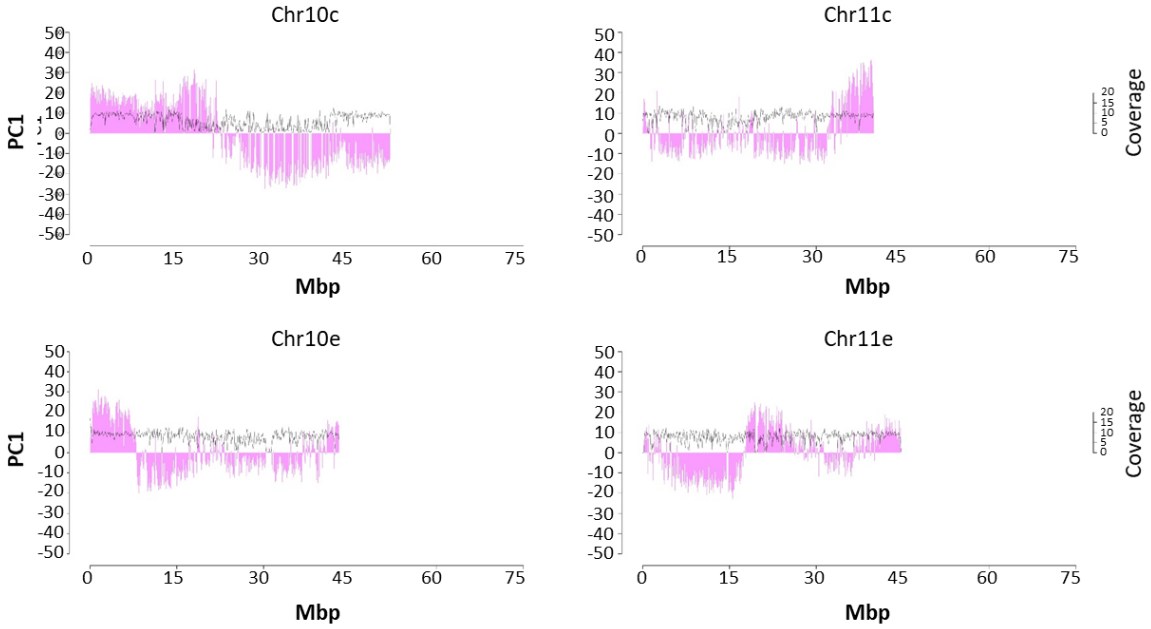

Supplementary Fig. 16. Chromatin organisation in *C. arabica* chromosomes Chr10 and Chr11. The pink histogram shows the first principal component (PC1) indicating the assignment to either the A (positive values on the primary y-axis) or to the B compartment (negative values on the primary y-axis). PC1 values were calculated from full chromosome distance-normalized interaction matrices at 50 Kb resolution in non-overlapping and fixed genomic windows of 100 Kb. The black line represents Hi-C read coverage (secondary y-axis). X-axes indicate million base pairs (Mbp). Source data are provided as a Source Data file.

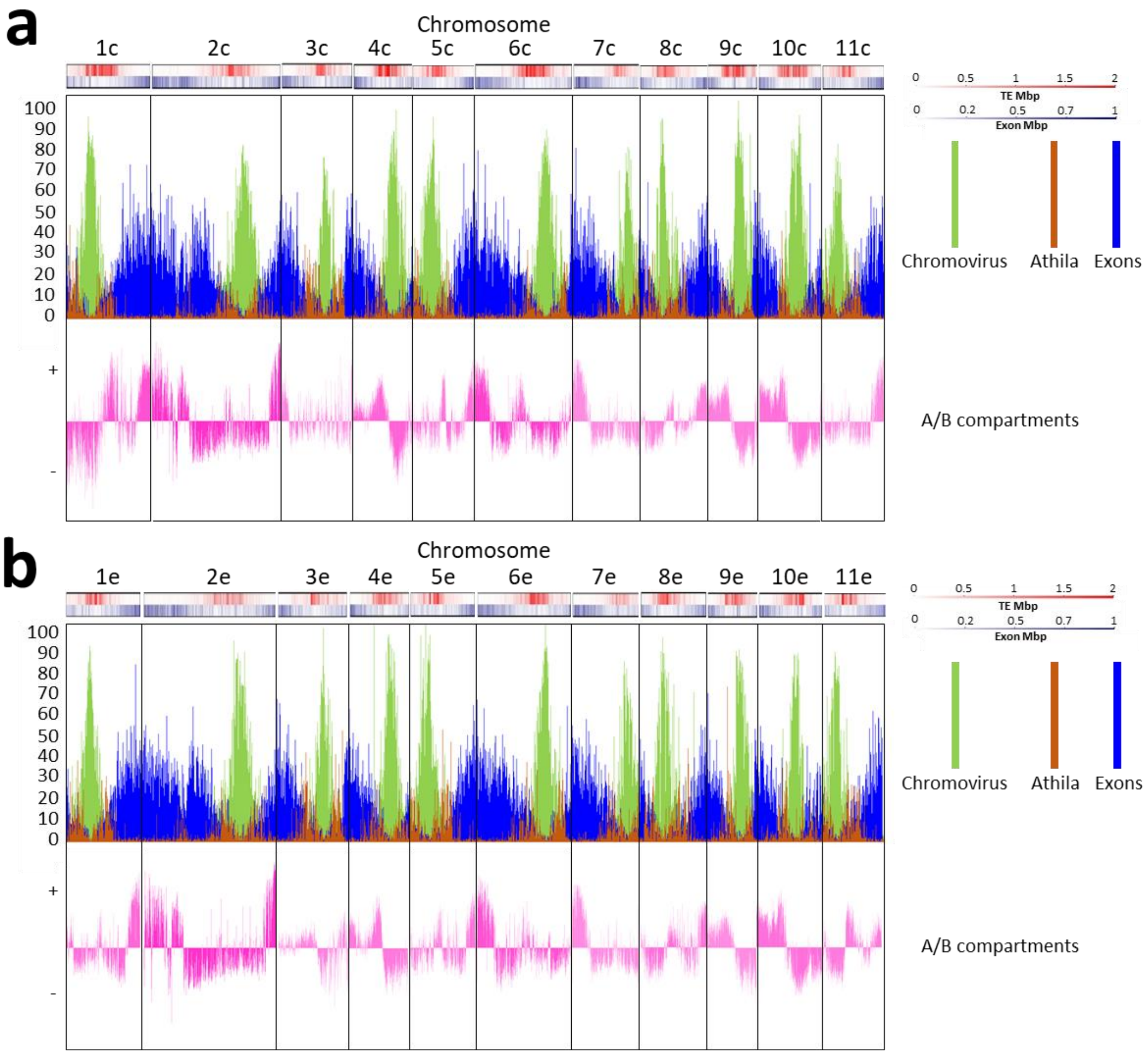

Supplementary Fig. 17. Structure of *C. arabica* chromosomes. a Canephora subgenome. **b** Eugenioides subgenome. In both panels, gene and transposable elements (TE) densities across 4,467 non-overlapping genomic windows corresponding to 100 Kb of non-repetitive DNA are shown as blue-to-white and red-to-white heatmaps, respectively, consistent with the illustration in Fig. 1c. Olive and brown histograms show, respectively, the percentage of base pairs included in satellite repeat arrays formed by Chromovirus derived-sequences and in Athila retroelements across 4,467 non-overlapping genomic windows containing 100 Kb of non-repetitive DNA (2,212 in the canephora subgenome and 2,255 in the eugenioides subgenome). A/B compartments were predicted using non-overlapping and fixed genomic windows of 100 Kb. Source data are provided as a Source Data file.

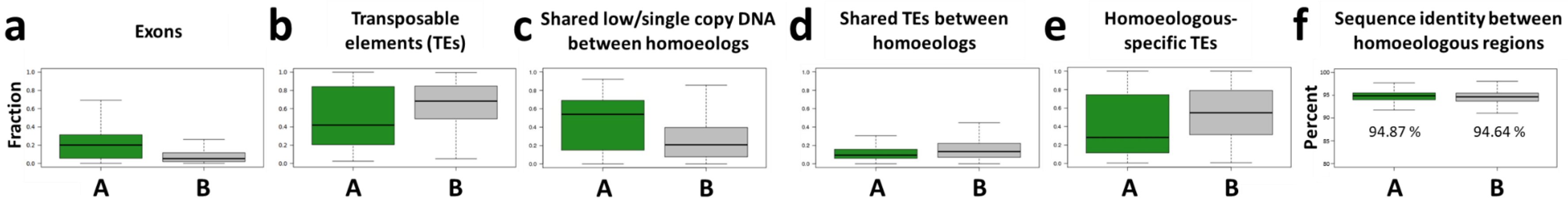

Supplementary Fig. 18. Enrichment of A/B compartments in different genomic features. **a** Gene content. **b** Transposable elements (TEs). **c** Shared low/single copy DNA between homoeologs. **d** Shared TEs between homoeologs. **e** Homoeologous-specific TEs. **f** Sequence identity between homoeologous regions. In all panels, box plots show variation among genomic windows belonging to either the A or the B compartment. All box plot distributions showed statistically significant differences between compartments using a two-sided Wilcoxon test. Numbers indicating the median values are reported in **f**. Regions considered in **f** correspond to those considered in both **c** and **d**. Boxes indicate the first and third quartiles, the horizontal line within the boxes indicates the median and the whiskers indicate ±1.5 × interquartile range. Source data are provided as a Source Data file.

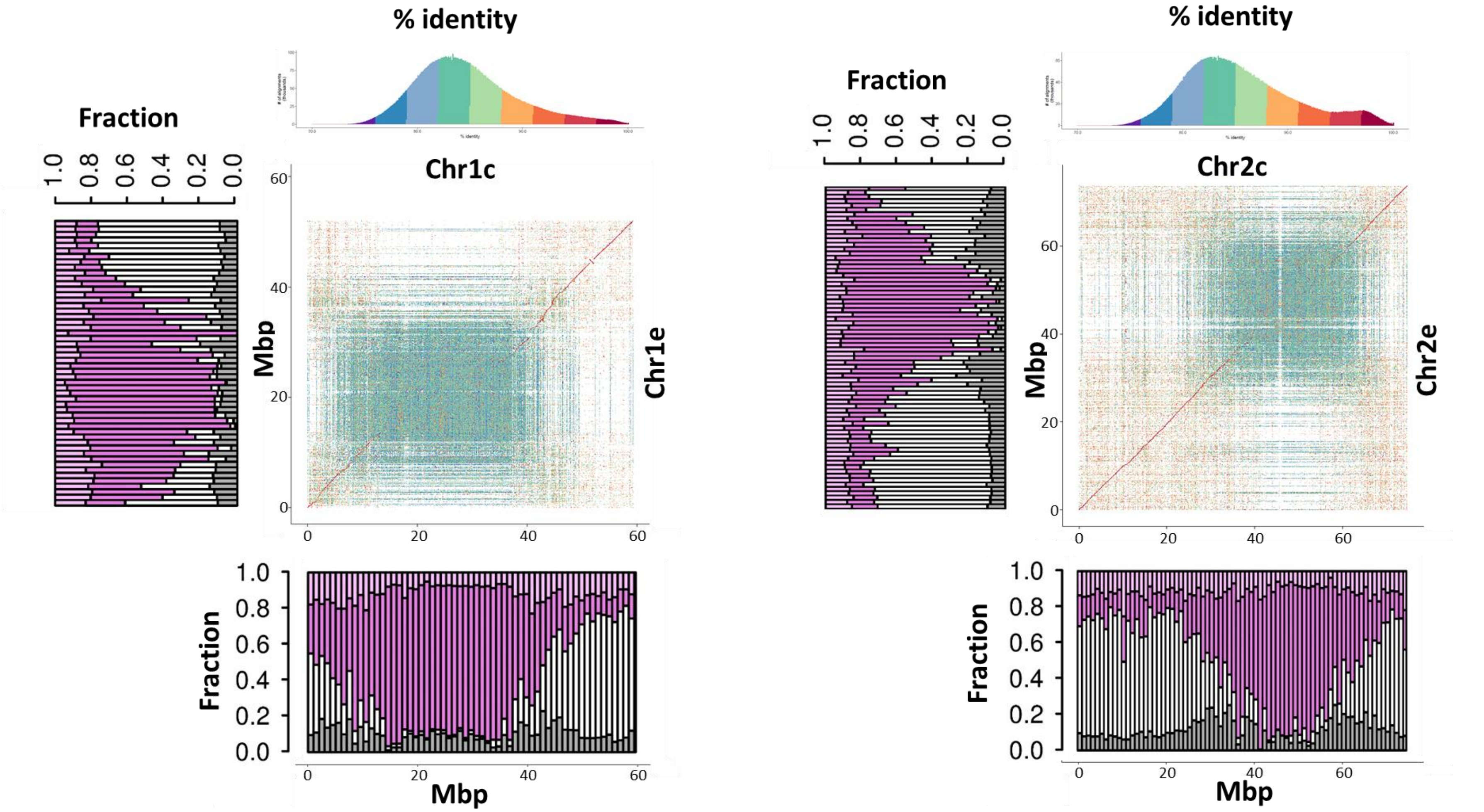

Supplementary Fig. 19. Sequence identity and structural variation between homoeologous chromosomes (Chr1c vs Chr1e and Chr2c vs Chr2e). Each dot represents sequence alignments with >70 % of identity between non-overlapping 2 Kb windows. The colour of each dot represents the % of sequence identity. Bar plots represent the fraction of nucleotides shared between (white and gray) or private to (pink and magenta) the homoeologs. These categories are further sorted into the fraction of nucleotides in annotated transposable elements (gray and magenta) and in non-repetitive DNA (white). The pink stack includes not shared low-copy DNA as well as other DNA tracts that are not annotated as transposable elements. The y-axis indicating the chromosomal coordinates in million base pairs (Mbp) of each homoeolog refers to both the bar plot and the sequence identity plot. Source data are provided as a Source Data file.

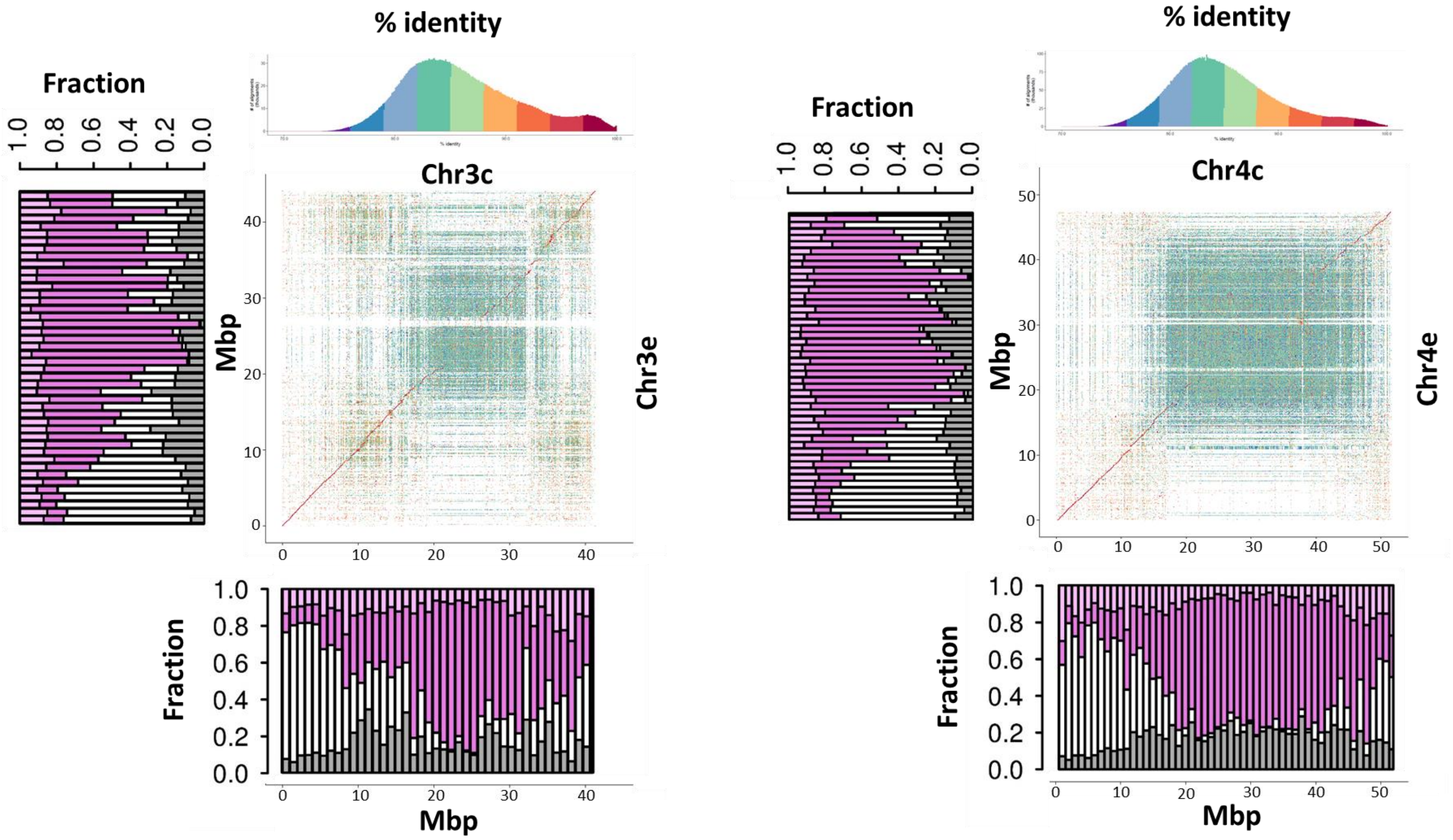

Supplementary Fig. 20. Sequence identity and structural variation between homoeologous chromosomes (Chr3c vs Chr3e and Chr4c vs Chr4e). Each dot represents sequence alignments with >70 % of identity between non-overlapping 2 Kb windows. The colour of each dot represents the % of sequence identity. Bar plots represent the fraction of nucleotides shared between (white and gray) or private to (pink and magenta) the homoeologs. These categories are further sorted into the fraction of nucleotides in annotated transposable elements (gray and magenta) and in non-repetitive DNA (white). The pink stack includes not shared low-copy DNA as well as other DNA tracts that are not annotated as transposable elements. The y-axis indicating the chromosomal coordinates in million base pairs (Mbp) of each homoeolog refers to both the bar plot and the sequence identity plot. Source data are provided as a Source Data file.

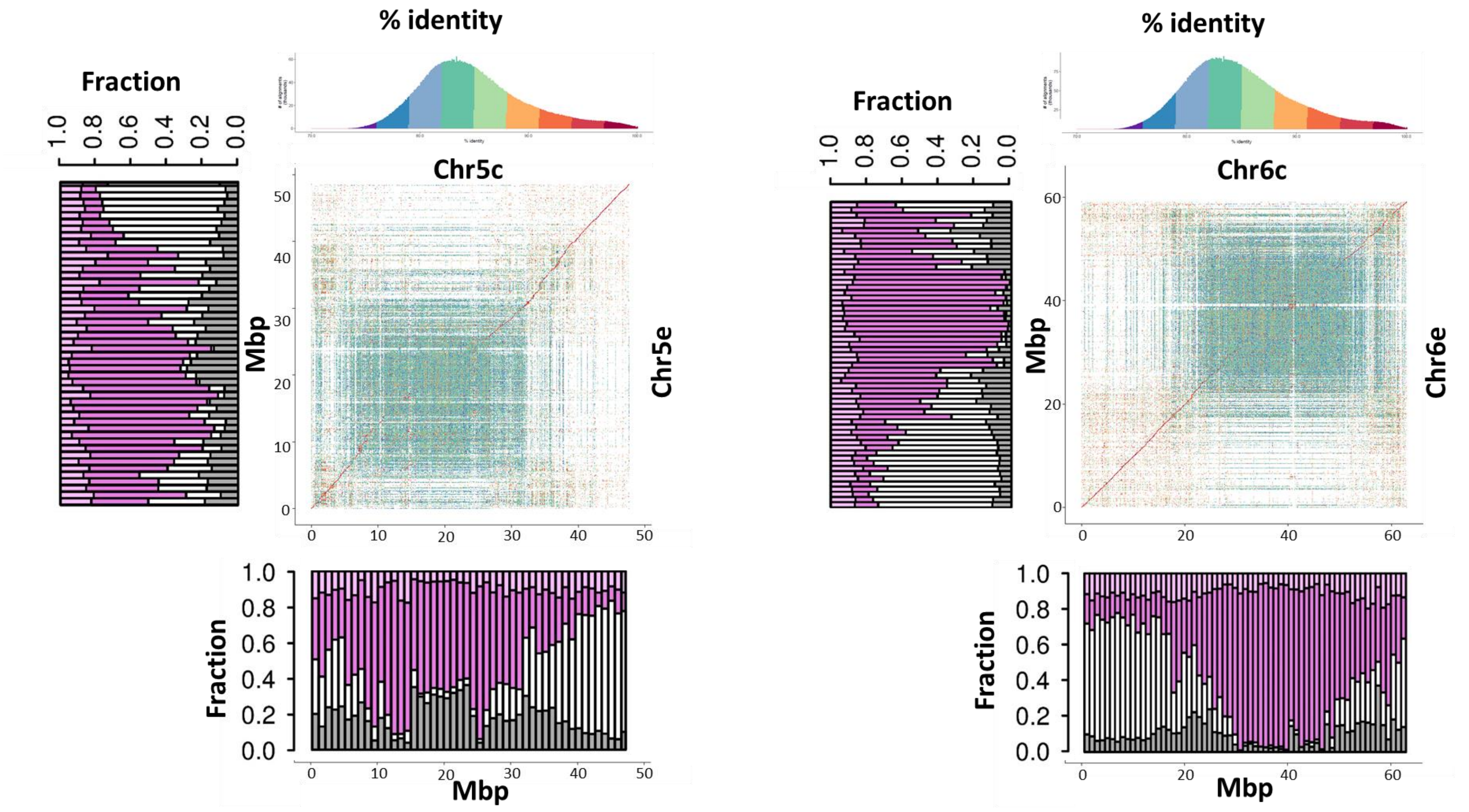

Supplementary Fig. 21. Sequence identity and structural variation between homoeologous chromosomes (Chr5c vs Chr5e and Chr6c vs Chr6e). Each dot represents sequence alignments with >70 % of identity between non-overlapping 2 Kb windows. The colour of each dot represents the % of sequence identity. Bar plots represent the fraction of nucleotides shared between (white and gray) or private to (pink and magenta) the homoeologs. These categories are further sorted into the fraction of nucleotides in annotated transposable elements (gray and magenta) and in non-repetitive DNA (white). The pink stack includes not shared low-copy DNA as well as other DNA tracts that are not annotated as transposable elements. The y-axis indicating the chromosomal coordinates in million base pairs (Mbp) of each homoeolog refers to both the bar plot and the sequence identity plot. Source data are provided as a Source Data file.

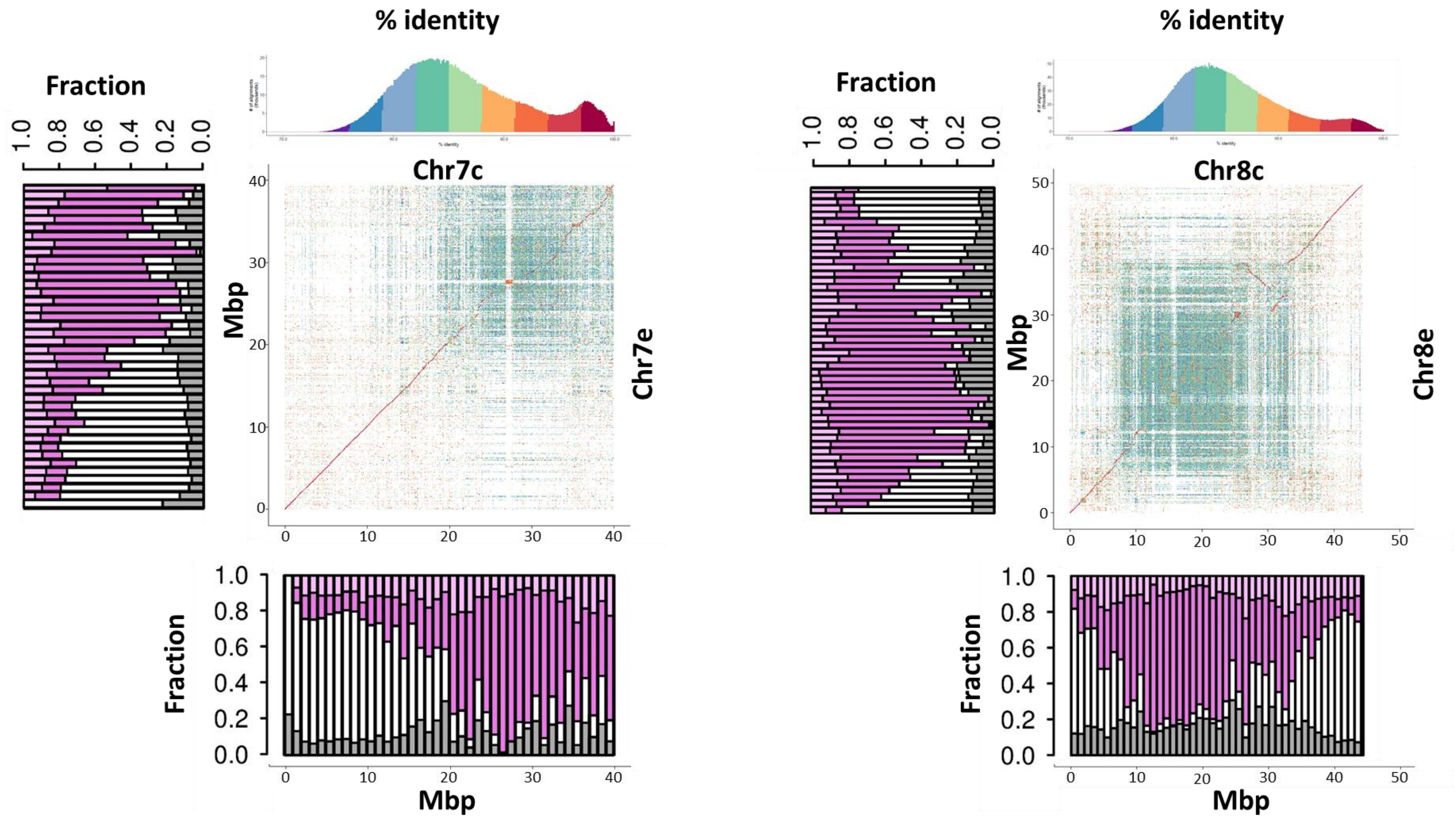

Supplementary Fig. 22. Sequence identity and structural variation between homoeologous chromosomes (Chr7c vs Chr7e and Chr8c vs Chr8e). Each dot represents sequence alignments with >70 % of identity between non-overlapping 2 Kb windows. The colour of each dot represents the % of sequence identity. Bar plots represent the fraction of nucleotides shared between (white and gray) or private to (pink and magenta) the homoeologs. These categories are further sorted into the fraction of nucleotides in annotated transposable elements (gray and magenta) and in non-repetitive DNA (white). The pink stack includes not shared low-copy DNA as well as other DNA tracts that are not annotated as transposable elements. The y-axis indicating the chromosomal coordinates in million base pairs (Mbp) of each homoeolog refers to both the bar plot and the sequence identity plot. Source data are provided as a Source Data file.

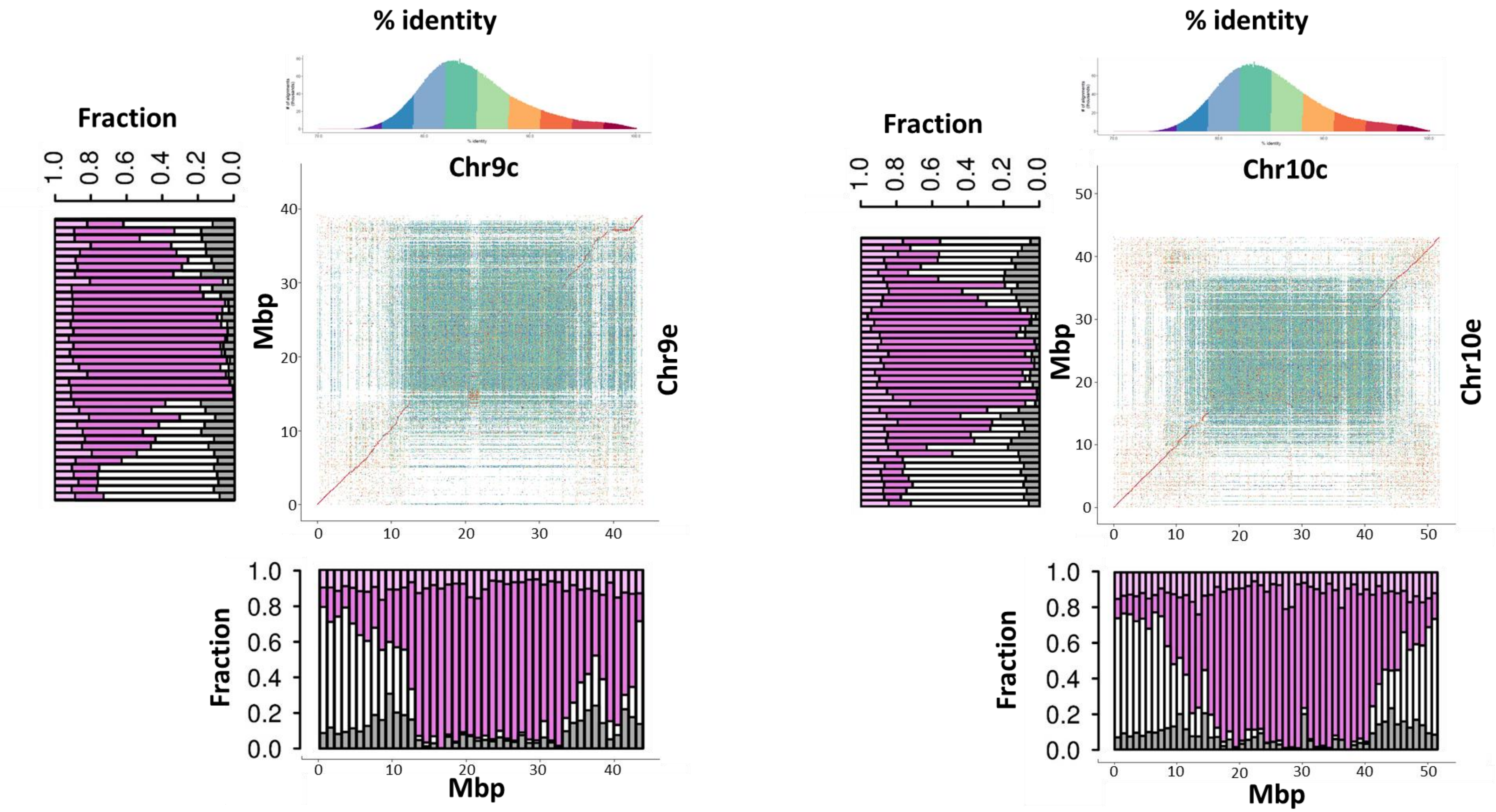

Supplementary Fig. 23. Sequence identity and structural variation between homoeologous chromosomes (Chr9c vs Chr9e and Chr10c vs Chr10e). Each dot represents sequence alignments with >70 % of identity between non-overlapping 2 Kb windows. The colour of each dot represents the % of sequence identity. Bar plots represent the fraction of nucleotides shared between (white and gray) or private to (pink and magenta) the homoeologs. These categories are further sorted into the fraction of nucleotides in annotated transposable elements (gray and magenta) and in non-repetitive DNA (white). The pink stack includes not shared low-copy DNA as well as other DNA tracts that are not annotated as transposable elements. The y-axis indicating the chromosomal coordinates in million base pairs (Mbp) of each homoeolog refers to both the bar plot and the sequence identity plot. Source data are provided as a Source Data file.

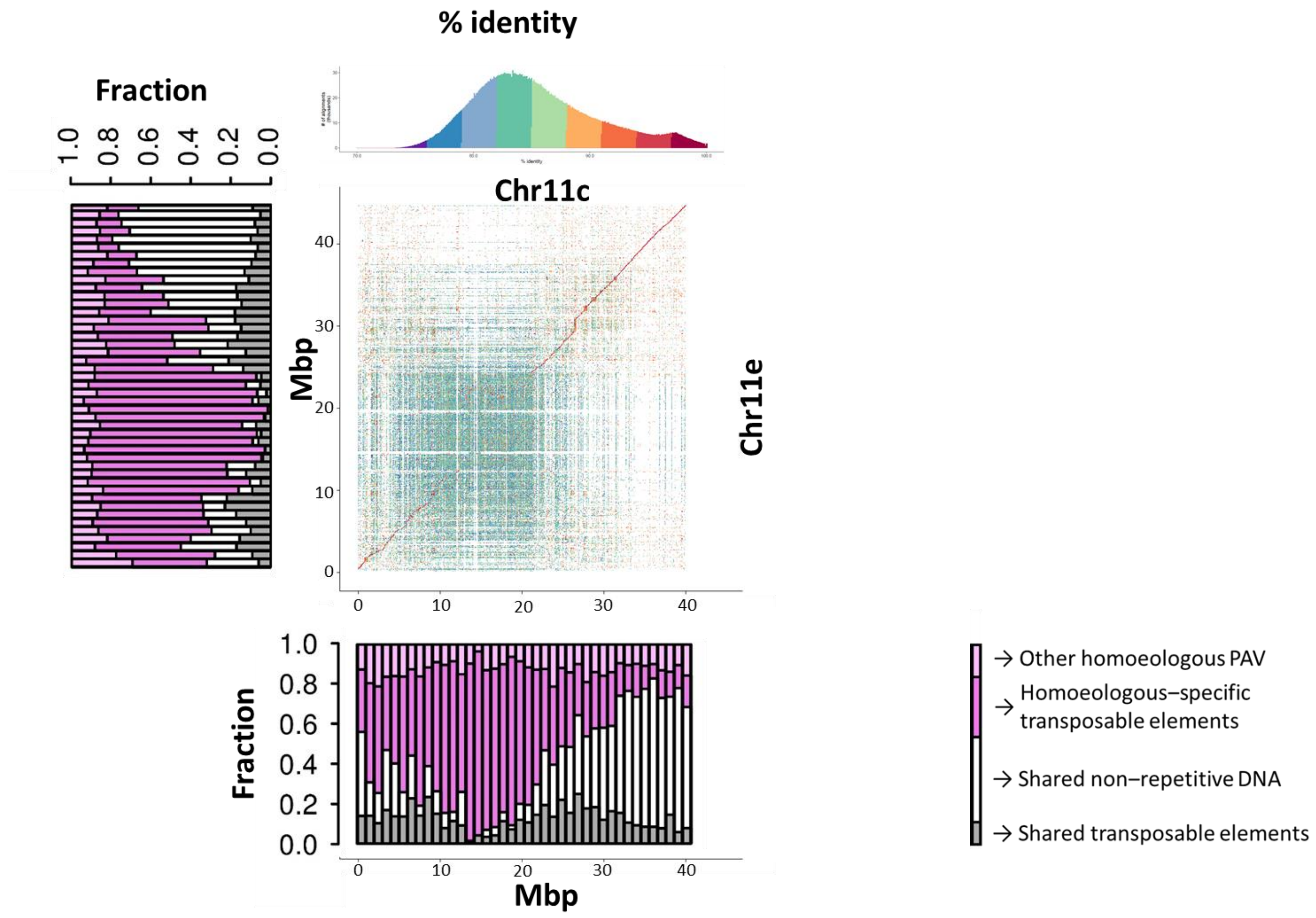

Supplementary Fig. 24. Sequence identity and structural variation between homoeologous chromosomes (Chr11c vs Chr11e). Each dot represents sequence alignments with >70 % of identity between non-overlapping 2 Kb windows. The colour of each dot represents the % of sequence identity. Bar plots represent the fraction of nucleotides shared between (white and gray) or private to (pink and magenta) the homoeologs. These categories are further sorted into the fraction of nucleotides in annotated transposable elements (gray and magenta) and in non-repetitive DNA (white). The pink stack includes not shared low-copy DNA as well as other DNA tracts that are not annotated as transposable elements. The y-axis indicating the chromosomal coordinates in million base pairs (Mbp) of each homoeolog refers to both the bar plot and the sequence identity plot. Source data are provided as a Source Data file.

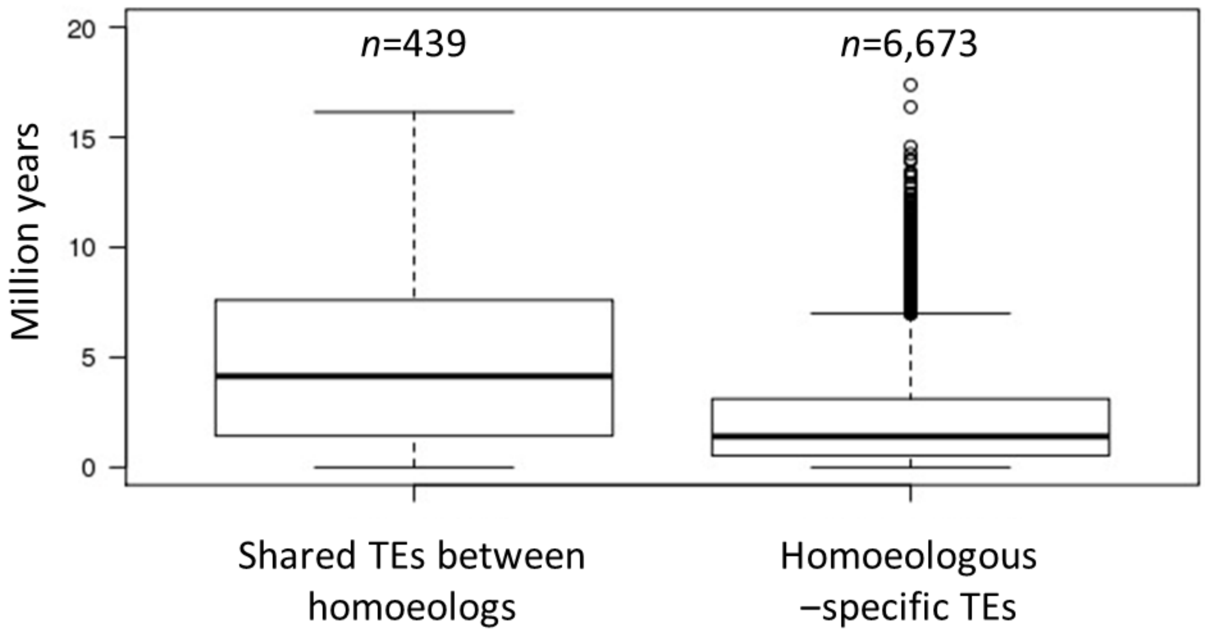

Supplementary Fig. 25. Box plot distribution of the estimated age of LTR-retrotransposon insertions that occurred before and after speciation of the *C. arabica* diploid ancestors. LTR-retrotransposons for elements that are shared between homoeologs (showing conserved genomic position of the element relative to the low/single copy flanking DNA and to the orthologous genes in the canephora and eugenioides homoeologs and the same target site duplication on both homoeologs) and for elements that are private to either homoeolog. The estimated age was calculated based on intraelement LTR sequence divergence and a mutation rate of 1.3×10^{-8} per site per year used for annuals and divided by 3 years per generation (duration of the juvenile phase). Numbers above each box (*n*=) indicate the number of transposable elements (TEs) in each category. Boxes indicate the first and third quartiles, the horizontal line within the boxes indicates the median and the whiskers indicate ±1.5 × interquartile range. Source data are provided as a Source Data file.

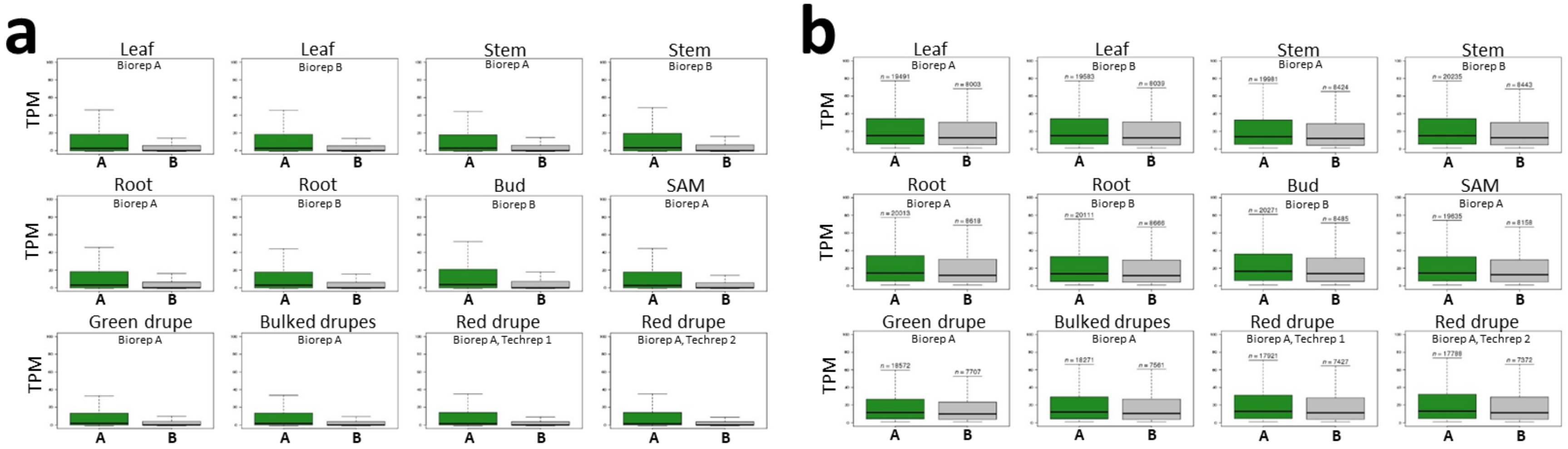

Supplementary Fig. 26. Gene expression levels of genes located in A or B chromatin compartments in different organs. **a** All genes in A (*n*=34,189) or B (*n*=22,930) chromatin compartments. **b** Genes in A or B chromatin compartments with TPM >1 in the organ analyzed. The number of genes with TPM>1 is indicated above each plot (*n*=). In both panels, green box plots show Transcripts Per Million (TPM) distributions for genes located in A-type open chromatin compartments; gray box plots show Transcripts Per Million (TPM) distributions for genes located in B-type compact chromatin compartments. All box plot distributions showed statistically significant differences between compartments using a two-sided Wilcoxon test. BioRep A and B stand for Biological Replicates (i.e. independent RNA extraction from two plants). Techrep 1 and 2 stand for Technical Replicates (i.e. independent RNA extraction from specimens sampled from the same plant). SAM stands for Shoot Apical Meristem. Bulked drupes stand for multiple drupes at different ripening stages sampled on the same plant. Boxes indicate the first and third quartiles, the horizontal line within the boxes indicates the median and the whiskers indicate ±1.5 × interquartile range. Source data are provided as a Source Data file.

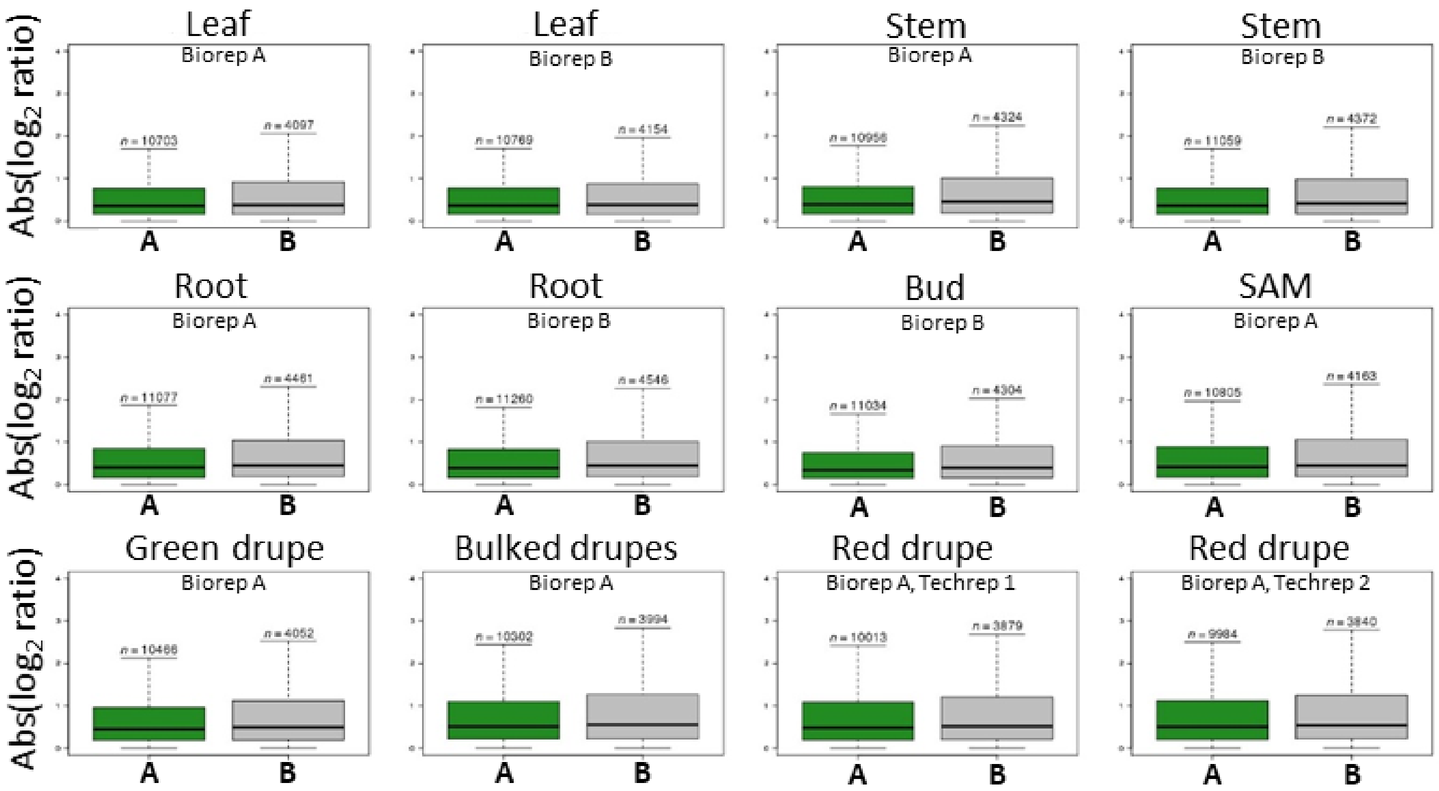

Supplementary Fig. 27. Differences in gene expression levels between homoeologous genes located in A or B chromatin compartments in different organs. Green box plots show distributions of absolute values of Transcripts Per Million (TPM) log2 ratios for homoeologous gene pairs located in A chromatin compartments. Gray box plots show distributions of absolute values of Transcripts Per Million (TPM) log_2 ratios for homoeologous gene pairs located in B chromatin compartments. The number of genes with TPM>1 is indicated above each plot (*n*=). Box plot distributions showed statistically significant differences between compartments using a two-sided Wilcoxon test except for red drupes Biorep A Techrep 1 and young leaves Biorep B. BioRep A and B stand for Biological Replicates (i.e. independent RNA extraction from two plants). Techrep 1 and 2 stand for Technical Replicates (i.e. independent RNA extraction from specimens sampled from the same plant). SAM stands for Shoot Apical Meristem. Bulked drupes stand for multiple drupes at different developmental stages sampled on the same plant. Boxes indicate the first and third quartiles, the horizontal line within the boxes indicates the median and the whiskers indicate ±1.5 × interquartile range. Source data are provided as a Source Data file.

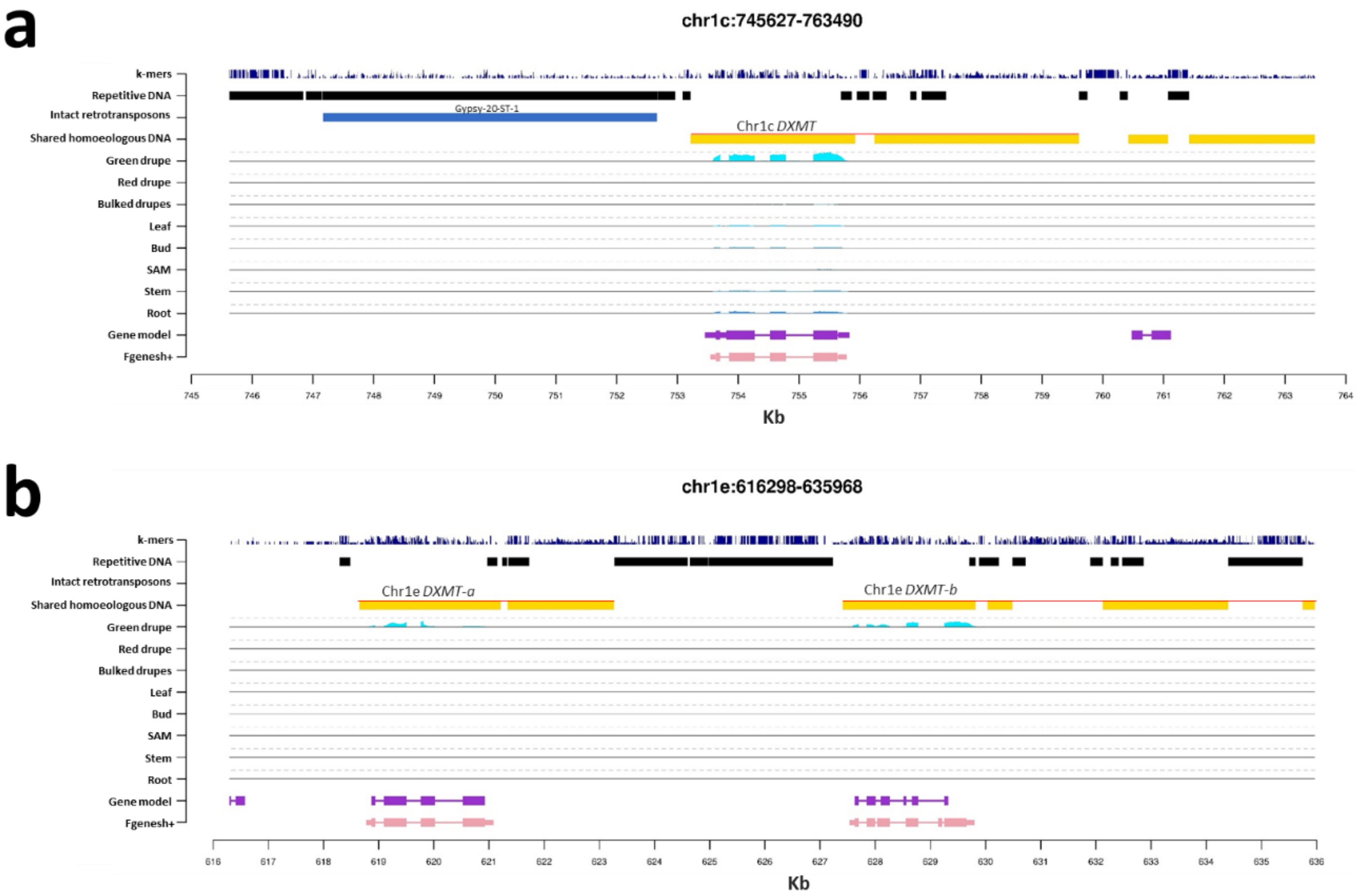

Supplementary Fig. 28. Graphical representation of the *DXMT* gene in the *C. arabica* Chr1c and Chr1e homoeologs. a Genomic features in the canephora homoeolog. **b** Genomic features in the eugenioides homoeolog. The navy blue track shows k-mer abundance. The black track shows repetitive DNA that was masked by Repeat Masker using the database of intact TEs generated by

EDTA. The royal blue track shows retrotransposons with intact target site duplications, named after the most similar element found in Repbase. The gold track shows shared collinear regions between homoeologs. The red line above shared collinear regions identifies the region in the canephora homoeolog (**a**) that underwent a segmental tandem duplication in the eugenioides homoeolog (**b**). The cyan tracks show gene expression profiles in different organs. The y-axis of each cyan track shows normalized RNA-Seq read coverage (reads counts per nucleotide position × 10^6 divided by the total number of mapped reads). The y-axis maximum value is set to 190 in all cyan tracks in **a** and **b**. Normalized levels of gene expression (Transcripts Per Million, TPM) are given in Supplementary Fig. 29. Purple and pink tracks show, respectively, predicted gene models that were obtained from automated gene prediction and curated gene models that were obtained using Fgenesh+ with full-length protein support from functionally characterized plant DXMT proteins. X-axes indicate kilobase pairs (Kb).

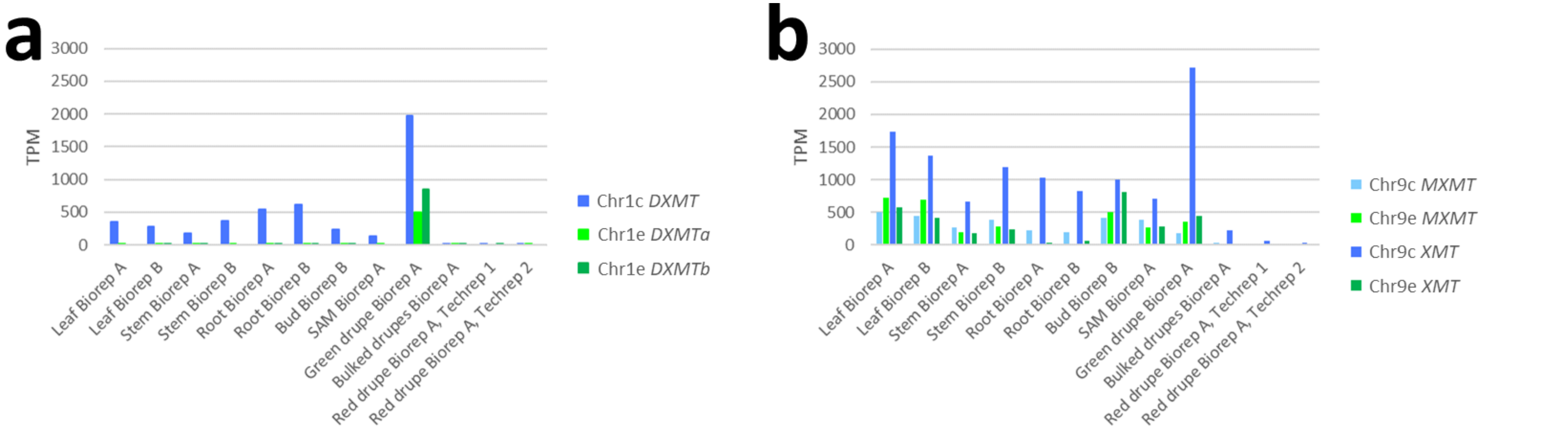

Supplementary Fig. 29. Gene expression of *C. arabica DXMT*, *MXMT* and *XMT* homoeologous copies of the caffeine biosynthetic pathway in different organs. a *DXMT* homoeologs in Chr1c (navy blue) and Chr1e (two paralogs, light and forest green). **b** *MXMT* and *XMT* homoeologs in the Chr9c (steel blue and navy blue) and Chr9e (light and forest green) gene clusters. BioRep A and B stand for Biological Replicates (i.e. independent RNA extraction from two plants). Each multi mapping RNA read contributed 1/n to the transcript coverage, where n stands for the number of matching transcripts. Techrep 1 and 2 stand for Technical Replicates (i.e. independent RNA extraction from specimens sampled from the same plant). SAM stands for Shoot Apical Meristem. Bulked drupes stand for multiple drupes at different ripening stages sampled on the same plant. Y-axes indicate Transcripts per Million (TPM). Source data are provided as a Source Data file.

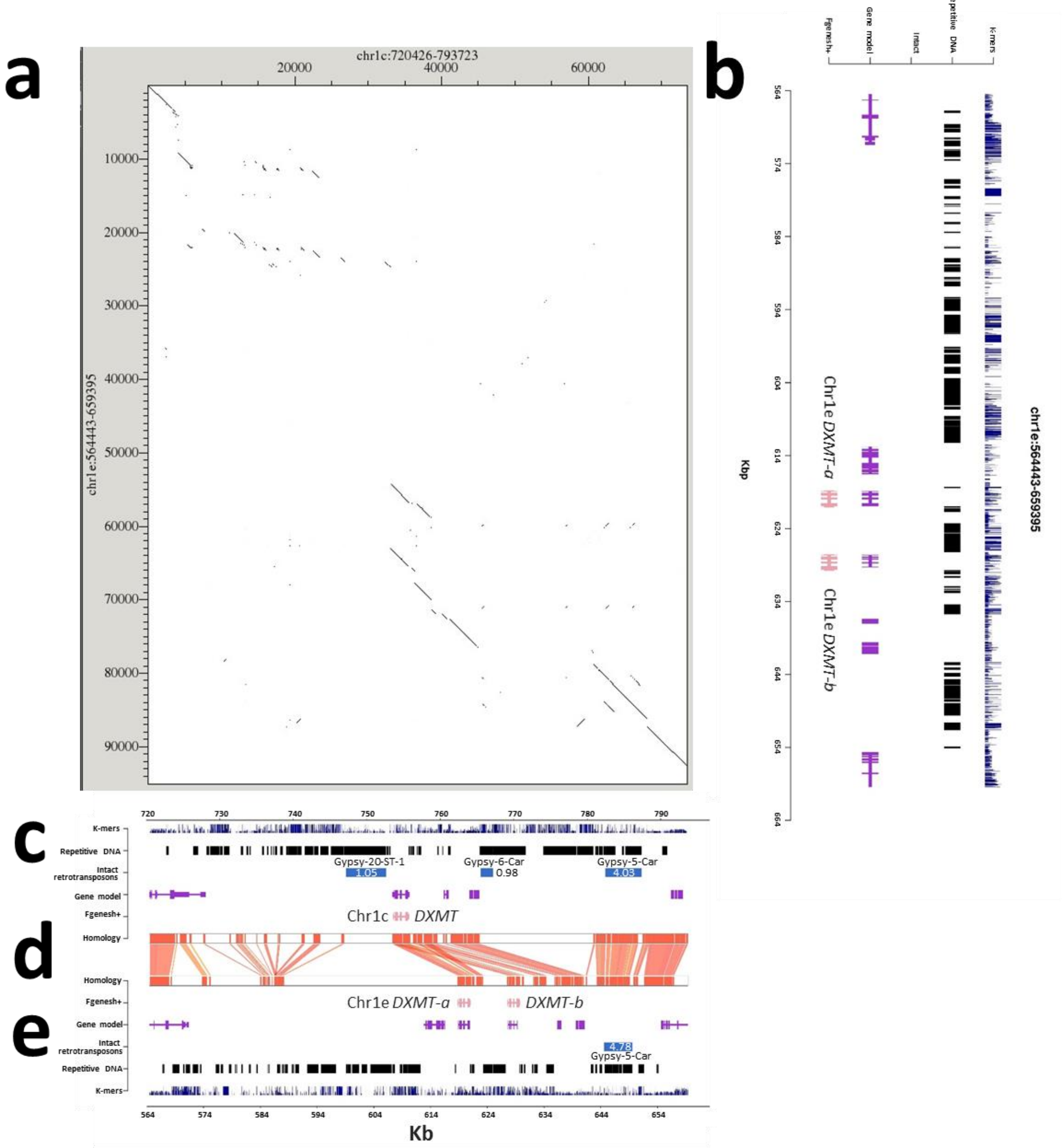

Supplementary Fig. 30. Homoeologous structural variation in *C. arabica* across the *DXMT* locus. a Dot plot comparison between canephora (horizontal) and eugenioides (vertical) homoeologs. **b**,**e** Genomic features in the eugenioides homoeolog. **c** Genomic features in the canephora homoeolog. The navy blue track in **b**, **c** and **e** shows k-mer abundance. The black track in **b**, **c** and **e** shows repetitive DNA that was masked by Repeat Masker using the database of intact TE generated by EDTA. The royal blue track in **b**, **c** and **e** shows retrotransposons with intact target site duplications, named after the most similar element found in Repbase. Number

indicated the estimated time of insertion (million years) based on LTR sequence divergence. Purple and pink tracks in **b**, **c** and **e** show, respectively, predicted gene models obtained from automated gene prediction and curated gene models obtain using Fgenesh+ with full-length protein support from functionally characterized plant DXMT proteins. **d** Red bars and connectors show shared collinear regions between homoeologs. X-axes indicate kilobase pairs (Kb).

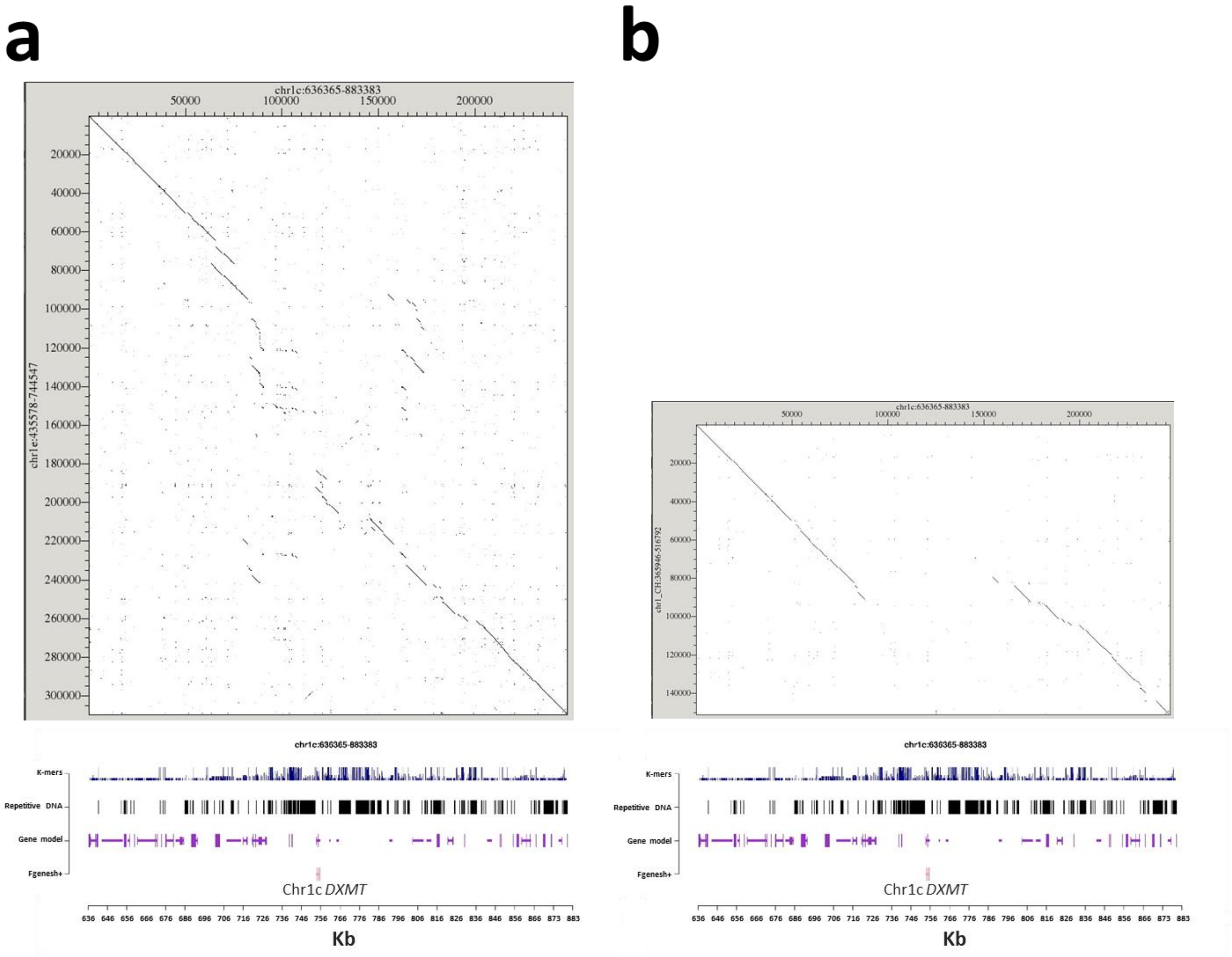

Supplementary Fig. 31. Synteny among homoeologs across a wider region in which the *DXMT* locus is located. a Dot plot comparison between canephora-derived (horizontal) and eugenioides-derived (vertical) homoeologs in *C. arabica*. **b** Dot plot comparison between the canephora-derived (horizontal) homoeolog in *C. arabica* and the *C. humblotiana* (vertical) homoeolog. The navy blue track shows k-mer abundance. The black track shows repetitive DNA that was masked by Repeat Masker using the database of intact TEs generated by EDTA. The purple track shows predicted gene models obtained from automated gene prediction. The pink track shows the location of the *DXMT* gene in *C. arabica* Chr1c. X-axes indicate kilobase pairs (Kb).

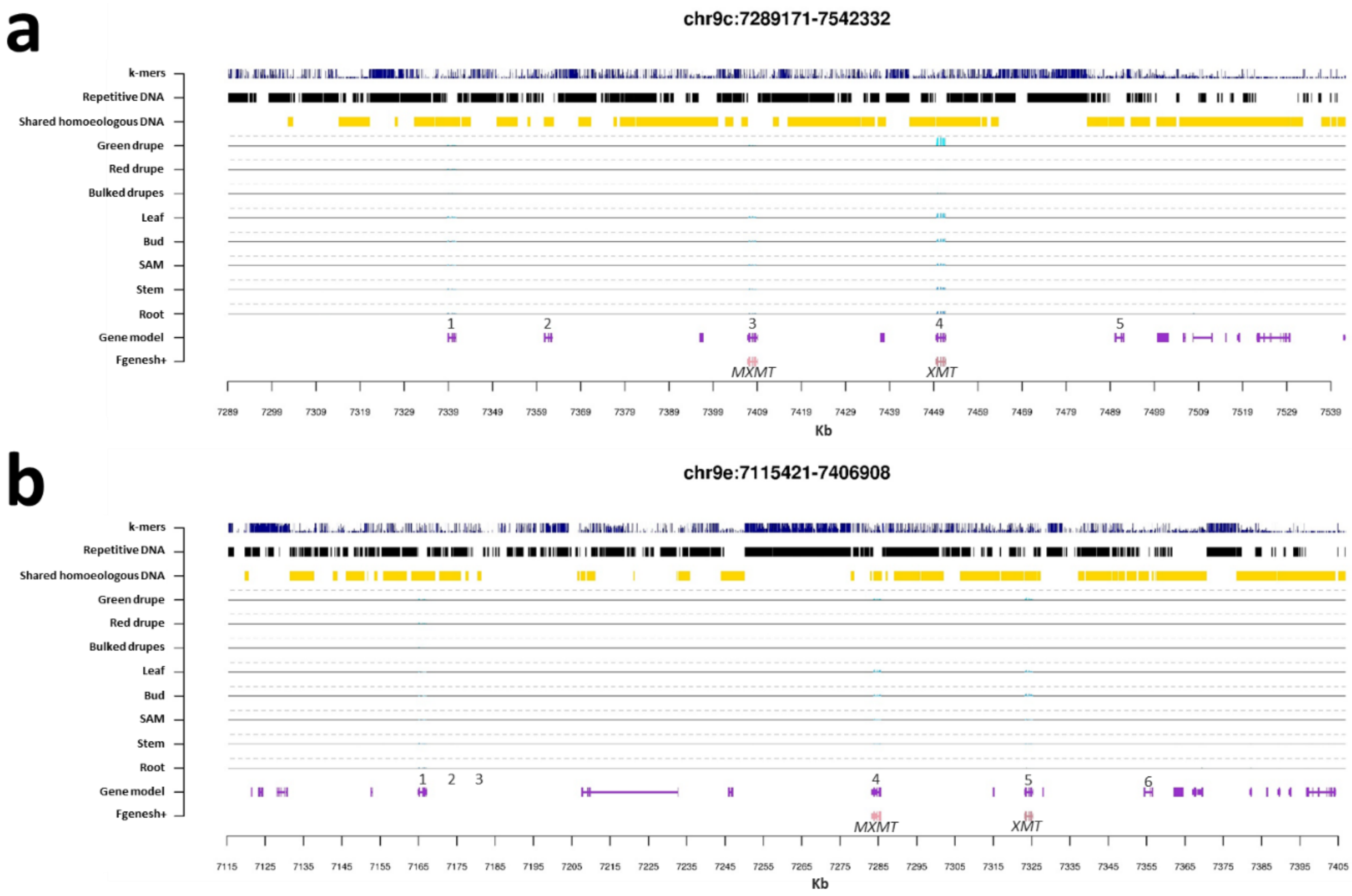

Supplementary Fig. 32. Graphical representation of the *MXMT* and *XMT* gene cluster in *C. arabica* Chr9c and Chr9e homoeologs. a Genomic features in the canephora homoeolog. **b** Genomic features in the eugenioides homoeolog. The navy blue track shows k-mer abundance. The black track shows repetitive DNA that was masked by Repeat Masker using the database of intact

TE generated by EDTA. The gold track shows shared collinear regions between homoeologs. The cyan tracks show gene expression profiles in different organs. The y-axis of each cyan track shows normalized RNA-Seq read coverage (reads counts per nucleotide position × 10^6 divided by the total number of mapped reads). The y-axis maximum value is set to 270 in all cyan tracks in **a** and **b**. Normalized levels of gene expression (Transcripts Per Million, TPM) are given in Supplementary Fig. 29. The purple track shows predicted gene models obtained from automated gene prediction. The numbers above the purple track identify regions showing similarity of the translated nucleotide sequence with the *C. canephora* predicted proteins Cc09_g06970 and Cc00_g24720. The pink track shows the location of the *MXMT* and *XMT* genes in *C. arabica* corresponding to the *C. canephora* predicted proteins Cc00_g24720 and Cc09_g06970. X-axes indicate kilobase pairs (Kb).

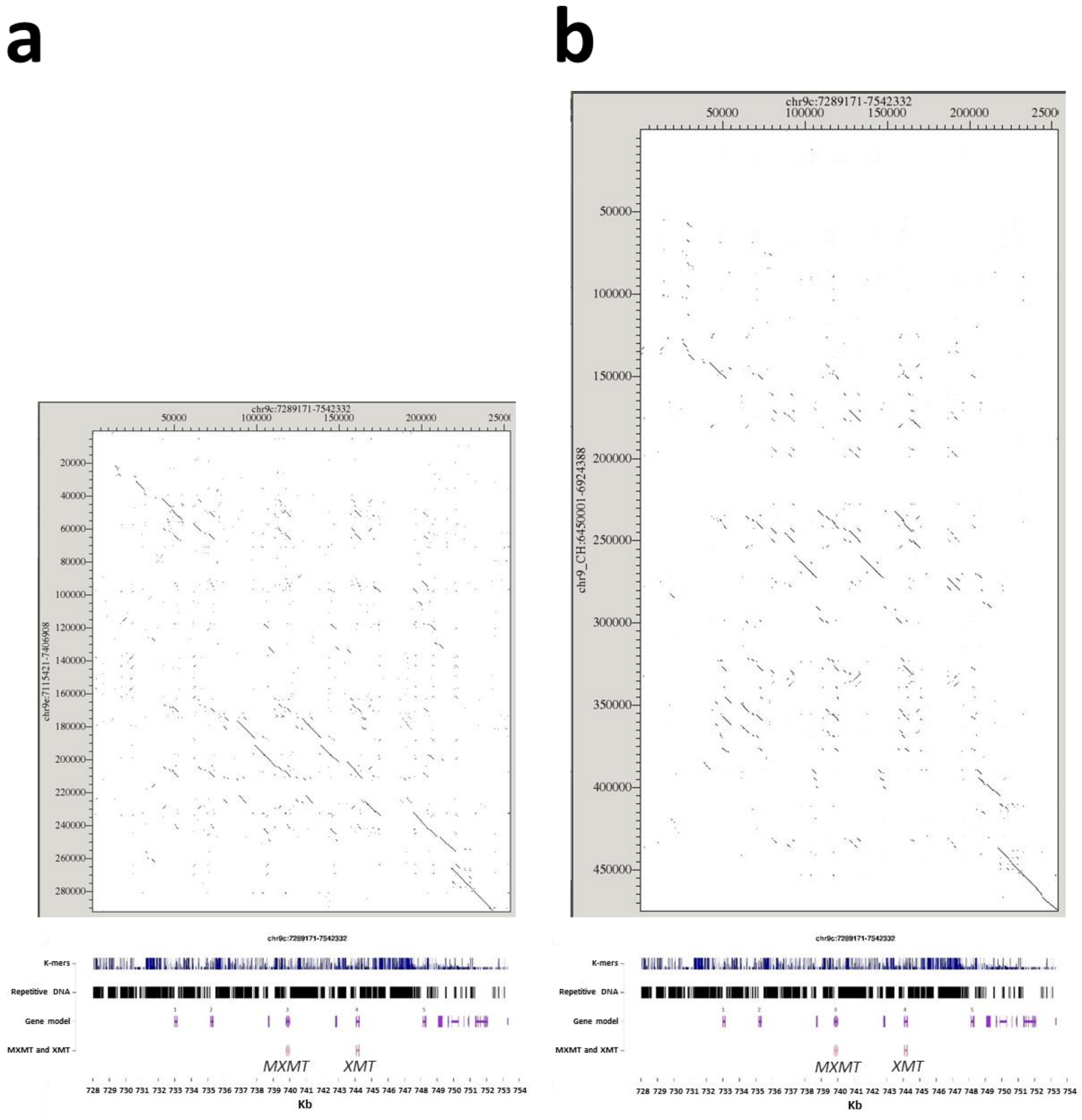

Supplementary Fig. 33. Synteny among homoeologs across a wider region in which the *MXMT* and *XMT* genes are located. a Dot plot comparison between canephora-derived (horizontal) and eugenioides-derived (vertical) homoeologs in *C. arabica*. **b** Dot plot comparison between the canephora-derived (horizontal) homoeolog in *C. arabica* and the *C. humblotiana* (vertical) homoeolog. The navy blue track shows k-mer abundance. The black track shows repetitive DNA that was masked by Repeat Masker using the database of intact TEs generated by EDTA. The purple track shows predicted gene models obtained from automated gene prediction. The pink track shows the location of the *MXMT* and *XMT* genes in *C. arabica* corresponding to the *C. canephora* predicted proteins Cc00_g24720 and Cc09_g06970. X-axes indicate kilobase pairs (Kb).

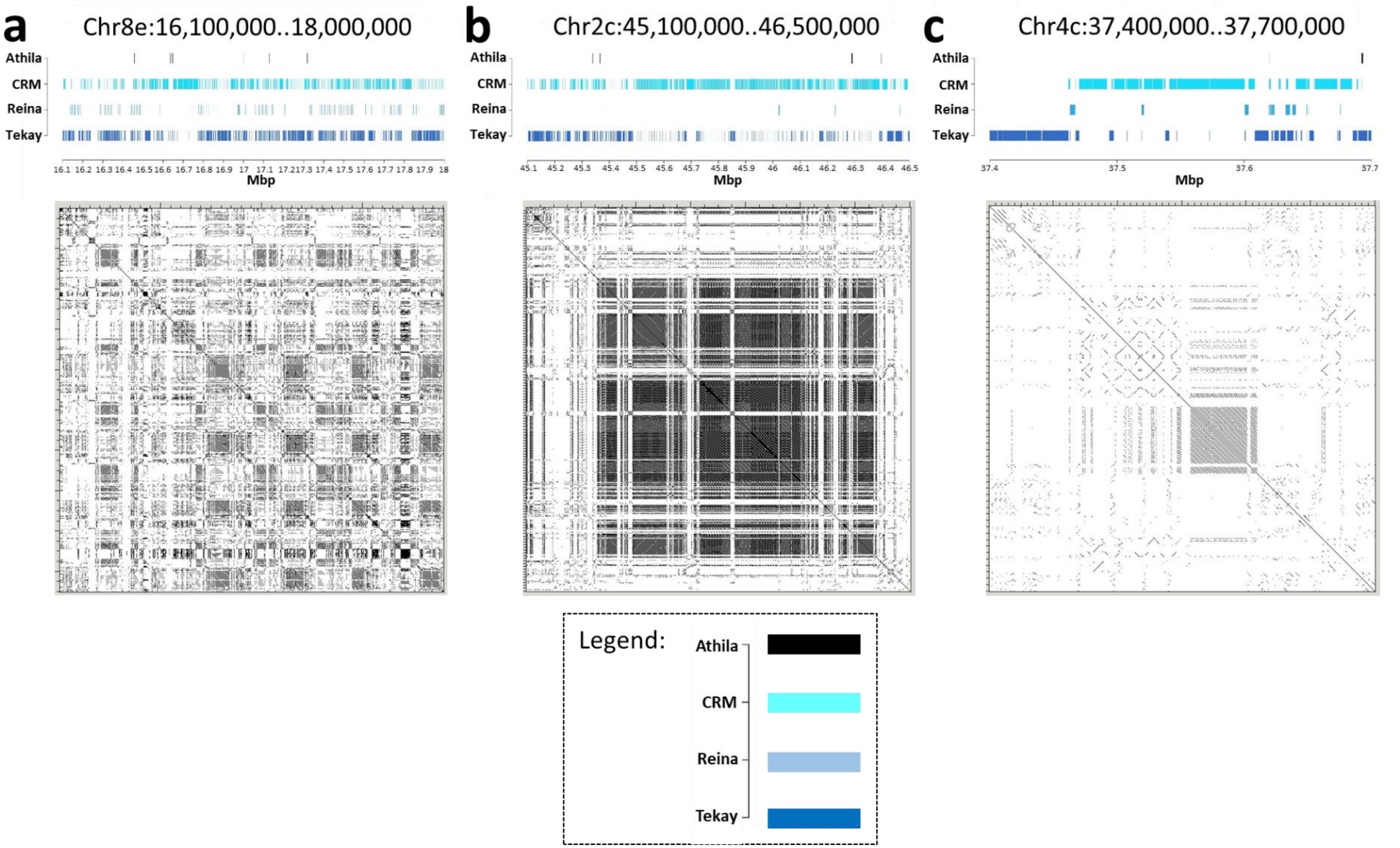

Supplementary Fig. 34. Self dot plot of three representative chromosomal regions representing different levels of structural complexity and sizes in chromovirus-derived and/or Athila-derived satellite arrays. a Multiple arrays of diverse and interspersed chromovirus-derived monomers. **b** Intermixed arrays of a conserved CRM-derived monomer and a conserved Tekay-derived monomer. **c** Mixture of arrays of diverse chromovirus-derived monomers and arrays of Athila-derived monomers. The colored bars represent the intervals masked by Repeat Masker using Athila, CRM, Reina and Tekay intact TE sequences. X-axes indicate million base pairs (Mbp).

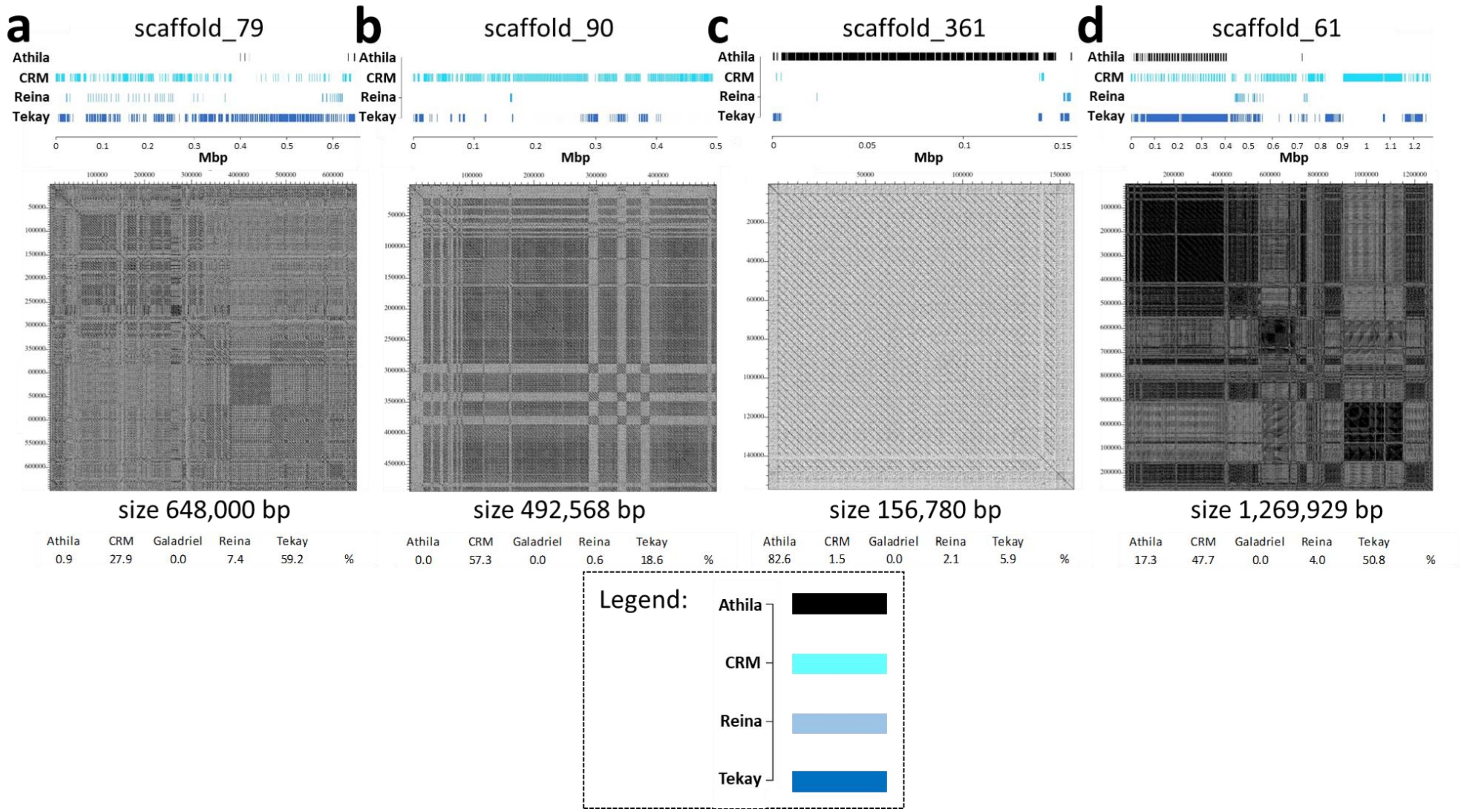

Supplementary Fig. 35. Self dot plot of four representative unanchored scaffolds representing different levels of structural complexity in chromovirus-derived and/or Athila-derived satellite arrays. a Multiple arrays of diverse and interspersed chromovirus-derived monomers. **b** Intermixed arrays of a conserved CRM-derived monomer and a conserved Tekay-derived monomer. **c** Array of a single conserved Athila-derived monomer. **d** Mixture of arrays of diverse chromovirus-derived monomers and arrays of Athila-derived monomers. The colored bars represent the intervals masked by Repeat Masker using Athila, CRM, Reina and Tekay intact TE sequences. X-axes indicate million base pairs (Mbp).

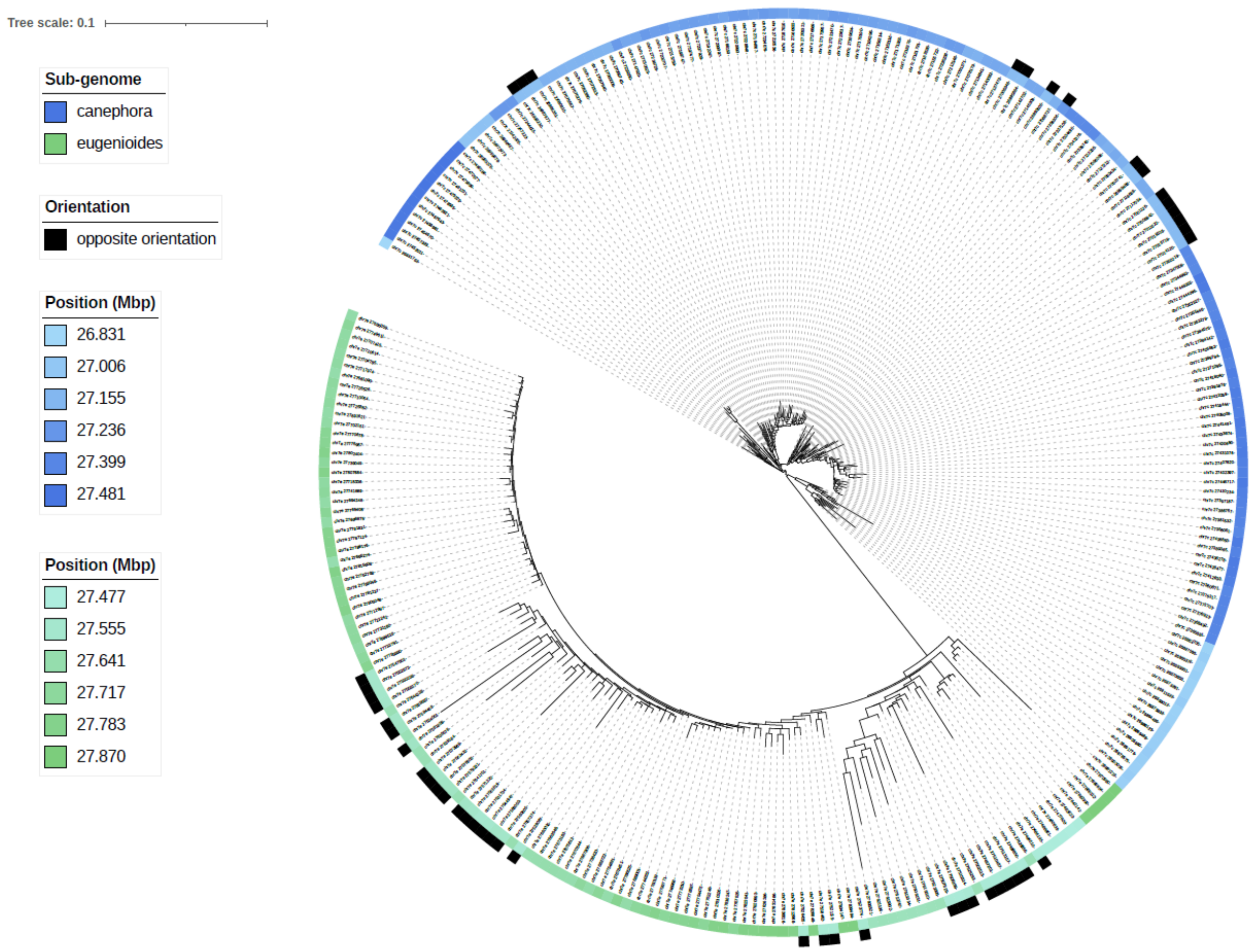

Supplementary Fig. 36. Phylogeny of the 2,683-bp CRM-derived monomer that has generated the tandem repeated array in Chr7c and Chr7e illustrated in Fig. 2b. Heatmaps indicate the relative position of each monomer within the satellite array in Chr7c (blue heatmap) and in Chr7e (green heatmap). Position is expressed as chromosomal coordinates in million base pairs (Mbp). The outer black track marks monomers that are present in opposite orientation with respect to the predominant orientation in the array. The phylogenetic tree was constructed using IQ-TREE[11] and plotted with iTOL[12].

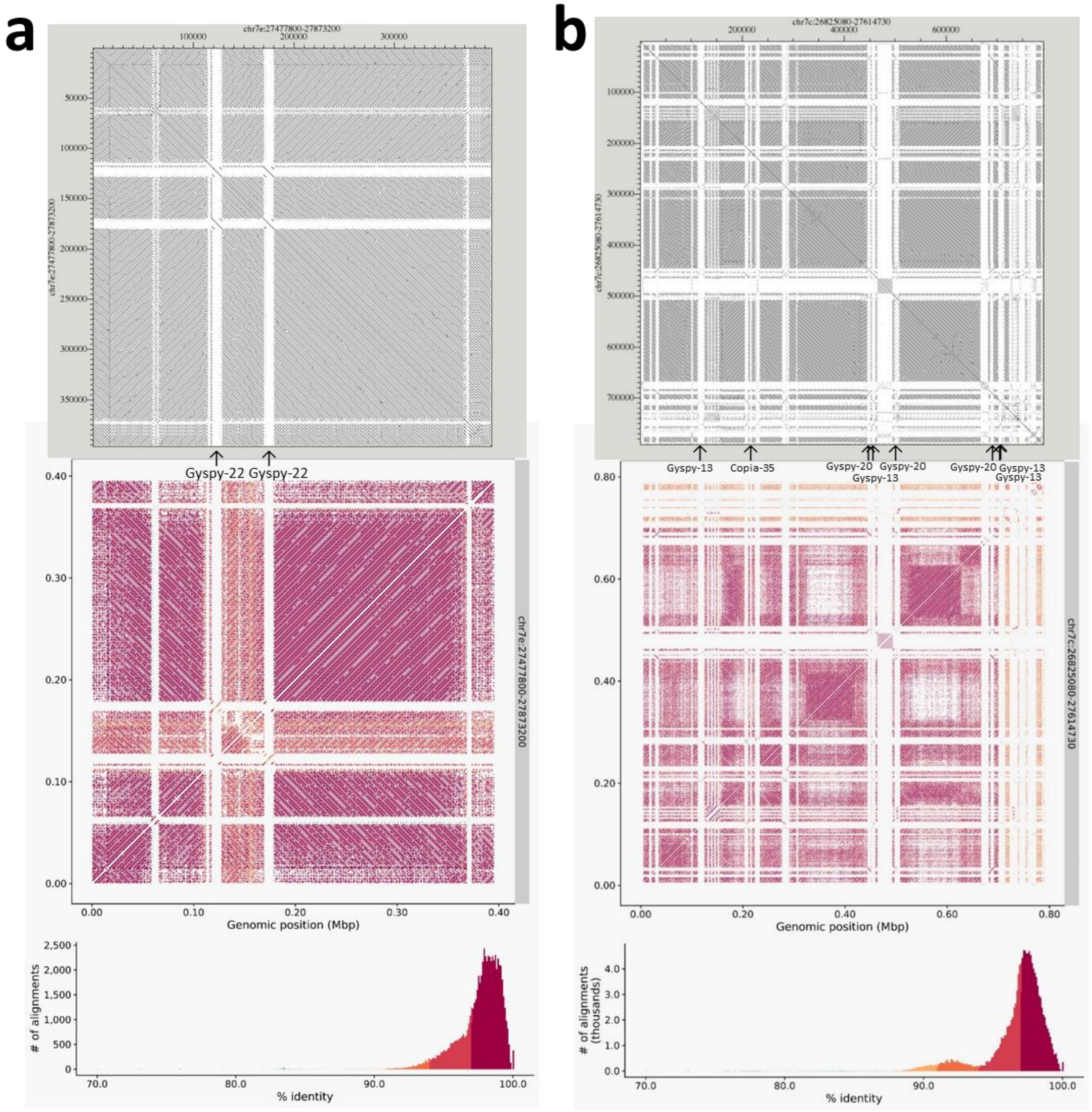

Supplementary Fig. 37. Structural organization of the tandem repeat array generated by the 2,683-bp CRM-derived monomer. a Chr7c. **b** Chr7e. In each panel, the upper graph in gray scale shows a self dot plot comparison of the nucleotide sequence of the array. The dot plot was generated using dotter. The lower graph in spectral colour brewer palette shows the identity plot among 1 Kb windows obtained using StainedGlass. The histogram at the bottom shows the levels of identity between pairs of the 1 Kb windows and the corresponding frequency with which they are found using the same colour key as above. Arrows point to the position of LTR-retrotransposon insertions. LTR-retrotransposons are named after the most similar matched element in Repbase.

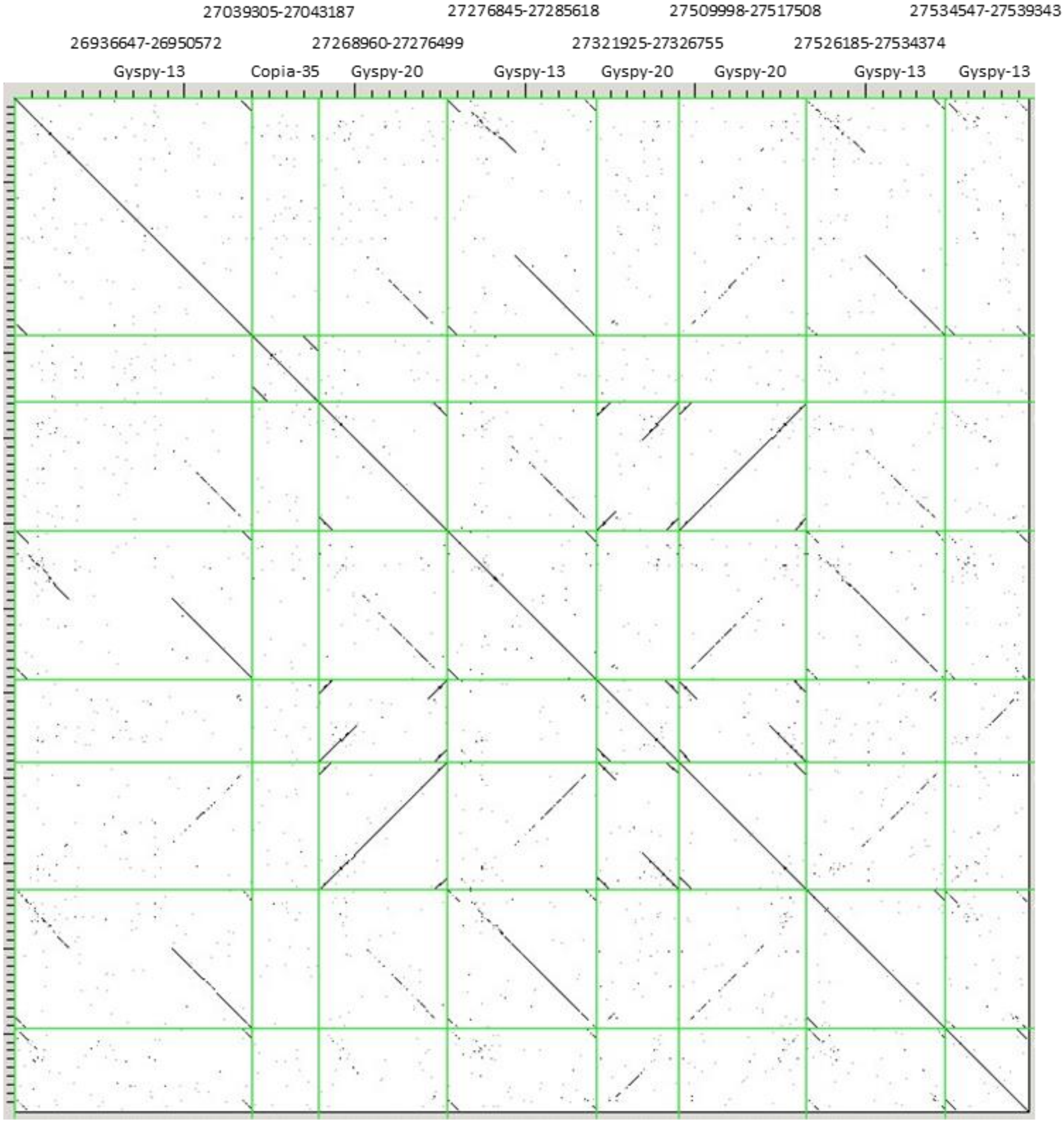

Supplementary Fig. 38. Dot plot comparisons of the LTR-retrotransposon insertions into the tandem repeat array on Chr7c generated by the 2,683-bp CRM-derived monomer shown in Supplementary Fig. 37. The all-versus-all comparison of the nucleotide sequence of the concatenated elements was generated using dotter. Green lines define the boundaries of each element. Details of each LTR-retrotransposon are reported in tabular format in Supplementary Table 7. The numbers above each element indicate the position of the element in Chr7c, expressed as chromosomal coordinates.

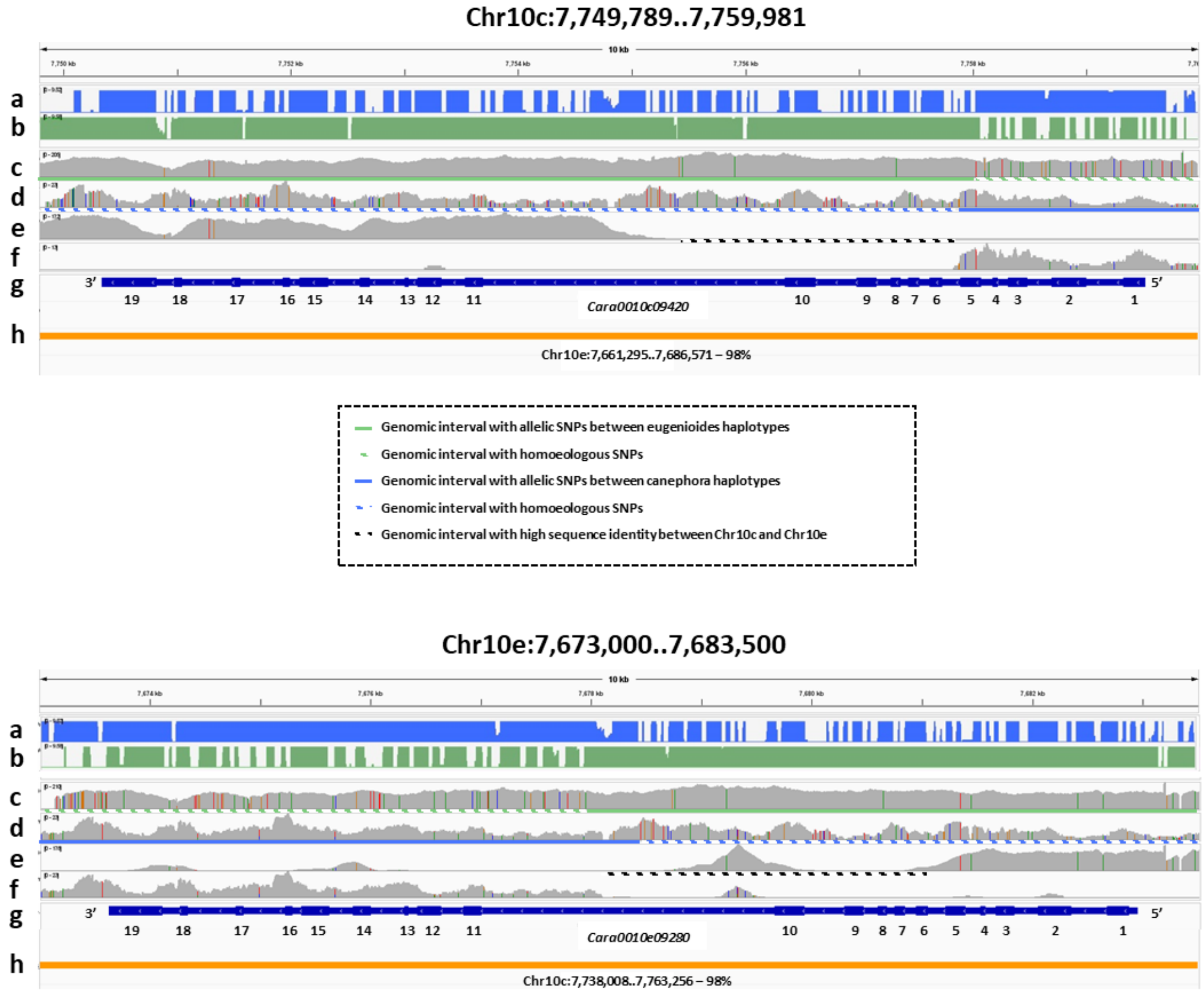

Supplementary Fig. 39. Details of the intragenic homoeologous exchange between chromosomes Chr10c and Chr10e. a 51-mers canephora plot. **b** 51-mers eugenioides plot. **c** *C. eugenioides* Illumina read coverage and allelic and homoeologous SNPs, obtained from read alignments against the canephora subgenome of *C. arabica* (chromosome pseudomolecules from Chr1c to Chr11c). **d** *C. canephora* Illumina read coverage and allelic and homoeologous SNPs obtained from read alignments against the eugenioides subgenome of *C. arabica* (chromosome pseudomolecules from Chr1e to Chr11e). **e** *C. eugenioides* Illumina read coverage and allelic SNPs obtained from read alignments against the *C. arabica* genome. **f** *C. canephora* Illumina read coverage and allelic SNPs obtained from read alignments against the *C. arabica* genome. **g** Gene model predictions. Number indicate exons. **h** Homoeologous intervals in *C. arabica*.

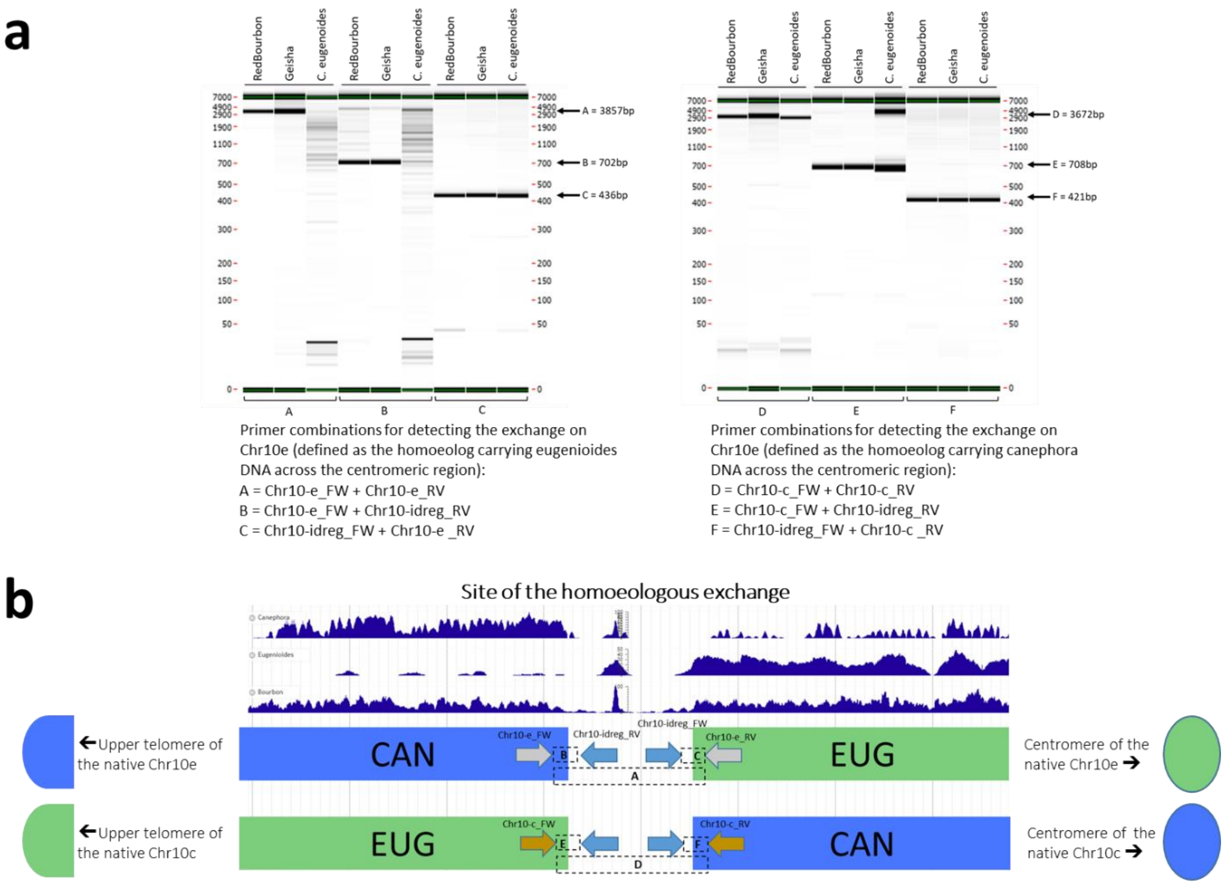

Supplementary Fig. 40. PCR-based validation of the homoeologous exchange between chromosomes Chr10c and Chr10e. a Sizing of the amplicons obtained from ‘Bourbon’, ‘Geisha’ and *C. eugenionides* DNA using the primer combinations A through F reported in **a**. **b** Scheme of the site of the homoeologous recombination, primer annealing sites (arrows) and expected amplicons (dashed line boxes, identified with A through F letters. The expected length of each amplicon based on the Bourbon reference sequence is reported in **a** flanking each actual amplicon. Blue plots in **b** show the coverage of uniquely mapping *C. canephora* reads (upper plot), *C. eugenioides* reads (middle plot) and Bourbon reads (lower plot) on the Bourbon reference sequence. The exact site of recombination is flanked by a tract of identical DNA sequence on both Chr10c and Chr10e (represented by a white background in **b**), which prevented sequencing reads to be uniquely mapped and was used to design primers that align on both chromosomes. Blast alignments of this tract against the diploid progenitor species indicated that it is more similar to *C. eugenioides* than to *C. canephora*. Primer sequences are given in Supplementary Table 11.

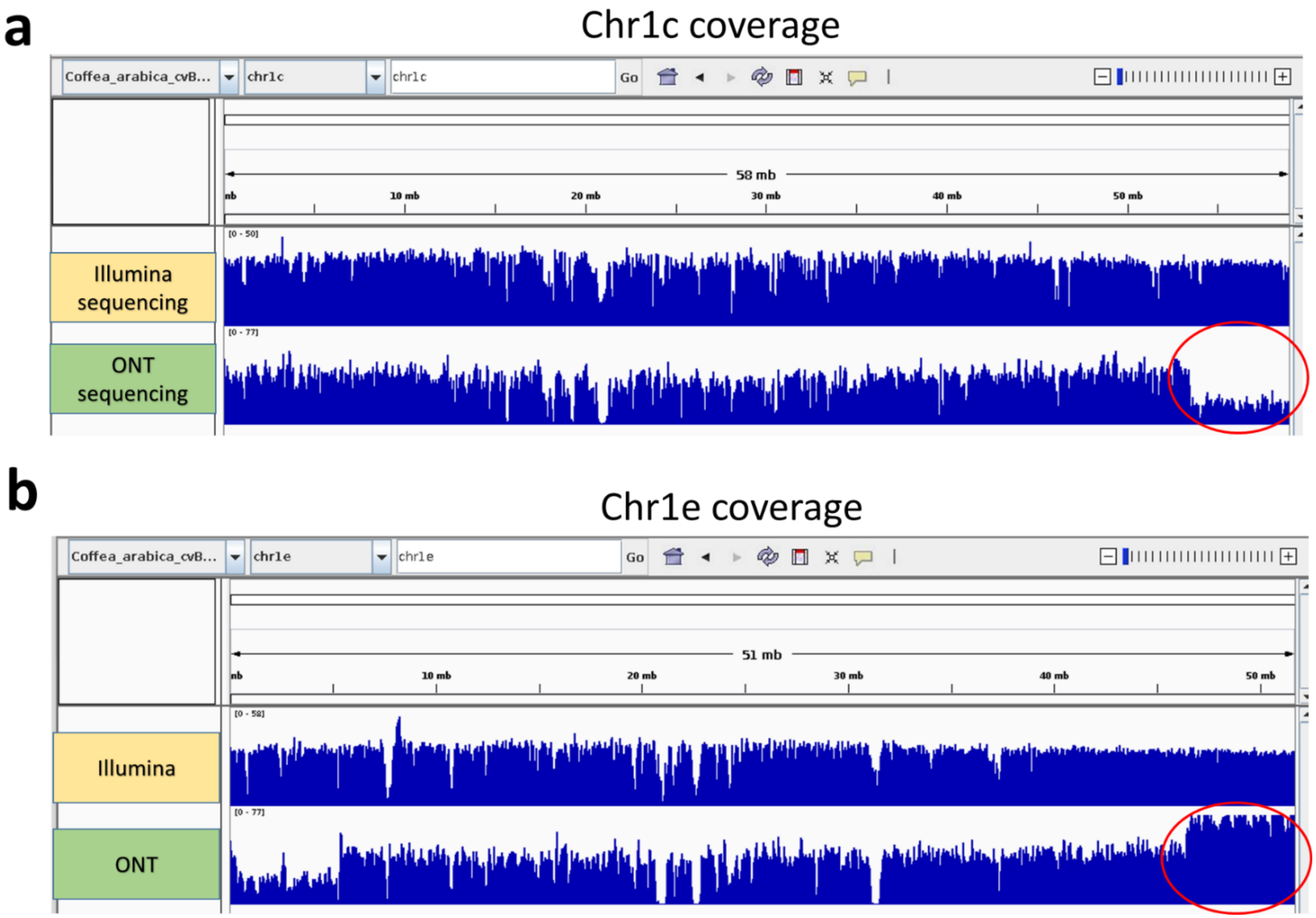

Supplementary Fig. 41. Read coverage plot along the Chr1 pseudomolecules of the Bourbon genome assembly. a Chr1c. **b** Chr1e. Illumina and ONT reads were obtained from DNA extractions and whole-genome sequencing of two different Bourbon specimens. Illumina sequencing has generated an average genome coverage of 30X. ONT sequencing has generated an average genome coverage of 50X. The y-axis (coverage) of the Illumina-derived plot is set 0-50X in **a** and **b**. The y-axis (coverage) of the ONT-derived plot is set 0-77X in **a** and **b**. The red oval indicates the chromosome segments with opposite deviation from normal read coverage in the two homoeologs.

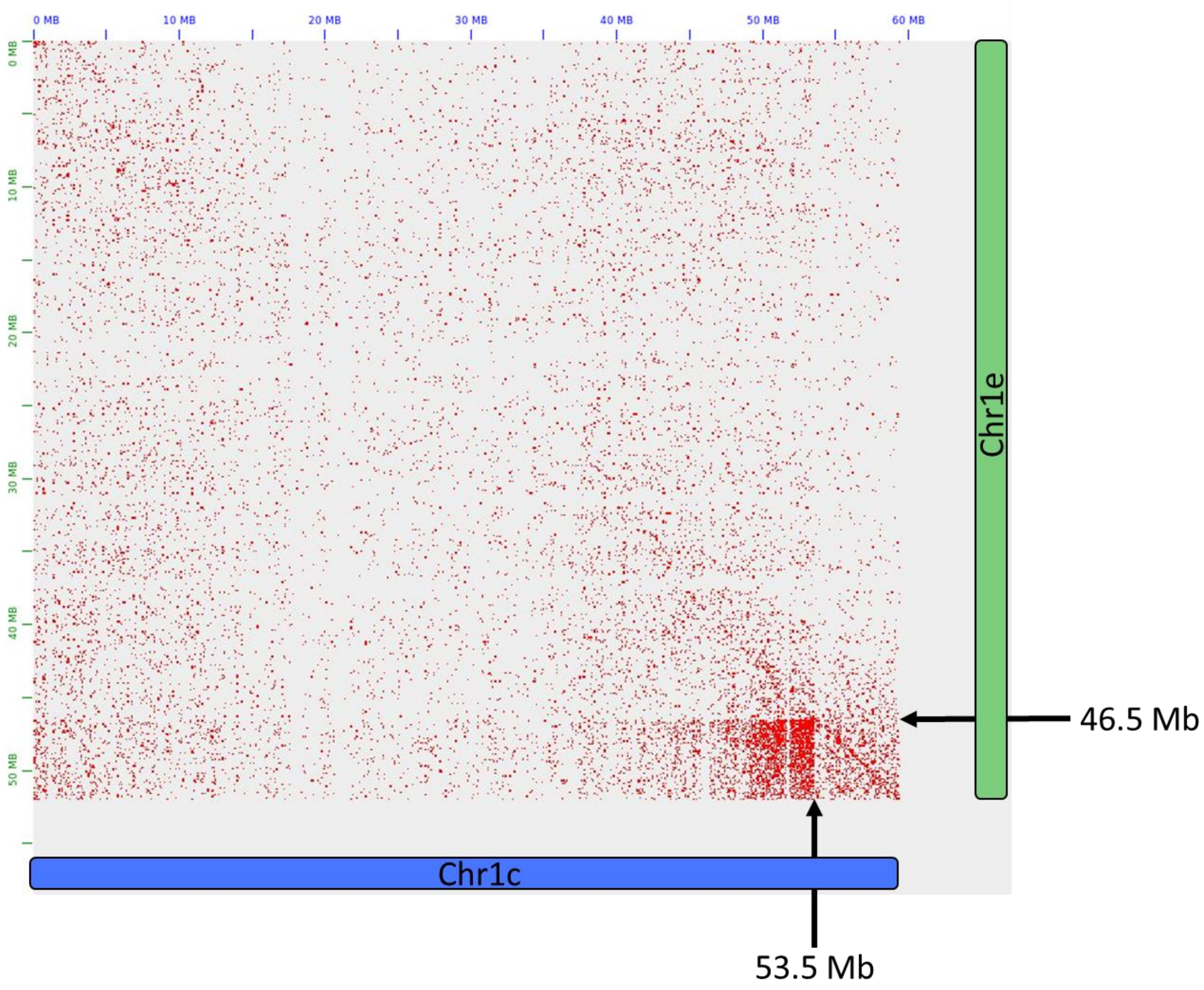

Supplementary Fig. 42. Hi-C contact map between Chr1c and Chr1e homoeologs. The arrows point to the position of the homoeologous replacement shown in Supplementary Fig. 41.

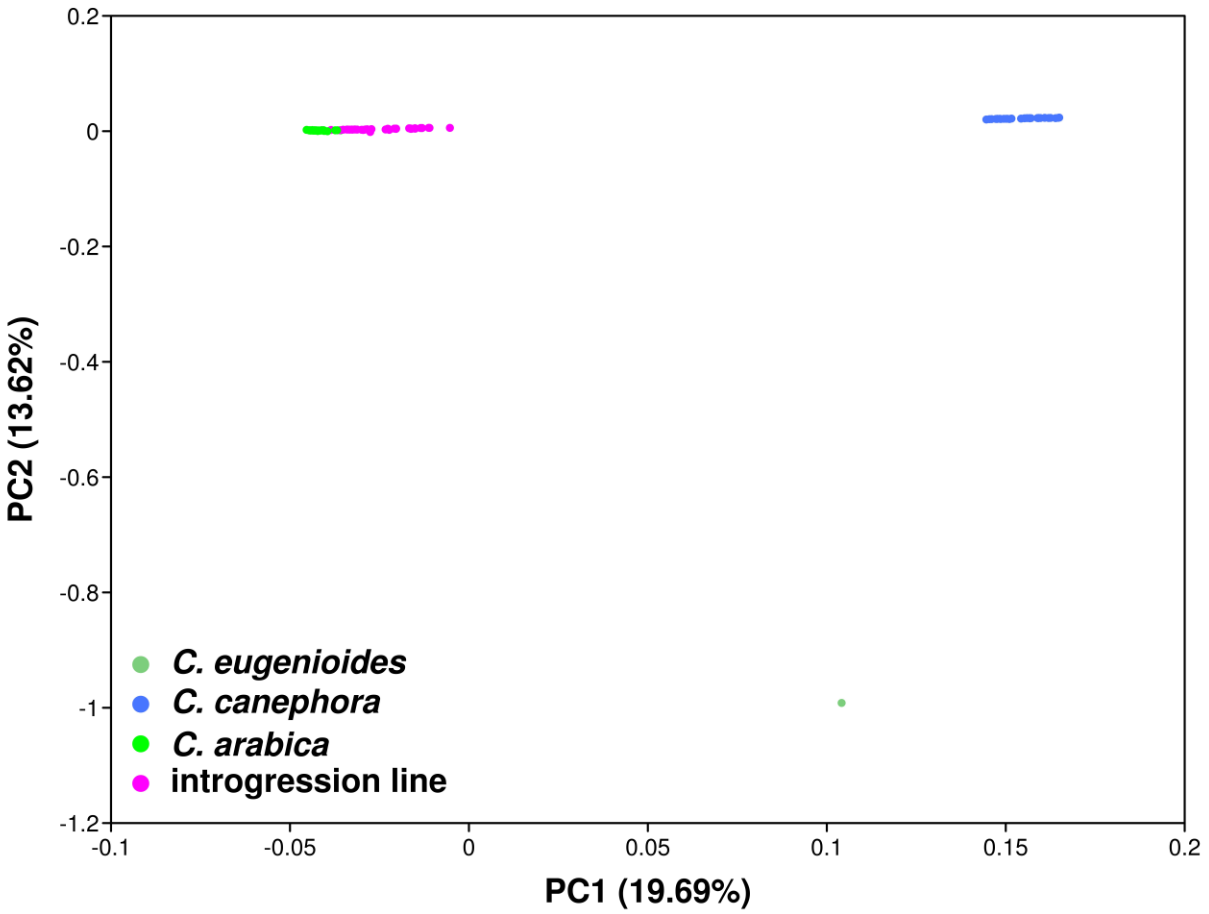

Supplementary Fig. 43. Principal Component Analysis (PCA) of the set of 173 *Coffea* sp. accessions, including the accession of *C. eugenioides* that was removed from Fig. 3a. Axes indicate the first two principal components (PC). It has to be noted that the *C. canephora* accession 33-1 has been removed from the PCA due to its low coverage (Supplementary Data 1). Source data are provided as a Source Data file.

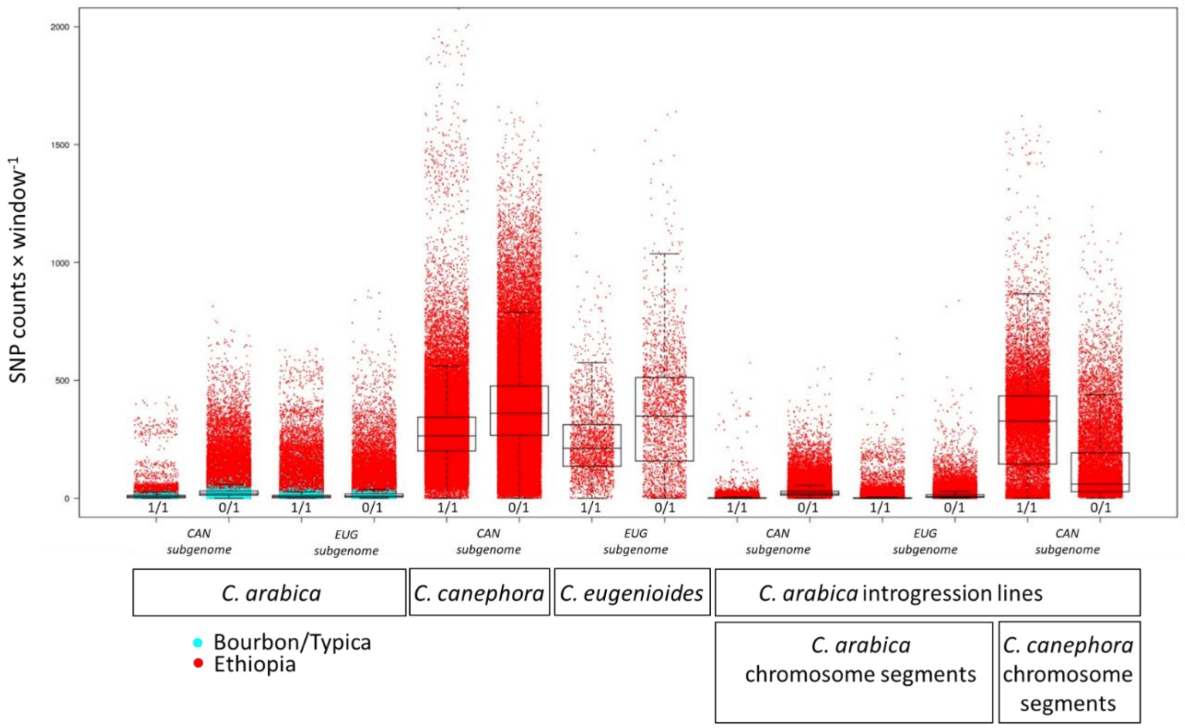

Supplementary Fig. 44. Box plot distribution of SNP counts across 4,467 non-overlapping genomic windows containing 100 Kb of non-repetitive DNA in 174 *Coffea* sp. accessions sorted by their taxonomic assignment. Within each taxa, SNP counts are sorted by their subgenome location with respect to the Bourbon reference genome (CAN stand for canephora, EUG stands for eugenioides) and by their zygosity state (1/1 homozygous alternative with respect to the Bourbon reference allele, 0/1 heterozygous with respect to the Bourbon reference allele). SNP counts in *C. arabica* × *C. canephora* introgression lines are further sorted by either residing in consecutive genomic windows with signatures of *C. canephora* introgression that make up chromosomes segments of *C. canephora* introgression or residing in the rest of genomic windows that are assumed to represent the *C. arabica* genetic background. Boxes indicate the first and third quartiles, the horizontal line within the boxes indicates the median and the whiskers indicate ±1.5 × interquartile range. Source data are provided as a Source Data file.

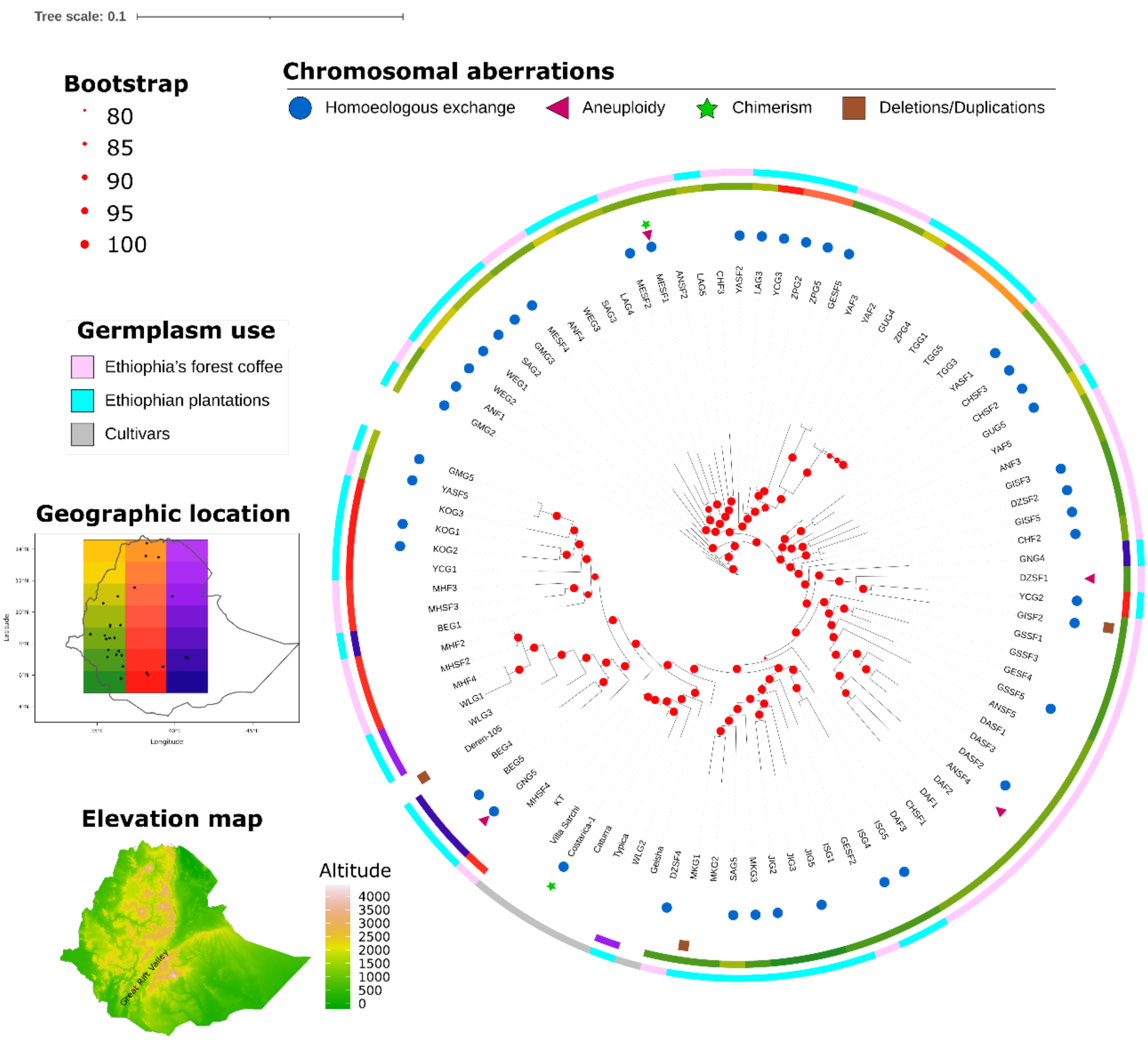

Supplementary Fig. 45. Phylogenetic tree of *C. arabica* based on 1,877,440 SNPs. The colours in the outer circle represent different types of germplasm, based on their use as defined by Mekbib and colleagues[19] (Supplementary Data 1). The colours in the inner circle represent different geographic areas in Ethiopia where the accessions were collected. The geographic location of sampling sites in Ethiopia is indicated by black dots on the colour key (the borders of the country are indicated by the black line). The symbols between the circles mentioned above and accession names indicate the presence of one or more chromosomal aberration and/or exchange in the corresponding accession as described in Supplementary Figs. 57-60. Branch length is proportional to the tree scale indicated in the legend. The Ethiopian elevation map was drawn using GADM data v4.1 (https://gadm.org/).

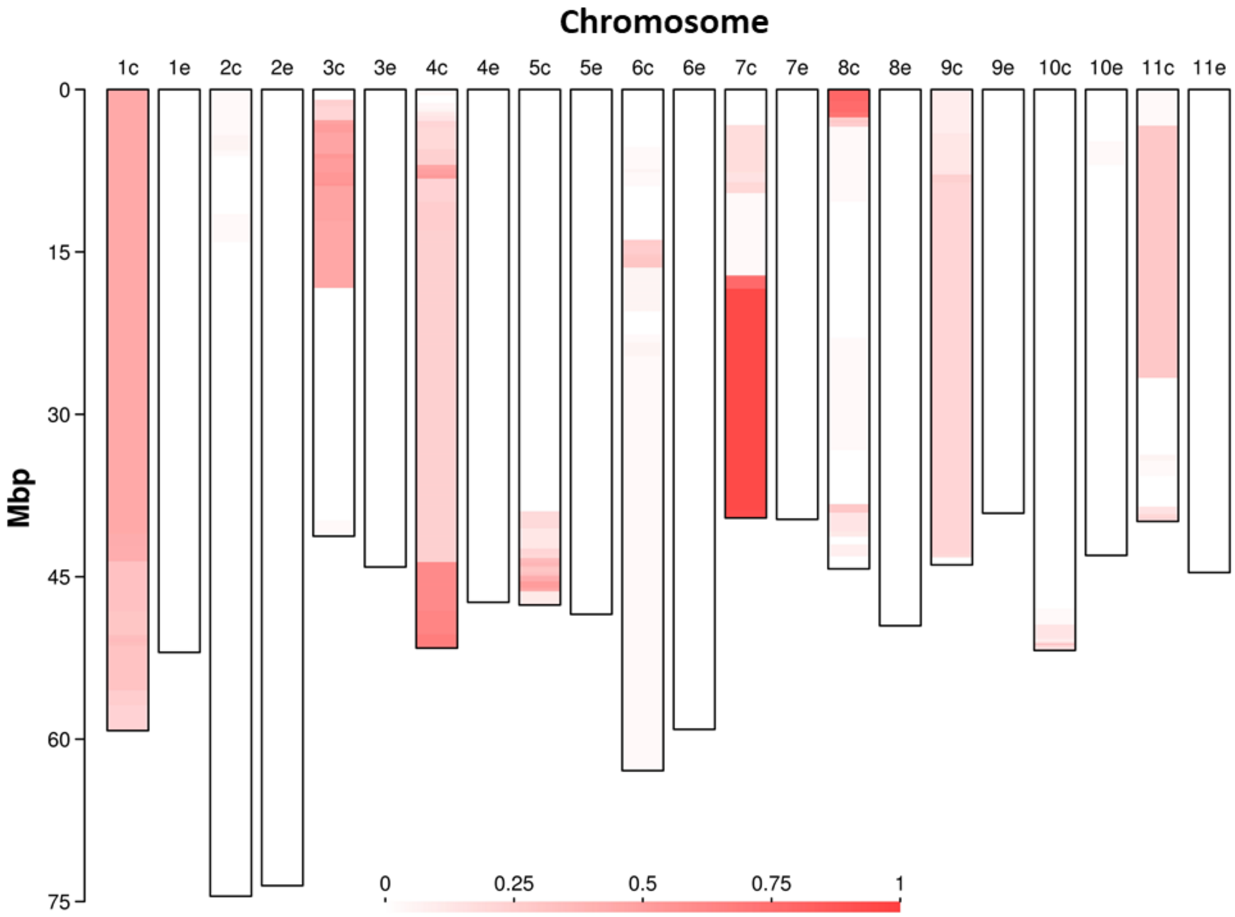

Supplementary Fig. 46. Introgressed haplotype frequency across 4,467 non-overlapping genomic windows containing 100 Kb of non-repetitive DNA in a set of 37 canephora introgression lines (Timor hybrid derivatives). Ideograms represent chromosomes. The red-to-white heatmap indicates introgressed haplotype frequency. The y-axis indicates chromosome length in million base pairs (Mbp). Source data are provided as a Source Data file.

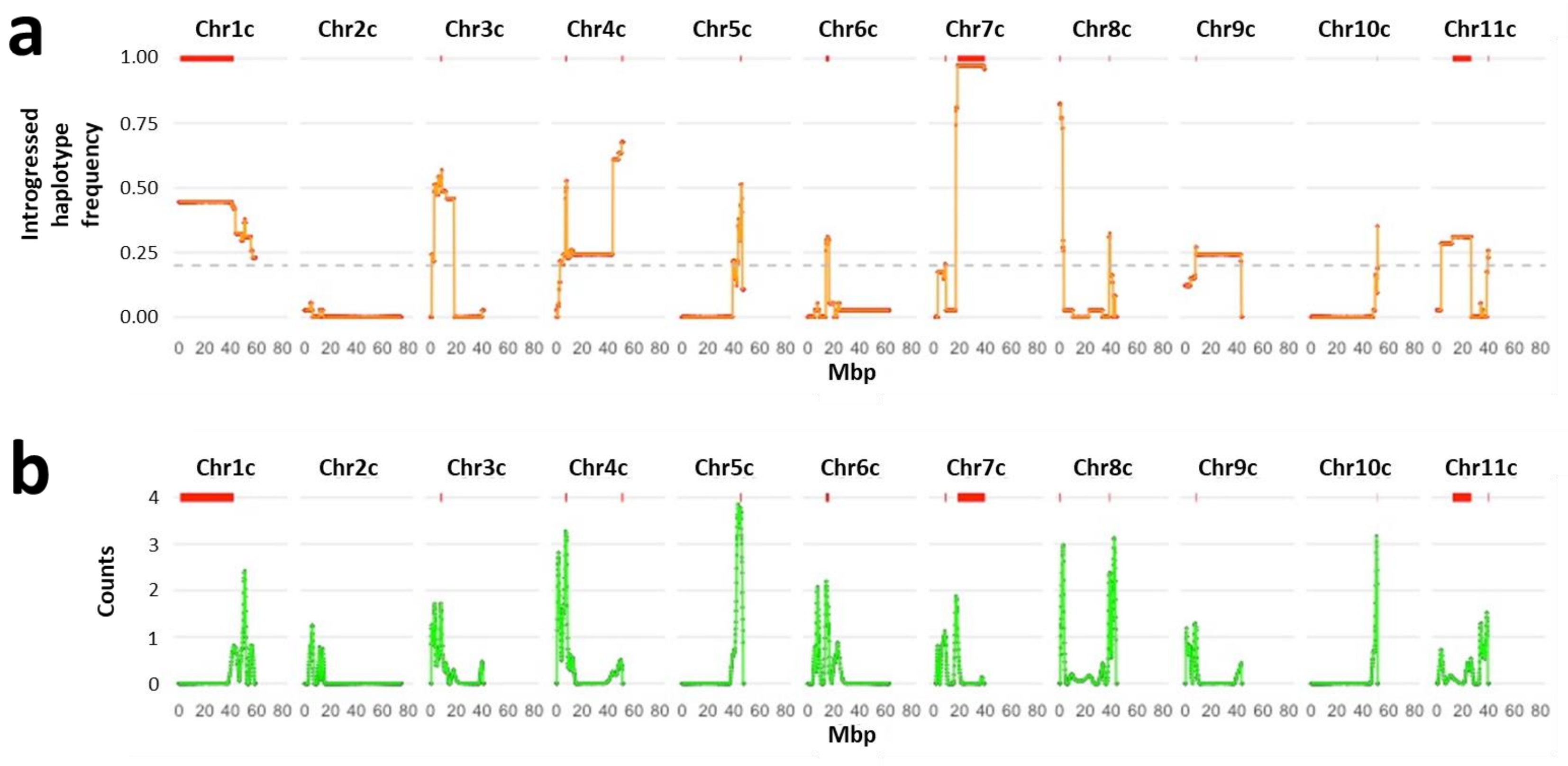

Supplementary Fig. 47. Timor hybrid-derived introgression in a group of 37 expected Timor hybrid derivatives. a Timor hybrid-derived haplotype frequency in the canephora subgenome. **b** Number of independent events of recombination in introgressed chromosome segments per million bases. Introgressed haplotype frequency in **a** was calculated across 2,212 non-overlapping genomic windows containing 100 Kb of non-repetitive DNA. Red horizontal bars on top of each panel indicate the regions used for GO enrichment analysis (Supplementary Data 7) corresponding to intrachromosomal peaks of Timor hybrid-derived haplotype frequency. Only peaks above the 0.20 threshold of Timor hybrid-derived haplotype frequency (shown as horizontal gray dashed-line in **a**) were considered for GO enrichment analysis. Evidence of recombination occurring over the same genomic window in multiple accessions was considered as originating from one single ancestral shared recombinational event. Source data are provided as a Source Data file.

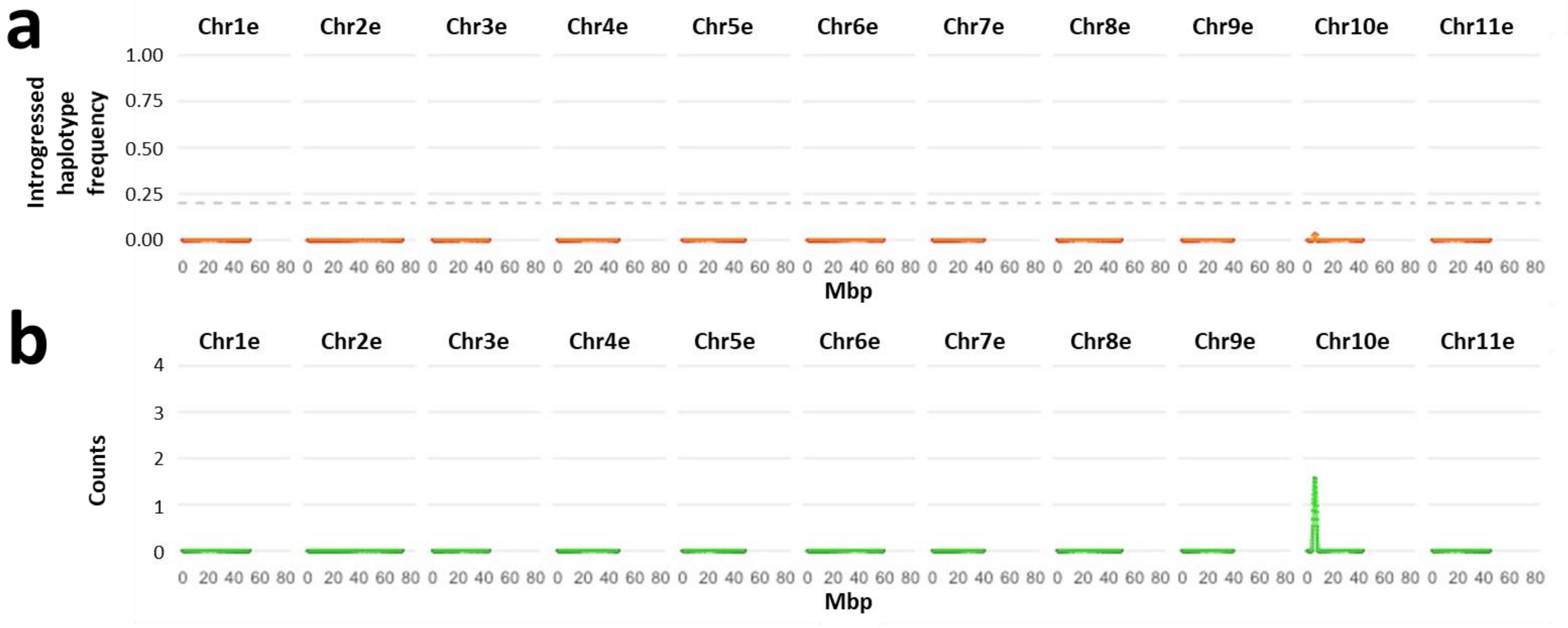

Supplementary Fig. 48. Timor hybrid-derived introgression in a group of 37 expected Timor hybrid derivatives. a Timor hybrid-derived haplotype frequency in the eugenioides subgenome. **b** Number of independent events of recombination in introgressed chromosome segments per million bases. It has to be noted that the upper end of Chr10e contains a subtelomeric canephora-derived segment as a result of a reciprocal homoeologous exchange described in the main text. Introgressed haplotype frequency in **a** was calculated across 2,255 non-overlapping genomic windows containing 100 Kb of non-repetitive DNA. Red horizontal bars on top of each panel indicate the regions used for GO enrichment analysis (Supplementary Data 7) corresponding to intrachromosomal peaks of Timor hybrid-derived haplotype frequency. Only peaks above the 0.20 threshold of Timor hybrid-derived haplotype frequency (shown as horizontal gray dashed-line in **a**) were considered for GO enrichment analysis. Evidence of recombination occurring over the same genomic window in multiple accessions was considered as originating from one single ancestral shared recombinational event. Source data are provided as a Source Data file.

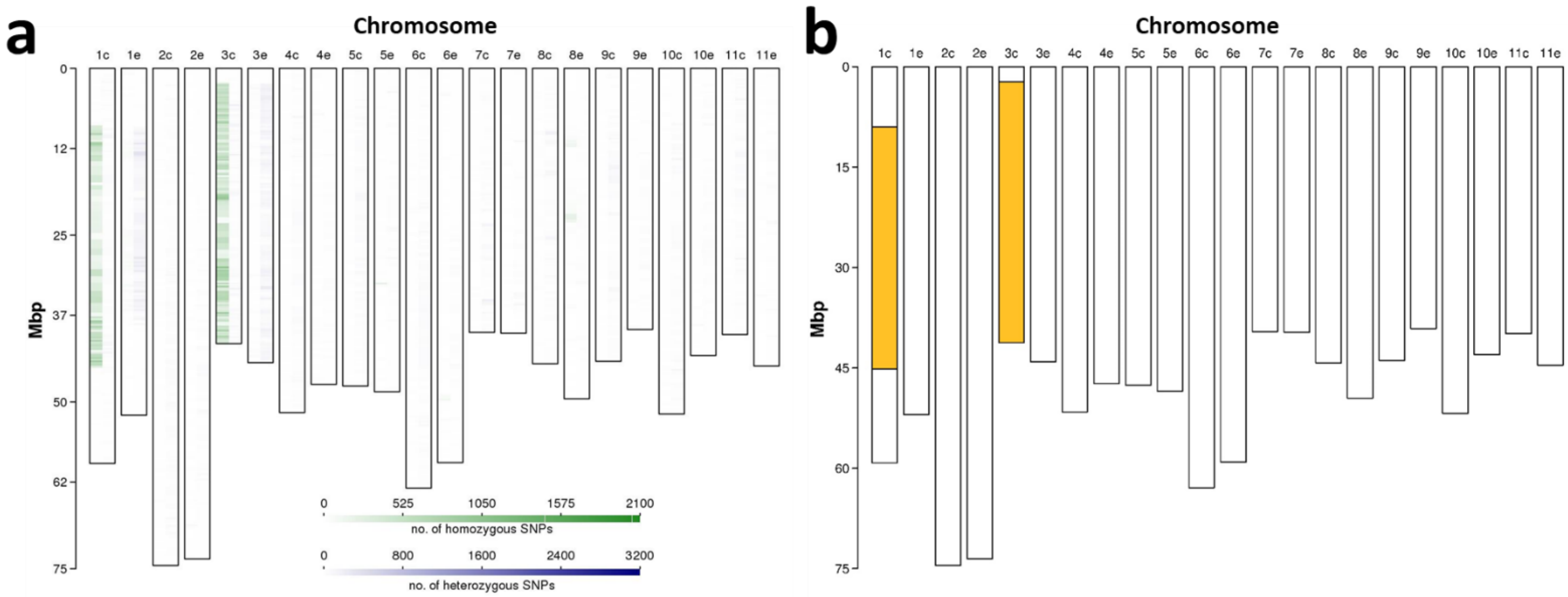

Supplementary Fig. 49. *Coffea* sp. introgression in the accession S288[20]. **a** Densities of heterozygous (plotted in the right-hand sector of each ideogram) and homozygous (plotted in the left-hand sector of each ideogram) SNPs with respect to the Bourbon reference are shown by blue-to-white and green-to-white heatmaps, respectively. Densities represent the number of SNPs per genomic window of 100 Kb of non-repetitive DNA. **b** Genomic location of *Coffea sp.* introgressed chromosome segments. In both panels, ideograms represent chromosomes. The y-axes indicate chromosome length in million base pairs (Mbp). Source data are provided as a Source Data file.

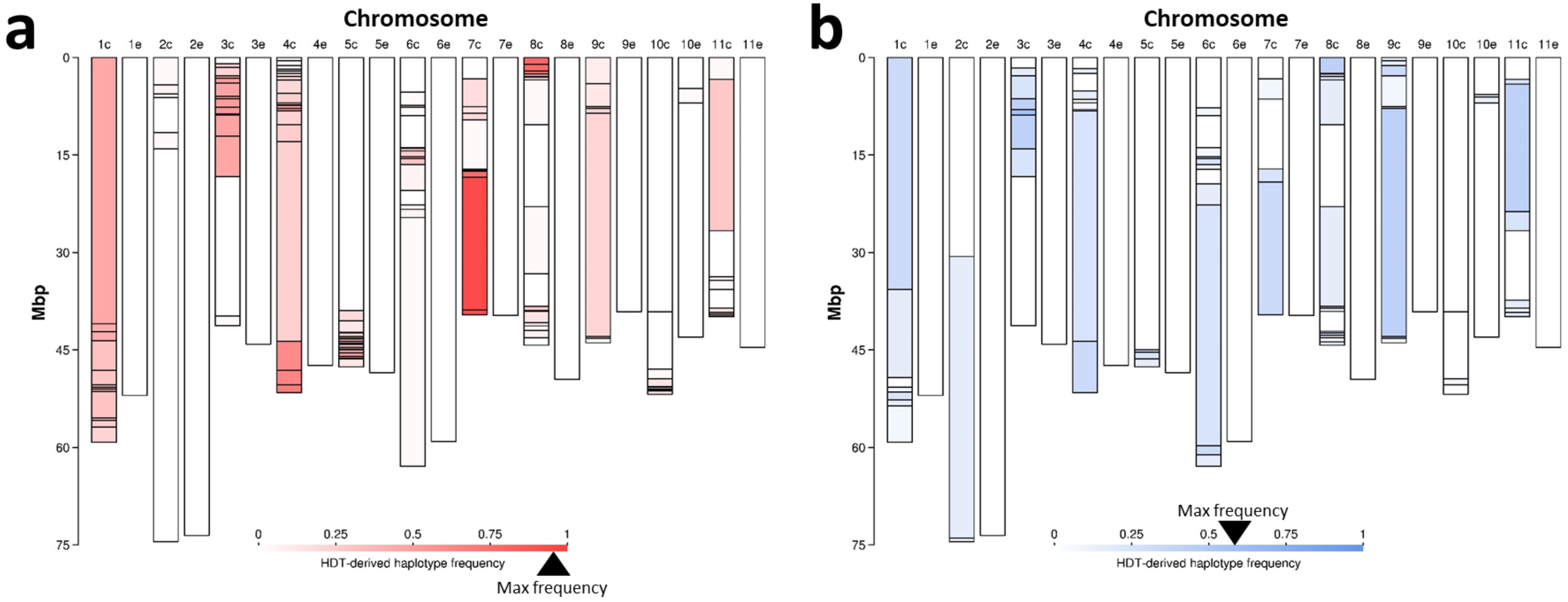

Supplementary Fig. 50. Genomic location of the recombination breakpoints of *C. canephora*-introgressed chromosome segments. a Recombination breakpoints in 37 expected Timor hybrid derivatives**. b** Recombination breakpoints in 6 specimens carrying unexpected canephora introgression (i.e. Kent, SL28, SL34, CHF1, GNG1 and GUG3). In both panels, ideograms represent chromosomes. The y-axis indicates chromosome length in million base pairs (Mbp). Heatmaps show Timor hybrid-derived haplotype frequency in the two sets. Black arrowheads in the heatmap legend point to the highest observed haplotype frequency in each set (Max frequency). Source data are provided as a Source Data file.

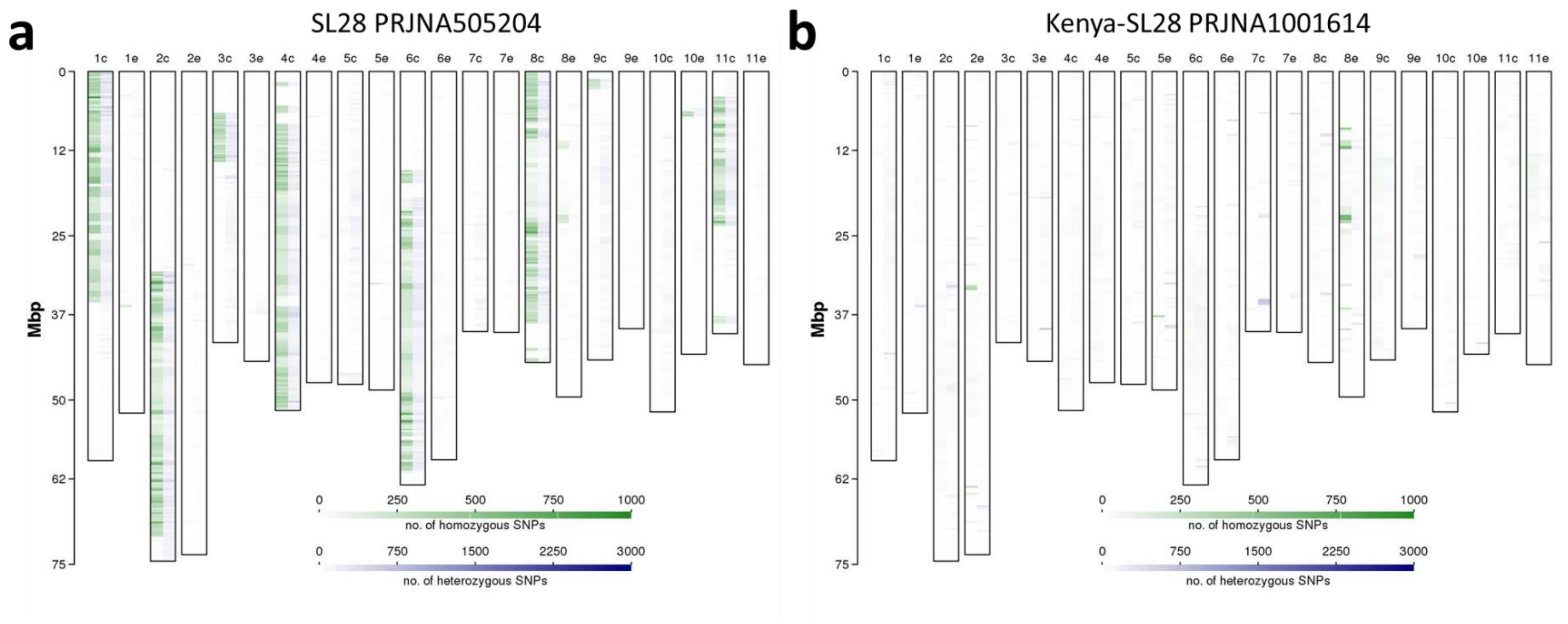

Supplementary Fig. 51. SNP density plot in two resequenced accessions of SL28 (Supplementary Table 9 and Supplementary Data 1). a Resequencing data deposited in NBCI under the BioProject PRJNA505204[20] (modal genome coverage = 2X). **b** Resequencing data generated in the present paper using an accession introduced from CATIE (modal genome coverage = 34X). In both panels, ideograms represent chromosomes. The y-axis indicates chromosome length in million base pairs (Mbp). Heat maps indicate densities of heterozygous (blue) and homozygous (green) SNPs with respect to the Bourbon reference. Heat maps were set to a maximum of 1,000 homozygous SNPs and 3,000 heterozygous SNPs per genomic window of 100 Kb of non-repetitive DNA. Values greater than maximum were plotted as maximum.

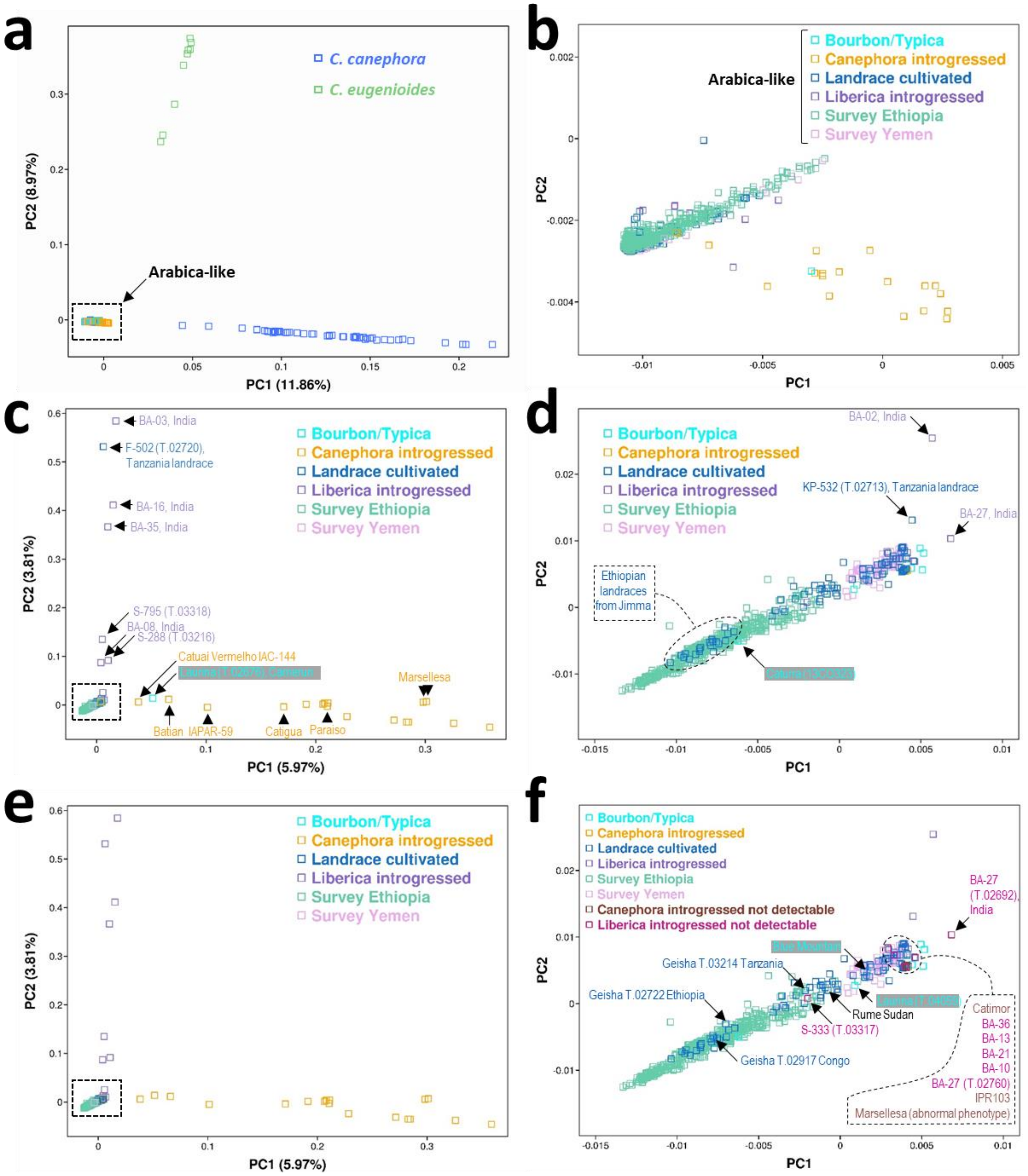

Supplementary Fig. 52. Genetic diversity in 834 *Coffea* sp. accessions based on GBS data. a PCA based on 8,169 SNPs in 834 accessions that include 54 accessions of *C. canephora*, 9 accessions of *C. eugenioides* and 771 Arabica-like accessions, the latter sorted into 6 categories based on *a priori* classification given by the holding germplasm repositories[13] (Supplementary Data 3). **b** Magnified view of the section of the bidimensional plot delimited by the rectangle in **a** representing Arabica-like germplasm. **c** PCA based on 1,992 SNPs in 771 Arabica-like

accessions sorted into 6 categories based on *a priori* classification given by the holding germplasm repositories. **d** Magnified view of the section of the bidimensional plot delimited by the rectangle in **c** representing Arabica-like germplasm. **e** The same as in **c** with the exception that accessions that turned out to be introgression lines were reclassified as such. **f** The same as in **d** with the exception that Arabica-like accessions were sorted into 8 categories based on the combination of *a priori* classification given by the holding germplasm repositories and genomic-based evidence of the presence of *C. canephora* or *C. liberica* introgression. In case of unexpected genomic-based evidence of introgression in accessions that were not classified *a priori* as such by the holding germplasm repositories (cryptic introgression), the accession was reclassified. In case of absence of evidence from genomic-based analysis of any introgressed segment in accessions that were classified *a priori* by the holding germplasm repositories as introgression lines based on their pedigree, the accession maintained the original classification with the appended note that introgression could not be detected. In the latter case, we could not exclude the possibility that introgression was completely purged by backcrossing or remained confined in relatively small chromosome segments in pericentromeric regions that could be poorly mapped by dRAD sequencing. In all panels, the bidimensional plot illustrates the first two components of a Principal Component Analysis (PCA). Source data are provided as a Source Data file.

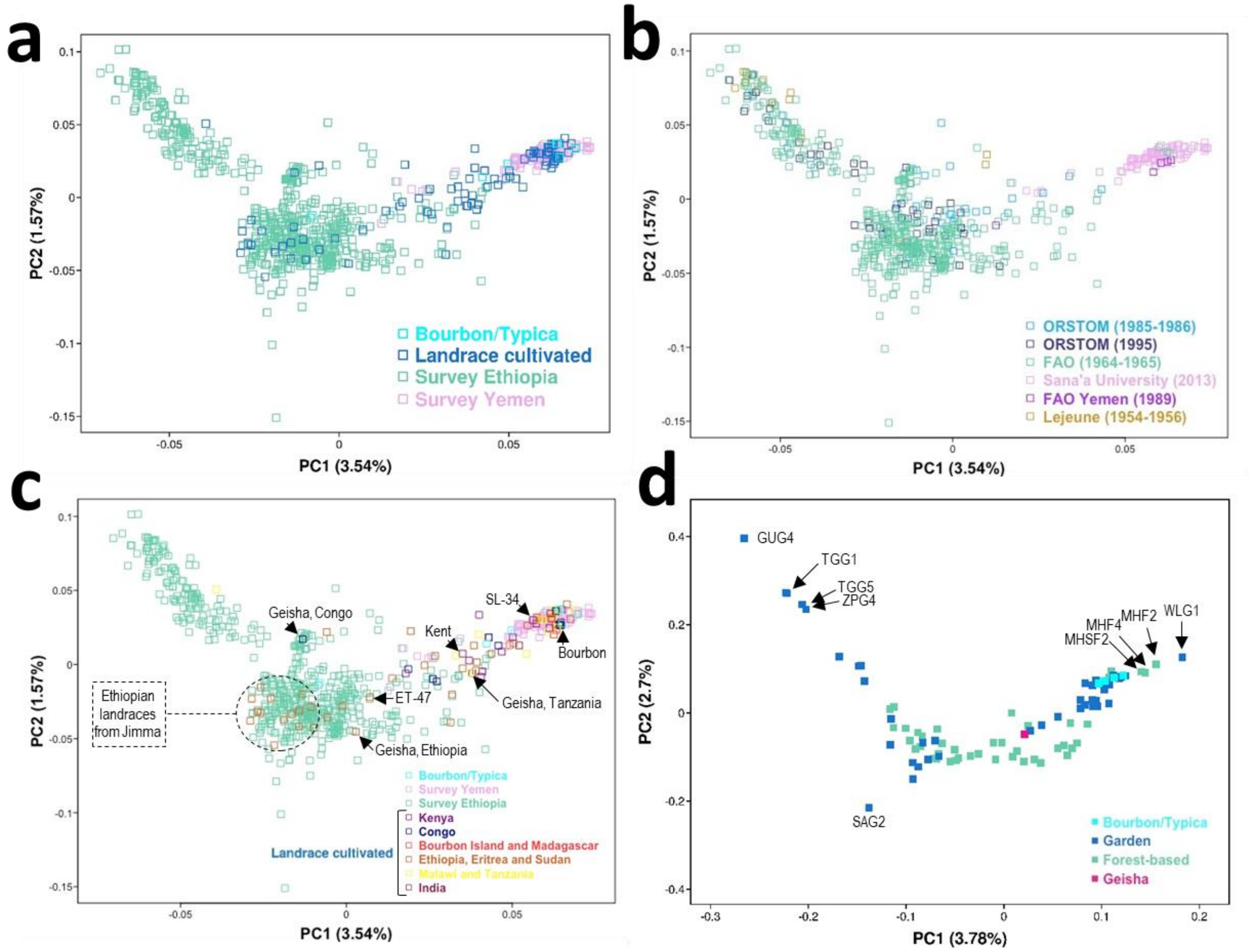

Supplementary Fig. 53. Genetic diversity in 734 *bona fide C. arabica* accessions based on GBS data and in 94 *bona fide C. arabica* accessions based on WGS data. a-c PCA based on 1,397 SNPs using publicly available dRAD sequencing data[13]. **a** Accession type as originally given by Scalabrin and colleagues[13]. **b** Accessions were sorted on the basis of the germplasm prospection during which they were collected. **c** Cultivated landraces were sorted based on the country where they were collected. Neighbouring countries are indicated with a single colour code. Relevant varieties in the GBS panel that are associated with critical raw read datasets in the WGS panel are indicated in **c** by black arrows and compared with the location on the PCA space of ‘Bourbon’, on one side, and with the location of Ethiopian landraces from the area around Jimma in the South West Region (where *C. arabica* is reported to grow spontaneously in rain forests[21]), on the other side, along the variance explained by PC1. The entry IDs given by the CATIE germplasm repository for the three accessions of ‘Geisha’, in this graph uniquely identified by their country of introduction, are given in Supplementary Data 3. **d** PCA based on 1,877,440 SNPs generated from WGS in this study. Accessions used in garden-based and forest-based coffee production systems are defined according to Mekbib and colleagues[19] (Supplementary Data 1). In all panels, the bidimensional plot illustrates the first two components of a Principal Component Analysis (PCA). Source data are provided as a Source Data file.

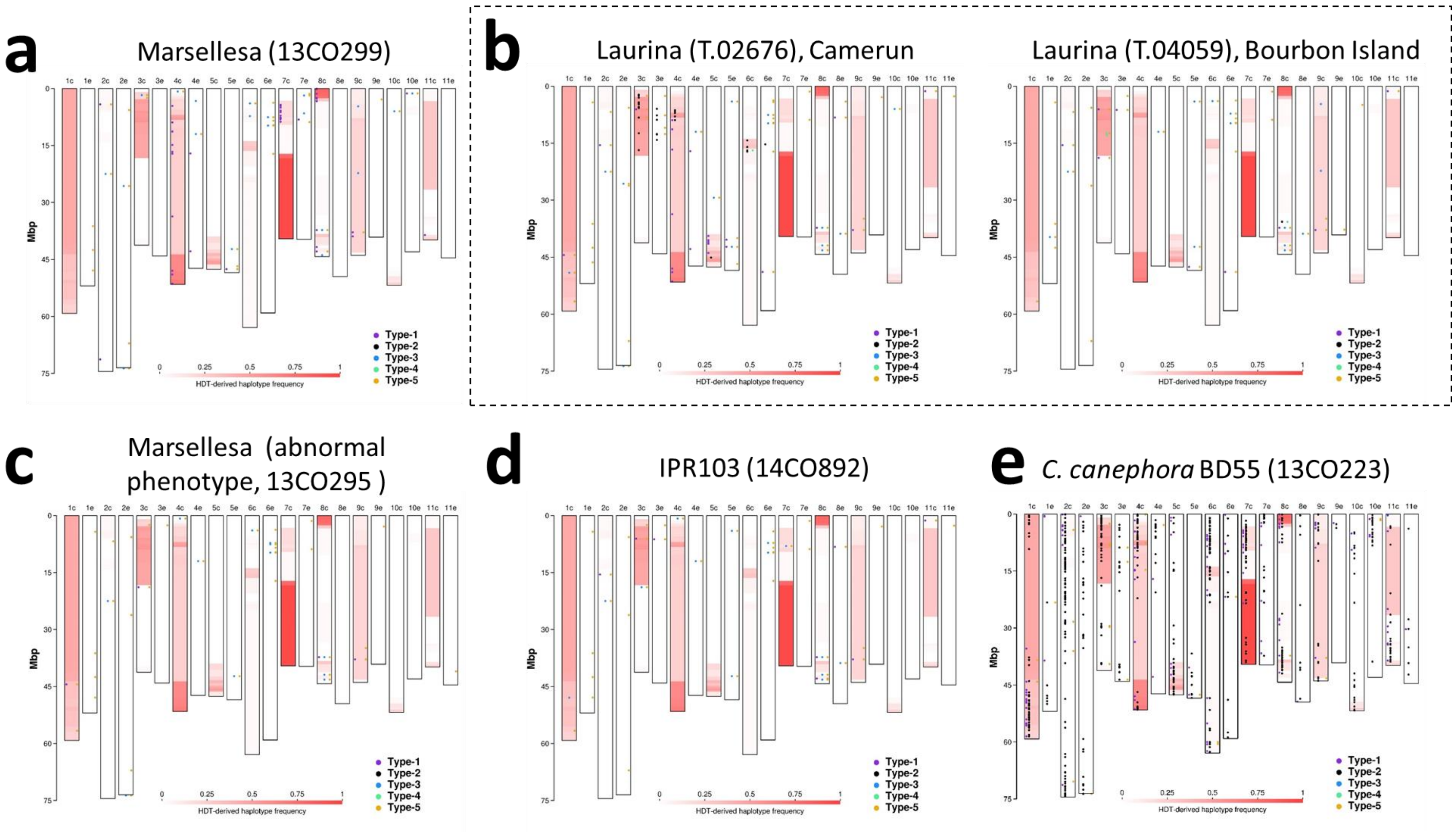

Supplementary Fig. 54. Chromosomal plots of *C. canephora* introgression into *C. arabica* genetic backgrounds based on WGS and GBS analysis. a Known and genomic-validated introgression lines. **b** Unexpected introgression lines with detectable introgression. **c-d** Expected introgression lines that possibly reverted to pure *C. arabica* genomes (loss of introgressed haplotypes) as a result of sexual propagation within the variety (**c**) or as a result of multiple cycles of backcrossing in the obtainment of the variety (**d**). **e** A diploid *C. canephora* accession used as a positive control. In all panels, ideograms represent chromosomes. The y-axis indicates

chromosome length in million base pairs (Mbp). The heatmap in the background shows introgressed haplotype frequency across 4,467 non-overlapping genomic windows containing 100 Kb of non-repetitive DNA based on WGS data in a set of 37 canephora introgression lines (Timor hybrid derivatives) as shown in Supplementary Fig. 46. Colored dots indicate the chromosomal location of SNPs identified in each accession based on GBS data. Type-1 SNPs (violet dots plotted in the leftmost lane of each diagram) are variant sites in the accession that were polymorphic in the population of *C. canephora* and were shared with one or more expected canephora introgression lines of HDT derivatives. Type-2 SNPs (black dots plotted in the second leftmost lane of each diagram) are variant sites in the accession that were polymorphic in the population of *C. canephora* but they were not shared with any expected introgression line of HDT derivatives. Type-3 SNPs (blue dots plotted in the central lane of each diagram) are variant sites in the accession that were shared with one or more expected introgression line of HDT derivatives but they were identical to the Bourbon reference in the population of *C. canephora*. Type-4 SNPs (green dots plotted in the second rightmost lane of each diagram) are variant sites in the accession that were not shared with the population of *C. canephora* and with expected canephora introgression lines of HDT derivatives. Type-5 SNPs (ochre dots plotted in the rightmost lane of each diagram) are variant sites in the accession that were shared with one or more expected liberica introgression lines. Source data are provided as a Source Data file.

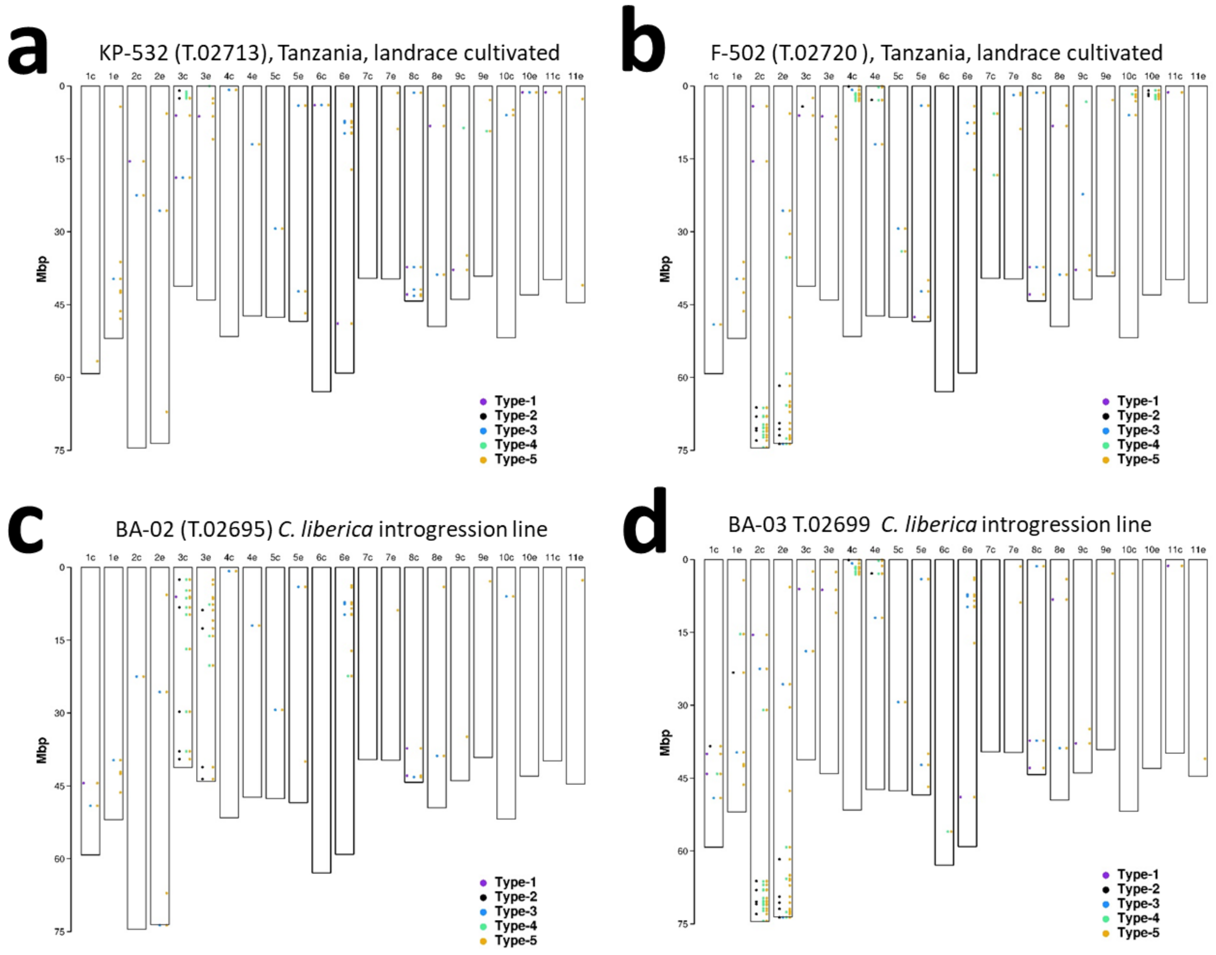

Supplementary Fig. 55. Chromosomal plots of *C. liberica* introgression into *C. arabica* genetic backgrounds based on GBS analysis. a-b Unexpected introgression lines with detectable introgression. **c-d** Known and genomic-validated introgression lines. In all panels, ideograms represent chromosomes. The y-axis indicates chromosome length in million base pairs (Mbp). Colored dots indicate the chromosomal location of SNPs identified in each accession based on GBS data. Type-1 SNPs (violet dots plotted in the leftmost lane of each diagram) are variant sites in the accession that were polymorphic in the population of *C. canephora* and were shared with one or more expected canephora introgression lines of HDT derivatives. Type-2 SNPs (black dots plotted in the second leftmost lane of each diagram) are variant sites in the accession that were polymorphic in the population of *C. canephora* but they were not shared with any expected introgression line of HDT derivatives. Type-3 SNPs (blue dots plotted in the central lane of each diagram) are variant sites in the accession that were shared with one or more expected introgression line of HDT derivatives but they were identical to the Bourbon reference in the population of *C. canephora*. Type-4 SNPs (green dots plotted in the second rightmost lane of each diagram) are variant sites in the accession that were not shared with the population of *C. canephora* and with expected canephora introgression lines of HDT derivatives. Type-5 SNPs (ochre dots plotted in the rightmost lane of each diagram) are variant sites in the accession that were shared with one or more expected liberica introgression lines.

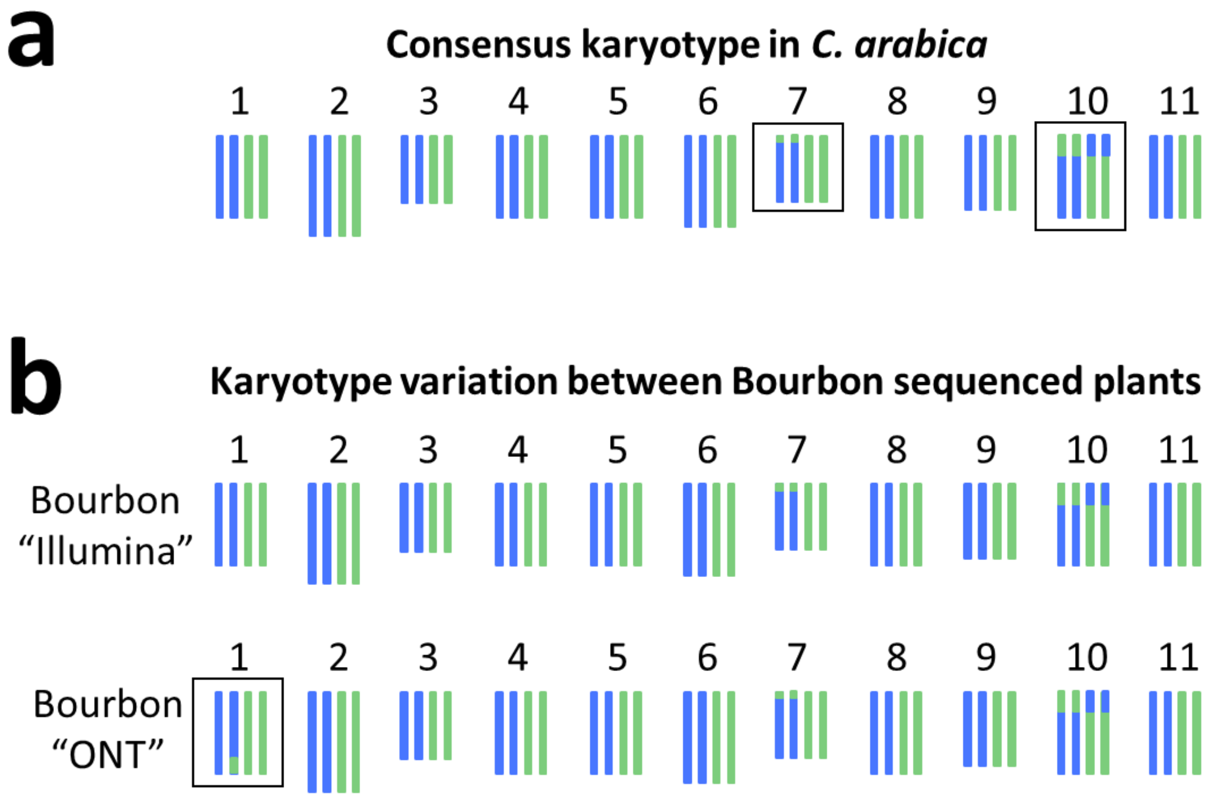

Supplementary Fig. 56. Graphical representation of karyotypes in *C. arabica*. a The most common karyotype in the analysed germplasm of *C. arabica*. **b** Variation between two individual plants of Bourbon used, separately, for short-read sequencing ("Illumina") and for long-read sequencing ("ONT"). Thin-lined boxes indicate other large (>200 Kb) homoeologous exchanges occurring in these accessions. Blue and green vertical ideograms represent canephora and eugenioides homoeologous copies, respectively. Ideograms are not drawn to scale. The exact length of chromosome pseudomolecules and the chromosome segment with unbalanced homoeologous copies in **b** are reported in Supplementary Table 2 and Supplementary Data 5. The plots of intrachromosomal variation in depth of coverage from which these ideograms are inferred are provided in Supplementary Fig. 41.

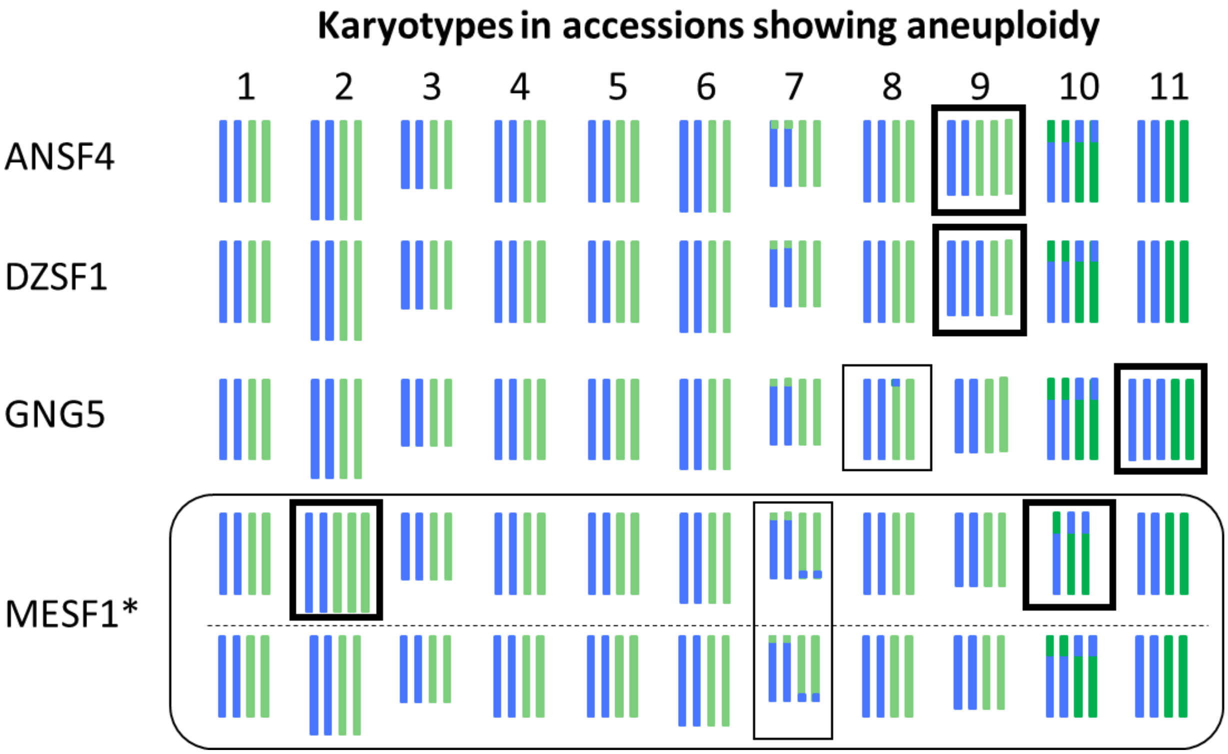

Supplementary Fig. 57. Graphical representation of karyotypes in 4 accessions showing aneuploidies. Thick-lined boxes indicate trisomy and monosomy. Thin-lined boxes indicate other large (>200 Kb) homoeologous exchanges occurring in these accessions. Blue and green vertical ideograms represent canephora and eugenioides homoeologous copies, respectively. Ideograms are not drawn to scale. The exact length of chromosome pseudomolecules is reported in Supplementary Table 2. The plots of inter- and intra-chromosomal variation in depth of coverage and homoeologous variant frequency from which these ideograms are inferred available in the figshare database[17].

* Homoeologous Variant Frequency plots and Depth of Coverage plots are compatible with MESF1 being a somatic mosaicism or MESF1 DNA resulting from the mixture of DNAs extracted from two different accessions, one of which carried trisomic Chr2e and monosomic Chr10c[17]. Based on the level of deviation in Homoeologous Variant Frequency we estimated the proportion of cells MESF1 carrying the variant in mosaic tissues or the ration of sample DNA contamination at 30 % with aneuploidy and 70 % wild-type based on a modified version of the formula reported by Marroni and colleagues[18], adjusted for polyploids in order to account here for tetraploidy.

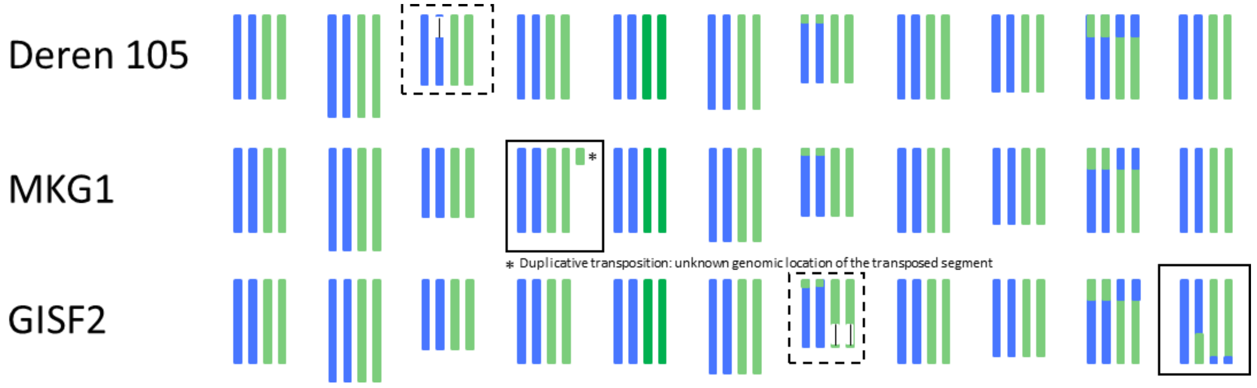

Supplementary Fig. 58. Graphical representation of the karyotypes in *C. arabica* accessions that differ from the most common karyotype shown in Supplementary Fig. 56 for one or more large segmental duplications and deletions. Dashed-lined boxes indicate large segmental duplications and deletions. Thin-lined boxes indicate large (>200 Kb) homoeologous exchanges occurring in these accessions. Blue and green vertical ideograms represent canephora and eugenioides homoeologous copies, respectively. Ideograms are not drawn to scale. The exact length of chromosome pseudomolecules and the chromosome segments with unbalanced homoeologous copies are reported in Supplementary Table 2 and Supplementary Data 4. The plots of inter- and intra-chromosomal variation in depth of coverage and homoeologous variant frequency from which these ideograms are inferred are available in the figshare database[17].

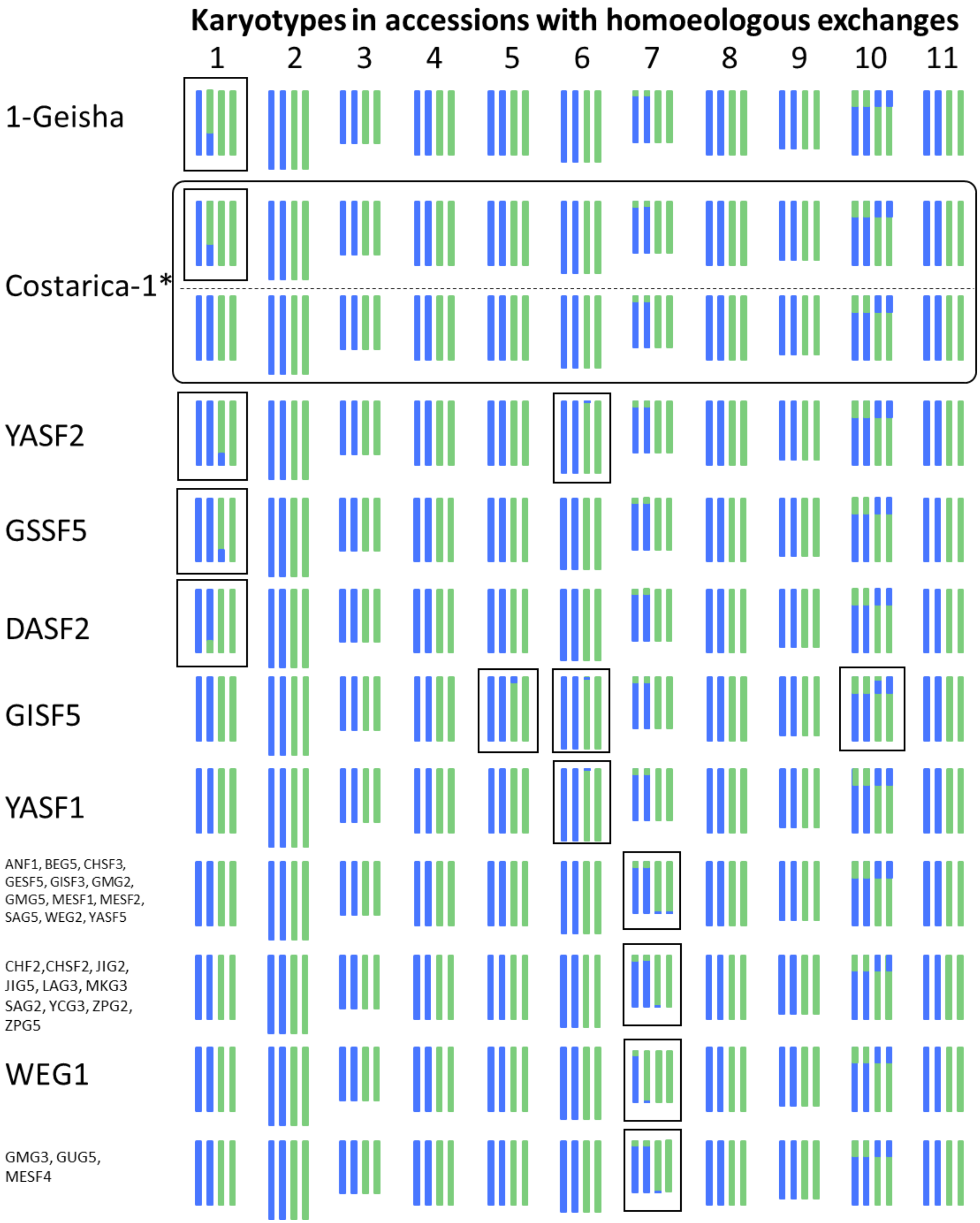

Supplementary Fig. 59. Graphical representation of the karyotypes in *C. arabica* accessions that differ from the most common karyotype shown in Supplementary Fig. 56 for one or more homoeologous exchanges shown in Fig. 5. Thin-lined boxes indicate large (>200 Kb)

homoeologous exchanges occurring in these accessions. Blue and green vertical ideograms represent canephora and eugenioides homoeologous copies, respectively. Ideograms are not drawn to scale. The exact length of chromosome pseudomolecules and the chromosome segments with unbalanced homoeologous copies are reported in Supplementary Table 2 and Supplementary Data 5-6. The plots of inter- and intra-chromosomal variation in depth of coverage and homoeologous variant frequency from which these ideograms are inferred are available in the figshare database[17].

* Homoeologous Variant Frequency plots and Depth of Coverage plots are compatible with Costarica-1 being a somatic mosaicism or Costarica-1 DNA resulting from the mixture of DNAs extracted from two different accessions, one of which carried trisomic Chr2e and monosomic Chr10c[17]. Based on the level of deviation in Homoeologous Variant Frequency we estimated the proportion of cells Costarica-1 carrying the variant in mosaic tissues at 50 % with the homoeologous exchange and 50 % wild-type, based on a modified version of the formula reported by Marroni and colleagues[18], adjusted for polyploids in order to account here for tetraploidy .

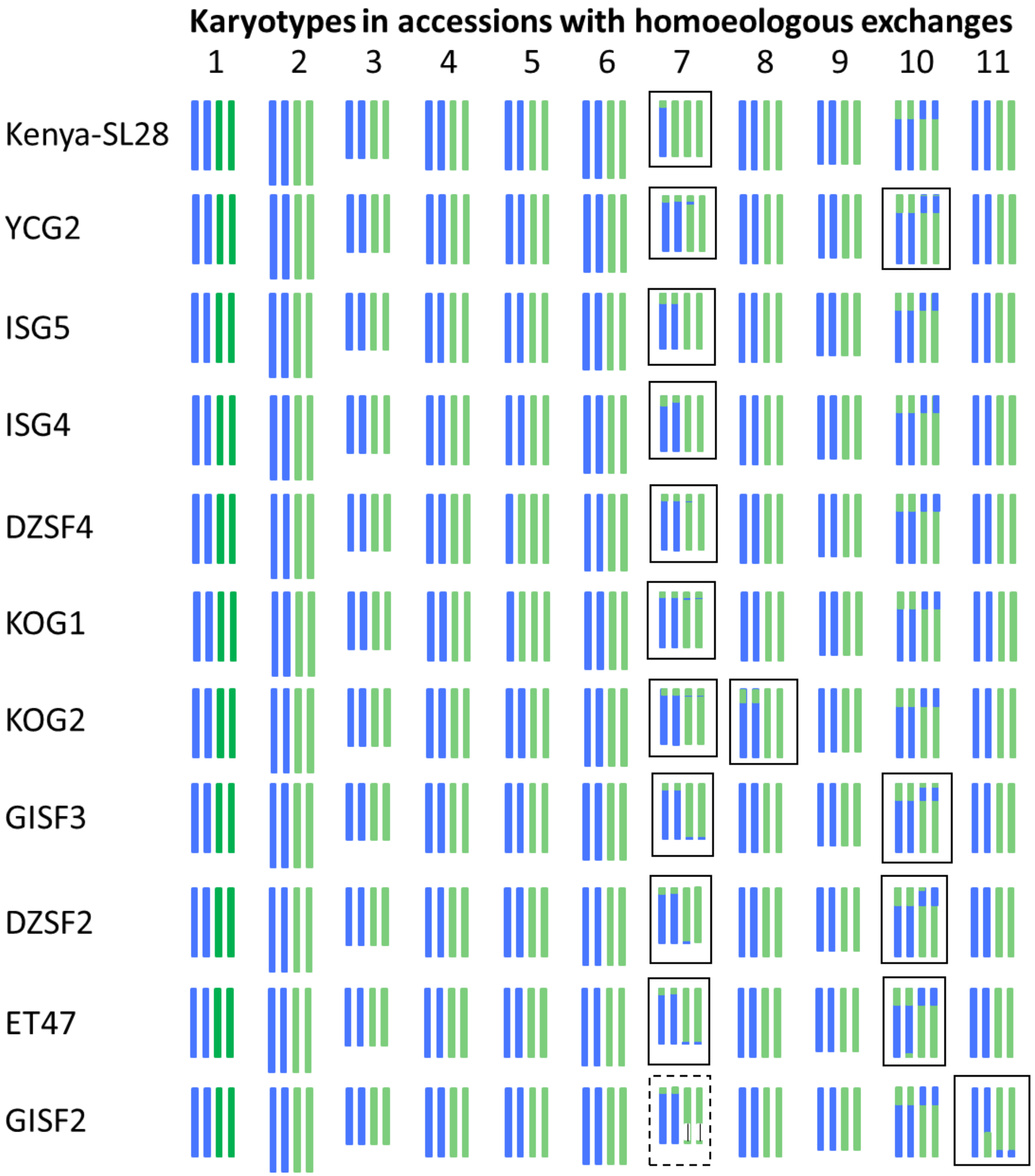

Supplementary Fig. 60. Graphical representation of the karyotypes in *C. arabica* accessions that differ from the most common karyotype shown in Supplementary Fig. 56 for one or more homoeologous exchanges shown in Fig. 5. Dashed-lined boxes indicate large segmental duplications and deletions. Thin-lined boxes indicate large (>200 Kb) homoeologous exchanges occurring in these accessions. Blue and green vertical ideograms represent canephora and eugenioides homoeologous copies, respectively. Ideograms are not drawn to scale. The exact length of chromosome pseudomolecules and the chromosome segments with unbalanced

homoeologous copies are reported in Supplementary Table 2 and Supplementary Data 5-6. The plots of inter- and intra-chromosomal variation in depth of coverage and homoeologous variant frequency from which these ideograms are inferred are available in the figshare database[17].

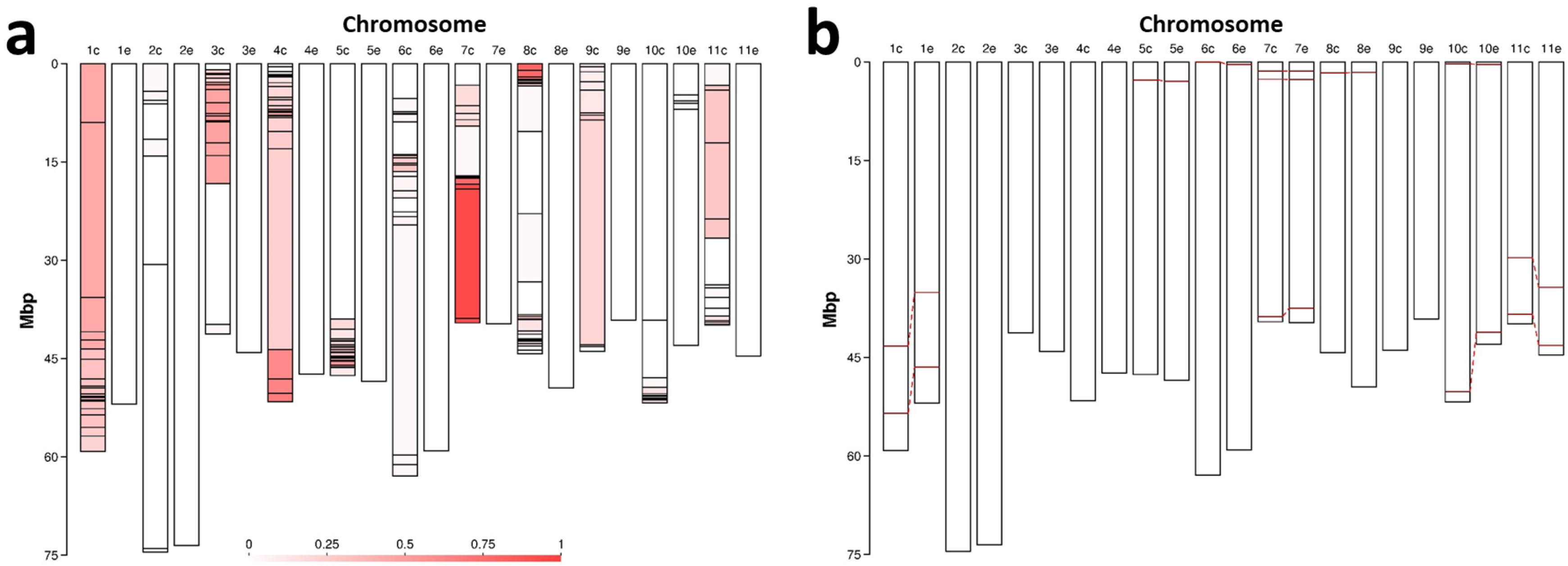

Supplementary Fig. 61. Genomic location of the events that generated chromosomal rearrangements. a Breakpoints of homologous recombination between *C. arabica*-derived homologs and modern diploid *Coffea* sp-derived homologs in *C. arabica* × *Coffea* sp. introgression lines. The heatmap in **a** shows Timor hybrid-derived haplotype frequency. **b** Sites of homoeologous exchanges in *C. arabica*. In both panels, ideograms represent chromosomes. The y-axis indicates chromosome length in million base pairs (Mbp). Source data are provided as a Source Data file and as Supplementary Data 6.

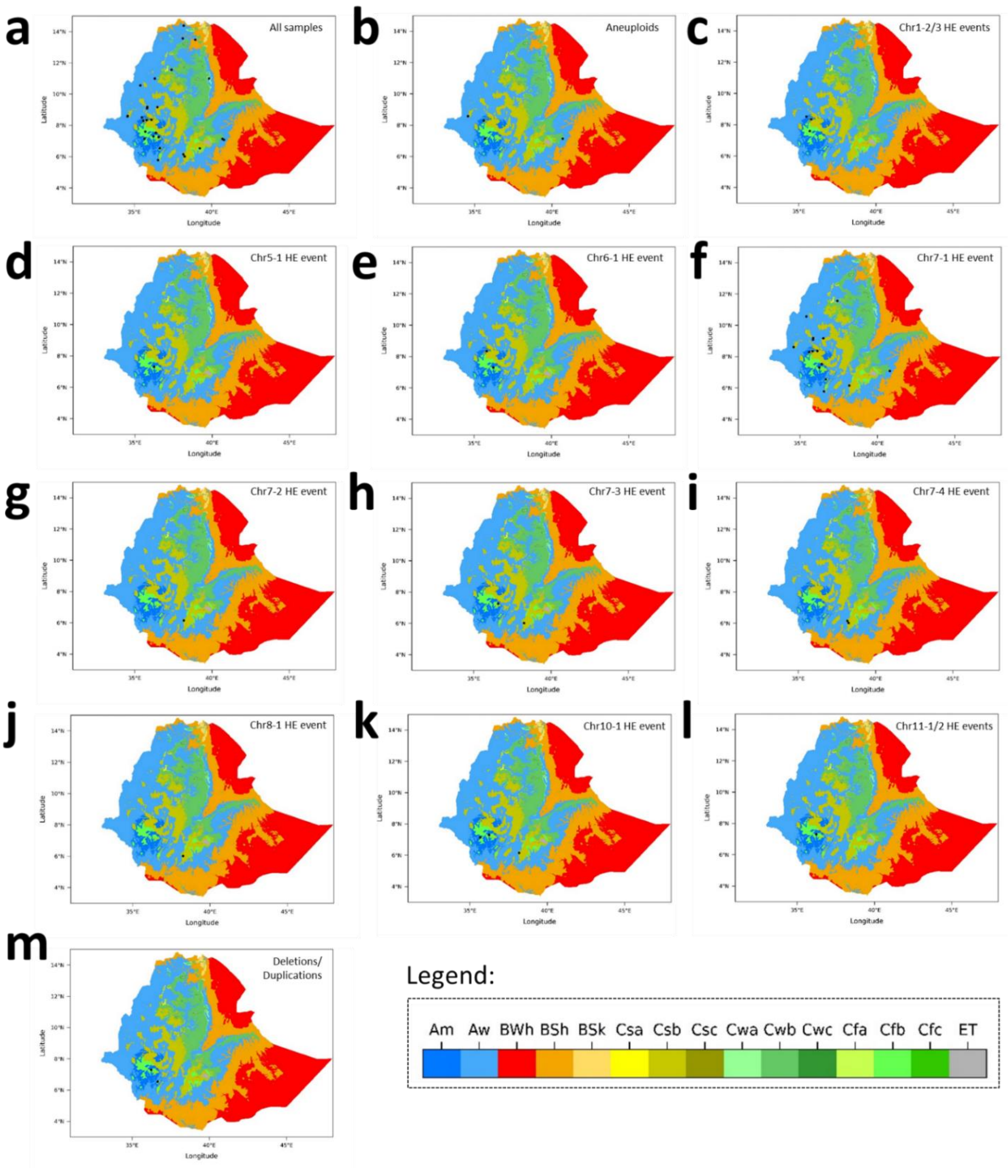

Supplementary Fig. 62. Geographic origin in Ethiopia of the accessions sequenced by Mekbib and colleagues[19] and analyzed in the present study. a All sampling sites. **b** Sampling sites of the accessions that showed independent events of aneuploidy. **c-l** Sampling sites of the accessions that showed homoeologous exchanges. **m** Sampling sites of the accessions that showed deletions/duplication. The approximate geographic coordinates were obtained from the location of the sampling sites (back dots) reported by Mekbib and colleagues[19]. Colours in the

background represent a climate map based on the Köppen-Geiger climate classification[22]. Symbols in the colour key stand for: A (Tropical), m (Monsoon), w (Savanna, dry winter); B (Dry), W (Arid Desert), S (Semi-Arid or steppe), h (Hot), k (Cold). C (Temperate), w (Dry winter), f (No dry season), s (Dry summer), a (Hot summer), b (Warm summer), c (Cold summer); E (Polar), T (Tundra). It has to be noted that the few spots in the map corresponding to Polar Tundra likely represent inaccurate data points in the original data[23]. The climate map in the background of panels **a-m** was plotted using the R libraries colorspace, tidyverse, raster, openxlsx, sf, ggspatial, gridExtra, ggrepel, ggplot2, cowplot and RColorBrewer using the data deposited in figshare public repository[23] for the Köppen-Geiger climate classification.

Supplementary Table 1. Metrics of the assembly.

Metrics	Contigs (n)	Contigs %	Scaffolds (n)	Scaffolds %	Superscaffolds (n)	Superscaffolds %
Number of sequences	2,131	-	1,995	-	1,751	-
Total size of sequences (nt)	1,338,463,224	-	1,338,531,224	-	1,317,750,309	-
Longest sequence (nt)	39,538,567	-	161,147,661	-	74,718,367	-
Shortest sequence (nt)	11,872	-	11,872	-	11,872	-
Number of sequences > 1kb	2,131	100	1,995	100	1,751	100
Number of sequences > 10kb	2,131	100	1,995	100	1,751	100
Number of sequences > 100kb	1,356	63.6	1,241	62.2	1041	59.5
Number of sequences > 1Mb	129	6.1	69	3.5	27	1.5
Number of sequences > 10Mb	37	1.7	29	1.5	22	1.3
Mean sequence size (nt)	628,092	-	670,943	-	752,570	-
Median sequence size (nt)	112,279	-	110,130	-	107,185	-
N50 sequence length (nt)	10,187,730	-	24,847,274	-	47,362,295	-
L50 sequence count	37	-	13	-	12	-

Supplementary Table 2. Metrics of the assembly, by chromosome.

Chromosome	Superscaffolds (n)	Scaffolds (n)	Contigs (n)	Sequence length (bp)
Chr1c	1	8	17	59,282,136
Chr1e	1	1	12	52,005,836
Chr2c	1	3	3	74,718,367
Chr2e	1	3	14	73,705,077
Chr3c	1	1	6	41,337,678
Chr3e	1	2	7	44,118,139
Chr4c	1	7	8	51,611,264
Chr4e	1	4	11	47,362,295
Chr5c	1	4	4	47,630,375
Chr5e	1	4	10	48,524,476
Chr6c	1	2	5	62,962,097
Chr6e	1	7	15	59,109,944
Chr7c	1	1	1	39,944,450
Chr7e	1	3	4	39,730,949
Chr8c	1	5	9	44,319,397
Chr8e	1	1	5	49,651,447
Chr9c	1	3	4	43,933,066
Chr9e	1	4	7	39,158,766
Chr10c	1	7	12	51,900,066
Chr10e	1	3	8	43,085,031
Chr11c	1	4	5	39,995,608
Chr11e	1	3	8	44,702,780
Sum	22	80	175	1,098,789,244
chloroplast	1	1	1	153,987
mitochondrion	1	1	2	1,172,695
Unanchored sequences	1,727	1,913	1,771	217,634,383
**Total assembly**	**1,751**	**1,995**	**1,949**	**1,317,750,309**

Supplementary Table 3. Chromosome pseudomolecules, unanchored scaffolds and comparison with the state of art Caturra assembly (GCA_003713225.1).

	Bourbon assembly (this paper)		Caturra assembly (GCA_003713225.1)		Bourbon to Caturra
**Chromosome pseudomolecule**	**Length (nt)**	**Gaps (n)**	**Length (nt)**	**Gaps (n)**	**Relative pseudomolecule length**
Chr1c	59,282,136	16	50,636,588	84	117%
Chr2c	74,718,367	2	66,155,350	21	113%
Chr3c	41,337,678	5	41,566,753	10	99.4%
Chr4c	51,611,264	7	41,786,336	36	124%
Chr5c	47,630,375	3	45,899,693	11	104%
Chr6c	62,962,097	4	55,181,588	19	114%
Chr7c	39,944,450	0	38,854,053	34	103%
Chr8c	44,319,397	8	39,008,463	34	114%
Chr9c	43,933,066	3	38,064,651	65	115%
Chr10c	51,900,066	11	45,429,025	45	114%
Chr11c	39,995,608	4	36,215,491	21	110%
Chr1e	52,005,836	11	48,756,970	38	107%
Chr2e	73,705,077	13	71,633,312	23	103%
Chr3e	44,118,139	6	37,271,464	11	118%
Chr4e	47,362,295	10	42,507,429	17	111%
Chr5e	48,524,476	9	39,439,615	16	123%
Chr6e	59,109,944	14	52,240,725	50	113%
Chr7e	39,730,949	3	35,392,230	13	112%
Chr8e	49,651,447	4	45,117,557	13	110%
Chr9e	39,158,766	6	35,839,895	12	109%
Chr10e	43,085,031	7	40,458,934	24	106%
Chr11e	44,702,780	7	42,465,768	26	105%
Sum	1,098,789,244	153	989,921,890	623	111%
**Unanchored scaffolds**	217,634,383				
**Total assembly**	1,316,423,627				

Supplementary Table 4. Consistency in order and orientation of chromosome pseudomolecules with the assemblies of the diploid progenitors.

	Bourbon assembly (this paper) vs diploid assemblies				Caturra assembly (GCA_003713225.1) vs diploid assemblies			
**Chromosome pseudomolecule**	**Length (nt)**	**Inconsistency (no. of intervals)**	**Inconsistency (cumulative nt)**	**Relative inconsistency**	**Length (nt)**	**Inconsistency (no. of intervals)**	**Inconsistency (cumulative nt)**	**Relative inconsistency**
Chr1c	59,282,136	1,272	4,994,109	8.4%	50,636,588	1,417	6,611,517	13.1%
Chr2c	74,718,367	1,611	11,203,540	15.0%	66,155,350	1,705	10,466,902	15.8%
Chr3c	41,337,678	1,211	4,909,582	11.9%	41,566,753	1,363	6,130,985	14.7%
Chr4c	51,611,264	1,058	6,960,485	13.5%	41,786,336	1,167	8,604,004	20.6%
Chr5c	47,630,375	923	4,470,519	9.4%	45,899,693	1,132	4,928,692	10.7%
Chr6c	62,962,097	1,205	8,301,775	13.2%	55,181,588	1,460	7,687,170	13.9%
Chr7c	39,944,450	1,040	4,460,667	11.2%	38,854,053	1,245	4,098,609	10.5%
Chr8c	44,319,397	1,092	5,703,932	12.9%	39,008,463	1,240	6,491,557	16.6%
Chr9c	43,933,066	724	3,521,073	8.0%	38,064,651	867	4,804,944	12.6%
Chr10c	51,900,066	1,071	6,678,105	12.9%	45,429,025	1,208	6,936,956	15.3%
Chr11c	39,995,608	1,320	10,102,819	25.3%	36,215,491	1,426	9,725,655	26.9%
Chr1e	52,005,836	980	24,831,183	47.7%	48,756,970	1,316	24,214,137	49.7%
Chr2e	73,705,077	1,784	21,084,309	28.6%	71,633,312	1,945	19,730,795	27.5%
Chr3e	44,118,139	1,781	12,047,158	27.3%	37,271,464	1,646	10,615,922	28.5%
Chr4e	47,362,295	1,001	10,914,623	23.0%	42,507,429	1,128	9,858,152	23.2%
Chr5e	48,524,476	1,303	9,732,646	20.1%	39,439,615	1,215	9,740,210	24.7%
Chr6e	59,109,944	1,887	21,931,455	37.1%	52,240,725	2,129	20,051,265	38.4%
Chr7e	39,730,949	1,027	8,507,142	21.4%	35,392,230	1,112	9,580,735	27.1%
Chr8e	49,651,447	1,912	7,739,977	15.6%	45,117,557	1,982	7,874,957	17.5%
Chr9e	39,158,766	1,271	9,938,712	25.4%	35,839,895	1,287	9,407,016	26.2%
Chr10e	43,085,031	1,468	12,078,135	28.0%	40,458,934	1,489	11,562,261	28.6%
Chr11e	44,702,780	1,703	15,712,703	35.1%	42,465,768	1,832	14,060,320	33.1%
Sum	1,098,789,244	28,644	225,824,649	20.6%	989,921,890	31,311	223,182,761	22.5%

Supplementary Table 5. Proportion of transposable elements in the whole genome and in A/B chromatin compartments. Breakdown by class and superfamily.

Superfamily	Percentage of haploid genome	Chromatin compartment					
		Cumulative length (bp)			Percentage of haploid genome length		
		A	B	n.d.	A	B	n.d.
Type I (Retrotransposon)							
LTR/Copia	5.15%	18,433,161	49,406,261	5,338	1.40%	3.75%	0.00%
LTR/Gypsy	24.11%	80,211,195	237,452,369	27,729	6.09%	18.02%	0.00%
LTR/unknown	21.34%	67,895,067	213,209,481	18,819	5.15%	16.18%	0.00%
nonLTR/LINE_element	0.16%	585,759	1,472,857	-	0.04%	0.11%	0.00%
nonLTR/unknown	0.00%	7,729	19,078	-	0.00%	0.00%	0.00%
nonTIR/helitron	2.14%	12,887,595	15,232,867	46,751	0.98%	1.16%	0.00%
Sum	*52.89%*	*180,020,506*	*516,792,913*	*98,637*	*13.66%*	*39.22%*	*0.01%*
Type II (DNA transposon)							
TIR/CACTA	0.92%	4,778,755	7,308,034	8,580	0.36%	0.55%	0.00%
TIR/hAT	1.19%	6,104,888	9,580,846	9,612	0.46%	0.73%	0.00%
TIR/Mutator	3.08%	17,196,685	23,405,822	35,659	1.31%	1.78%	0.00%
TIR/PIF_Harbinger	0.27%	1,403,872	2,203,625	2,811	0.11%	0.17%	0.00%
TIR/Tc1_Mariner	0.33%	2,165,935	2,215,537	6,736	0.16%	0.17%	0.00%
Sum	*5.80%*	*31,650,135*	*44,713,864*	*63,398*	*2.40%*	*3.39%*	*0.00%*
Grand Sum	*58.69%*	*211,670,641*	*561,506,777*	*162,035*	*16.06%*	*42.61%*	*0.01%*

n.d. = not determined. Within unanchored scaffolds and with genomic windows in chromosome pseudomolecules that could not be assigned to either compartment.

Supplementary Table 6. Shared and non-shared portions of the two subgenomes. Details by genomic window are provided graphically in Supplementary Figs. 19-24 and in the related Source Data file in tabular format.

Subgenome	Shared transposable elements	Homoeologous-specific transposable elements	Shared non-repetitive DNA	Other homoeologous-specific PAV	Shared sum	Subgenome length	Percent
Canephora	70,280,735	265,339,756	157,232,364	64,781,649	227,513,099	557,634,504	40.8%
Eugenioides	62,135,526	254,206,152	154,978,158	69,834,904	217,113,684	541,154,740	40.1%
Sum	132,416,261	519,545,908	312,210,522	134,616,553	444,626,783	1,098,789,244	

Supplementary Table 7. Features of the LTR-retrotransposons that are interspersed with the tandem repeat arrays on Chr7c generated by the 2,683-bp CRM-derived monomer (Supplementary Fig. 37). The estimated age of each insertional event was calculated based on intra-element LTR sequence divergence and a mutation rate of 1.3×10^{-8} per site per year used for annuals and divided by 3 years per generation (duration of the juvenile phase).

		TE			LTR1			LTR2			
**Chromosome**	**Repbase annotation**	**Start**	**End**	**Size**	**Start**	**End**	**Size**	**Start**	**End**	**Size**	**Estimated insertion time (MY)**
Chr7c	Gypsy-13	26,936,647	26,950,572	13,926	26,936,647	26,937,311	665	26,949,900	26,950,572	673	2.3076
Chr7c	Copia-35	27,039,305	27,043,187	3,883	27,039,305	27,040,207	903	27,042,299	27,043,187	889	3.4614
Chr7c	Gypsy-20	27,268,960	27,276,499	7,540	27,268,960	27,269,736	777	27,275,723	27,276,499	777	0.6000
Chr7c	Gypsy-13	27,276,845	27,285,618	8,774	27,276,845	27,277,519	675	27,284,946	27,285,618	673	2.7924
Chr7c	Gypsy-20	27,321,925	27,326,755	4,831	27,321,925	27,322,698	774	27,325,984	27,326,755	772	0.3000
Chr7c	Gypsy-20	27,509,998	27,517,508	7,511	27,509,998	27,510,738	741	27,516,768	27,517,508	741	0.9462
Chr7c	Gypsy-13	27,526,185	27,534,374	8,190	27,526,185	27,526,883	699	27,533,716	27,534,374	659	9.1962
Chr7c	Gypsy-13	27,534,547	27,539,343	4,797	27,534,547	27,535,147	601	27,538,743	27,539,343	601	9.1845

Supplementary Table 8. Gene expression levels of the homoeologous genes *Cara0010e09280* and *Cara0010c09420* spanning over the site of homoeologous recombination between Chr10c and Chr10e.

Cara0010e09280	**Chromosome**	**Start**	**End**	**Coverage**	**FPKM**	**TPM**
**Bud** Biorep A	Chr10e	7673621	7682949	20.673195	3.560728	6.626792
**Green Drupe** Biorep A	Chr10e	7673621	7682949	1.346083	0.235987	0.411509
**Shoot Apical Meristem** Biorep A	Chr10e	7673621	7682949	2.879896	1.142693	1.903697
**Bulked Drupes** Biorep A	Chr10e	7673621	7682949	0.270349	0.064085	0.116672
**Red Drupe** Biorep A Techrep 1	Chr10e	7673621	7682949	0.150293	0.032889	0.060317
**Red Drupe** Biorep A Techrep 2	Chr10e	7673621	7682949	1.180998	0.235636	0.439106
**Root** Biorep A	Chr10e	7673621	7682949	3.266066	0.954456	1.817727
**Root** Biorep B	Chr10e	7673621	7682949	5.207314	1.060804	2.054827
**Stem** Biorep A	Chr10e	7673621	7682949	4.574816	0.691267	1.234336
**Stem** Biorep B	Chr10e	7673621	7682949	9.103427	1.613985	2.996892
**Leaf** Biorep A	Chr10e	7673621	7682949	26.322521	4.896039	8.907329
**Leaf** Biorep B	Chr10e	7673621	7682949	22.859854	4.44055	8.193858

Cara0010c09420	**Chromosome**	**Start**	**End**	**Coverage**	**FPKM**	**TPM**
**Bud** Biorep A	Chr10c	7750335	7759523	20.206772	3.480392	6.477281
**Green Drupe** Biorep A	Chr10c	7750335	7759523	1.952358	0.342275	0.596853
**Shoot Apical Meristem** Biorep A	Chr10c	7750335	7759523	3.25008	1.289575	2.1484
**Bulked Drupes** Biorep A	Chr10c	7750335	7759523	0.146776	0.034793	0.063342
**Red Drupe** Biorep A Techrep 1	Chr10c	7750335	7759523	0.463426	0.101412	0.185986
**Red Drupe** Biorep A Techrep 2	Chr10c	7750335	7759523	1.275746	0.254541	0.474334
**Root** Biorep A	Chr10c	7750335	7759523	3.879532	1.133732	2.159151
**Root** Biorep B	Chr10c	7750335	7759523	6.315848	1.286628	2.492259
**Stem** Biorep A	Chr10c	7750335	7759523	5.866378	0.886425	1.582814
**Stem** Biorep B	Chr10c	7750335	7759523	8.684153	1.53965	2.858865
**Leaf** Biorep A	Chr10c	7750335	7759523	27.956692	5.199997	9.460319
**Leaf** Biorep B	Chr10c	7750335	7759523	21.224575	4.122895	7.607711

Supplementary Table 9. Haplotype sharing between introgressions lines and Catimors.

Introgression line	Shared genomic windows with Catimors carrying extra-Arabica SNPs	Extra-Arabica variant sites in the introgression line across introgressed windows shared with Catimors	Extra-Arabica variant sites shared with Catimors	Extra-Arabica variant sites not called in Catimors	Extra-Arabica variant sites not covered in Catimors	Introgressed haplotype matching
CHF1 (homozygous introgressions)	66 (P7963, T5175, T8667)	25400	25234	15	151	0.999405917
CHF1 (homozygous introgressions)	17 (T8667)	6913	6374	6	489	0.992216687
SL34 (homozygous introgressions)	144 (T8667)	44982	41834	67	2550	0.985906863
SL34 (heterozygous introgressions)	61 (T8667)	16695	12852	1196	1025	0.820165922

Supplementary Table 10. Resequencing data newly generated in the present study.

Sample identifier / Accession name	Metadata-based taxonomic assignment	Genomic composition-based assignment	Reported country of origin/sampling and holding repository	Reference for data and metadata	BioProject number	Coverage (X)
1-Geisha	*C. arabica*	*C. arabica*	Ethiopia, corresponding to the accession T.02722, introduced from CATIE to illycaffè SpA by seeds	This paper	PRJNA1001614	34
Kenya-SL28	*C. arabica*	*C. arabica*	Kenya, corresponding to the accession T.02739 introduced from CATIE to illycaffè SpA by seeds	This paper	PRJNA1001614	34
GEISHA	*C. arabica*	*C. arabica*	Ethiopia, World Coffee Research	This paper	PRJNA1001613	40
ET47	*C. arabica*	*C. arabica*	Ethiopia, World Coffee Research	This paper	PRJNA1001613	44

Supplementary Table 11. Primer sequences for the experimental validation of the homoeologous exchange on chromosomes 10.

Primer name	5'→3' primer sequence	Notes
Chr10-c_FW	GGAAATAGTAATTAATTTGTACTGC	
Chr10-c_RV	TCTTTAAGTCTCATCTAAGACATA	The 5'-end (5-bp) of Chr10-c_RV does not align to Chr10e, but the 3'-end (18-bp) aligns without mismaches to Chr10e not providing specificity for the amplification of the wild-type Chr10c
Chr10-idreg_FW	TCTTTTAACTTCTCTGCTTG	
Chr10-idreg_RV	AAGAATCACATGTCTGAAAG	
Chr10-e_FW	GTAATTAATTTGTACAACAGC	
Chr10-e_RV	CTTTTGTTTTTCTTGGTTACA	
Chr10-idreg_FW	TCTTTTAACTTCTCTGCTTG	
Chr10-idreg_RV	AAGAATCACATGTCTGAAAG	

Supplementary references

1. Ghurye, J., Pop, M., Koren, S., Bickhart, D. & Chin, C. S. Scaffolding of long read assemblies using long range contact information. *BMC Genomics* **18**, 527 (2017).
2. Kurtz, S. *et al.* Versatile and open software for comparing large genomes. *Genome Biol.* **5**, R12 (2004).
3. Durand, N. C. *et al.* Juicer provides a one-click system for analyzing loop-resolution Hi-C experiments. *Cell Syst.* **3**, 95–98 (2016).
4. Sedlazeck, F. J. *et al.* Accurate detection of complex structural variations using single-molecule sequencing. *Nat. Methods 2018 156* **15**, 461–468 (2018).
5. Robinson, J. T. *et al.* Integrative Genomics Viewer. *Nat. Biotechnol.* **29**, 24 (2011).
6. Ou, S. *et al.* Benchmarking transposable element annotation methods for creation of a streamlined, comprehensive pipeline. *Genome Biol.* **20**, 1–18 (2019).
7. Rice, P., Longden, L. & Bleasby, A. EMBOSS: The European Molecular Biology Open Software Suite. *Trends Genet.* **16**, 276–277 (2000).
8. Ma, J. & Bennetzen, J. L. Rapid recent growth and divergence of rice nuclear genomes. *Proc. Natl. Acad. Sci. U. S. A.* **101**, 12404–10 (2004).
9. Dvorkina, T., Bzikadze, A. V. & Pevzner, P. A. The string decomposition problem and its applications to centromere analysis and assembly. *Bioinformatics* **36**, 93–101 (2020).
10. Katoh, K. & Standley, D. M. MAFFT Multiple Sequence Alignment Software Version 7: Improvements in performance and usability. *Mol. Biol. Evol.* **30**, 772–780 (2013).
11. Minh, B. Q. *et al.* IQ-TREE 2: New models and efficient methods for phylogenetic inference in the genomic era. *Mol. Biol. Evol.* **37**, 1530–1534 (2020).
12. Letunic, I. & Bork, P. Interactive Tree Of Life (iTOL) v5: an online tool for phylogenetic tree display and annotation. *Nucleic Acids Res.* **49**, 293–296 (2021).
13. Scalabrin, S. *et al.* A single polyploidization event at the origin of the tetraploid genome of *Coffea arabica* is responsible for the extremely low genetic variation in wild and cultivated germplasm. *Sci. Reports 2020 101* **10**, 1–13 (2020).
14. Fernie, L., Greathead, D., Meyer, F., Monaco, L. & Narasimhaswamy, R. *FAO coffee mission to Ethiopia, 1964-65. FAO* (1968).
15. Guillaumet, J. L. & Halle, F. *Etude de la variabilité du* Coffea arabica *dans son aire d'origine. Rapport sur la mission ORSTOM dans le Sud Ouest de l'Ethiopie: 12 novembre - 18 décembre 1966*. (1967).
16. Guillaumet, J. & Halle, F. Échantillonnage du matériel *Coffea arabica* récolté en Éthiopie. in *Etude de la structure et de la variabilite genetique des cafeiers* 13–18 (1978).
17. Scalabrin, S. *et al.* A chromosome-scale assembly reveals chromosomal aberrations and exchanges generating genetic diversity in *Coffea arabica* germplasm. Data sets. *figshare*

10.6084/m9.figshare.23821881 (2023).

18. Marroni, F. *et al.* Reduction of heterozygosity (ROH) as a method to detect mosaic structural variation. *Plant Biotechnology Journal* **15**, 791–793 (2017).

19. Mekbib, Y. *et al.* Whole-genome resequencing of *Coffea arabica* L. (Rubiaceae) genotypes identify SNP and unravels distinct groups showing a strong geographical pattern. *BMC Plant Biol.* **22**, 69 (2022).

20. Huang, L. *et al.* Resequencing 93 accessions of coffee unveils independent and parallel selection during *Coffea* species divergence. *Plant Mol. Biol.* **103**, 51–61 (2020).

21. Meyer, F. Notes on wild *Coffea arabica* from Southwestern Ethiopia, with some historical considerations. *Econ. Bot.* **19**, 136–151 (1965).

22. Beck, H. E. *et al.* Present and future Köppen-Geiger climate classification maps at 1-km resolution. *Sci. Data* **5**, (2018).

23. Beck, H. E. *et al.* Present and future Köppen-Geiger climate classification maps at 1-km resolution. figshare. Dataset. https://doi.org/10.6084/m9.figshare.6396959 (2018).
