# Supplementary material for: A chromosome-scale assembly reveals chromosomal aberrations and exchanges generating genetic diversity in Coffea arabica germplasm
Source: Nat Commun. 2024 Jan 23;15:463. doi: 10.1038/s41467-023-44449-8 (PMC10805892; doi:10.1038/s41467-023-44449-8)
Supplement: Supplementary file 3 — Description of Additional Supplementary Files [file 41467_2023_44449_MOESM3_ESM.pdf]

Description of Additional Supplementary Files

File Name: Supplementary Data 1
Description: Resequencing data obtained from public repositories.

File Name: Supplementary Data 2
Description: Gene ontology enrichment analysis in the chromosome segments of C. arabica that tend to be replaced by Timor hybrid-derived haplotypes in Timor hybrid derivatives.

File Name: Supplementary Data 3
Description: GBS data obtained from public repositories (BioProject number PRJNA554647).

File Name: Supplementary Data 4
Description: *C. arabica* accessions showing large (>1 Mb) deletions or duplications.

File Name: Supplementary Data 5
Description: *C. arabica* accessions showing homoeologous exchanges.

File Name: Supplementary Data 6
Description: Characterisation of the exchanged homoeologous chromosomal segments, from the homoeologous exchange (HE) site to the nearest telomeric end, and of the region spanning HE sites (1-Mbp interval around the approximate location of each HE event).

File Name: Supplementary Data 7
Description: Gene ontology enrichment analysis in exchanged homoeologous chromosomal segments (from the site of homoeologous exchange to the nearest telomeric end).
