# Supplementary material for: A chromosome-scale assembly reveals chromosomal aberrations and exchanges generating genetic diversity in Coffea arabica germplasm
Source: Nat Commun. 2024 Jan 23;15:463. doi: 10.1038/s41467-023-44449-8 (PMC10805892; doi:10.1038/s41467-023-44449-8)
Supplement: Supplementary file 11 — Reporting Summary [file 41467_2023_44449_MOESM11_ESM.pdf]

Corresponding author(s): Gabriele Di Gaspero, Michele Morgante

Last updated by author(s): Dec 6, 2023

Reporting Summary

Nature Portfolio wishes to improve the reproducibility of the work that we publish. This form provides structure for consistency and transparency in reporting. For further information on Nature Portfolio policies, see our Editorial Policies and the Editorial Policy Checklist.

Statistics

For all statistical analyses, confirm that the following items are present in the figure legend, table legend, main text, or Methods section.

n/a	Confirmed	
☐	☒	The exact sample size (*n*) for each experimental group/condition, given as a discrete number and unit of measurement
☒	☐	A statement on whether measurements were taken from distinct samples or whether the same sample was measured repeatedly
☐	☒	The statistical test(s) used AND whether they are one- or two-sided<br>*Only common tests should be described solely by name; describe more complex techniques in the Methods section.*
☒	☐	A description of all covariates tested
☒	☐	A description of any assumptions or corrections, such as tests of normality and adjustment for multiple comparisons
☐	☒	A full description of the statistical parameters including central tendency (e.g. means) or other basic estimates (e.g. regression coefficient) AND variation (e.g. standard deviation) or associated estimates of uncertainty (e.g. confidence intervals)
☐	☒	For null hypothesis testing, the test statistic (e.g. *F*, *t*, *r*) with confidence intervals, effect sizes, degrees of freedom and *P* value noted<br>*Give P values as exact values whenever suitable.*
☒	☐	For Bayesian analysis, information on the choice of priors and Markov chain Monte Carlo settings
☒	☐	For hierarchical and complex designs, identification of the appropriate level for tests and full reporting of outcomes
☒	☐	Estimates of effect sizes (e.g. Cohen's *d*, Pearson's *r*), indicating how they were calculated

Our web collection on statistics for biologists contains articles on many of the points above.

Software and code

Policy information about availability of computer code

Data collection	No software was used

Data analysis

ONT reads were corrected and assembled with Canu v2.0 (ErrorRate=0.4, correctedErrorRate=0.144, minReadLength=10000, minOverlapLength=3000, ovlMerDistinct=0.975, corMhapSensitivity=high, saveReads=True, "batOptions=-dg 3 -db 3 -dr 1 -ca 500 -cp 50). In order to polish the contig, Illumina reads were aligned using BWA-MEM v0.7.17 and base correction was performed using Pilon v1.23. Hi-C reads were aligned to the reference genome using BWA-MEM v0.7.17 (-5SP) and filtered using samblaster v0.1.26. Scaffolding was performed using SALSA v2.3. Principal component values on aligned Hi–C reads were produced using the HOMER utility runHiCpca.pl. BAC clone sequences were assembled using ABySS v1.3.7. Ab initio gene prediction was performed using SNAP, Glimmer v3.02b, BRAKER2 v2.1.5 and Geneid v1.4.4. Final gene models were generated using EvidenceModeler v1.1.1 and PASA2. Gene annotation was performed with Pannzer2. Gene Ontology assignment was performed using goslim_plant database (update 27/07/2023) and enrichment analysis was performed using the R package topGO v2.52.0. Transposable elements were identified using EDTA v1.9.6 and RepeatMasker v4.0.6. RNA reads were aligned using STAR v2.7.10a. DNA reads were aligned using BWA-MEM v0.7.17. Single Nucleotide Polymorphisms were called using UnifiedGenotyper tool in GATK v3.3.0. Chromosome–scale visualization of tandem repeat structures with identity heatmaps were generated with StainedGlass v0.4. Synteny plots were obtained with SyRI v1.6. Two-sided Wilcoxon test was applied to statistically test the box-plot distributions. Deviations from the variant frequency that is expected under the normal condition of 2:2 homoeologous copy number, which we named here Reduction Of Homoeologous Heterozygosity (ROHH) were identified using the software χ-scan with the chi_reads algorythm. Depth of Coverage (DOC) was calculated using the command genomecov in bedtools. Phylogenetic trees were constructed using vcf-kit. Graphs were plotted using R v3.6.1 using the data provided in the Source Data file. Variant sites were called from GBS reads using Stacks v.2.1. PCA was computed using the R package SNPRelate v1.8.0. Monomeric sequences of tandem repeated arrays were extracted with StringDecomposer v1.1.2. Multiple alignments of monomeric sequences were generated using MAFFT v7.475. The phylogenetic tree of monomeric sequences swas constructed using IQ-TREE v2.1.2 and plotted with iTOL.

For manuscripts utilizing custom algorithms or software that are central to the research but not yet described in published literature, software must be made available to editors and reviewers. We strongly encourage code deposition in a community repository (e.g. GitHub). See the Nature Portfolio guidelines for submitting code & software for further information.

Data

Policy information about availability of data

All manuscripts must include a data availability statement. This statement should provide the following information, where applicable:

- Accession codes, unique identifiers, or web links for publicly available datasets
- A description of any restrictions on data availability
- For clinical datasets or third party data, please ensure that the statement adheres to our policy

The data generated in this study have been deposited in the NCBI database under the following BioProject numbers: raw sequences and the genome assembly of 'Bourbon' PRJNA944143 [https://www.ncbi.nlm.nih.gov/bioproject/?term=PRJNA944143], raw sequences of 4 accessions (Supplementary Table 10) PRJNA1001613 [https://www.ncbi.nlm.nih.gov/bioproject/?term=PRJNA1001613] and PRJNA1001614 [https://www.ncbi.nlm.nih.gov/bioproject/?term=PRJNA1001614]. Source data are provided with this paper. The following genome features of the Bourbon assembly are graphically available using the genome browser at https://coffea.appliedgenomics.org/ and accessible upon registration: gene predictions supported by evidence of RNA read alignments, repeat annotation, k-mers, synteny and collinearity between homoeologs. Raw sequence data for genetic diversity analysis were obtained from the BioProject numbers PRJNA505204 [https://www.ncbi.nlm.nih.gov/bioproject/?term=PRJNA505204], PRJNA790687 [https://www.ncbi.nlm.nih.gov/bioproject/?term=PRJNA790687], PRJNA554647 [https://www.ncbi.nlm.nih.gov/bioproject/?term=PRJNA554647], PRJNA497891 [https://www.ncbi.nlm.nih.gov/bioproject/?term=PRJNA497891]. Large data sets are deposited in the figshare repository43 with the following DOI 10.6084/m9.figshare.23821881 [https://figshare.com/articles/figure/_b_A_chromosome-scale_assembly_reveals_chromosomal_aberrations_and_exchanges_generating_genetic_diversity_in_b_b_i_Coffea_arabica_i_b_b_germplasm_b_/23821881]. They include: Reduction Of Homoeologous Heterozygosity (ROHH) and Depth of Coverage (DOC) analysis in C. arabica with data organized according to individual genotype as well as to individual chromosome, including simulations of read coverage variation; introgression analysis in the GBS diversity panel; genome annotation data (gene prediction and repeat annotation in GFF3 File Format; gene annotation in txt format).

Research involving human participants, their data, or biological material

Policy information about studies with human participants or human data. See also policy information about sex, gender (identity/presentation), and sexual orientation and race, ethnicity and racism.

Reporting on sex and gender	N/A
Reporting on race, ethnicity, or other socially relevant groupings	N/A
Population characteristics	N/A
Recruitment	N/A
Ethics oversight	N/A

Note that full information on the approval of the study protocol must also be provided in the manuscript.

Field-specific reporting

Please select the one below that is the best fit for your research. If you are not sure, read the appropriate sections before making your selection.

☒ Life sciences ☐ Behavioural & social sciences ☐ Ecological, evolutionary & environmental sciences

Life sciences study design

All studies must disclose on these points even when the disclosure is negative.

Sample size	Raw sequence data for genetic diversity analysis were retrieved from public repositories. Data were obtained from the following BioProjects: PRJNA505204,PRJNA790687, PRJNA554647, PRJNA497891. Genetic diversity analysis was conducted using a sample of 174 Coffea sp accessions. Selection bias was excluded by the use of the entire set of publicly available sequencing data downloaded from public databases as of December 2022 from the two largest resequencing projects in Coffea arabica: Huang et al 2020 and Mekbib et al 2022.
Data exclusions	One accession of C. eugenioides was excluded from the graph in Fig.3a, as reported in the Figure Legend. The exclusion criteria for Fig.3a were pre-established because Fig. 3a is intended to show the relations among present-day diploid C. canephora accessions, pure C. arabica acccessions, and C. arabica accessions that contain recent C. canephora neo-introgression. The complete graph including the accession of C. eugenioides is provided in Supplementary Fig. 43, which conveys along PC1 exactly the same evidence as above. Introducing C. eugenioides in Supplementary Fig. 43 masked the second principal component of variation among present-day C. canephora, C. arabica, and C. arabica containing C. canephora neo-introgression because PC2 in Supplementary Fig. 43 accounts for variation across the eugenioides subgenome, which is quasi-null among accessions of C. arabica in comparison with between accessions of C. arabica and diploid C. eugenioides. We therefore placed Fig.3a in the main text and Supplementary Fig. 43 in the Supplementary Information, and not vice versa, because Fig.3a graphically conveys more information than Supplementary Fig. 43.<br><br>In the PCA analysis only, the C. canephora accession 33-1 has been removed due to its low number of raw reads and low coverage after read alignment (2X, Supplementary Data 1). The exclusion criteria for this accession were not pre-established. We used as an input for SNP calling all available accessions from the public datasets of Huang et al 2020 and Mekbib et al 2022, in order to comply with the requirement of not introducing a selection bias, regardless of any technical issue that could affect any sample. Then, in specific downstream analyses, such as the PCA, where technical issues affecting individual samples, such as low coverage, impacted the integrity of the output we had to remove technically critical samples.<br><br>All together, the treatment of the accession of C. eugenioides as an outgroup with respect to C. canephora-derived variation in C. arabica and the exclusion of the low coverage accession C. canephora accession '33-1' in PCA plots account for the fact that Fig. panels and Supplementary Figs. contain when specified in their Legends either 172 or 173 samples with respect to the set of 174 samples that is listed in the table of Supplementary Data 1 and that is used elsewhere in the article, if not otherwise indicated.
Replication	Hi-C data were generated using a single leaf sample and were not replicated. DNA sequencing data were not replicated in terms of biological replicates because there is no environmental factor affecting their result. Technical replication is provided by the read coverage. Two replicates were produced for each RNAseq sample. All attempts at replication were successful.
Randomization	Not relevant to this study. No a priori group allocation has been used. For genetic diversity analyses, taxonomic assignment based on Metadata was not used for a priori grouping.
Blinding	Not relevant to this study. No a priori group allocation has been used. For the genetic diversity experiment, investigators were not blind to group allocation during data collection because blinding was not relevant to data collection. We used all publicly available sequencing data of accessions held in germplasm collections. This study did not address natural population variation. For the same genetic diversity experiment, investigators were blind to group allocation because the taxonomic assignment of each accession, whose raw reads were drawn from public BioProjects, as reported in their associated Metadata was not used for a priori grouping.

Reporting for specific materials, systems and methods

We require information from authors about some types of materials, experimental systems and methods used in many studies. Here, indicate whether each material, system or method listed is relevant to your study. If you are not sure if a list item applies to your research, read the appropriate section before selecting a response.

Materials & experimental systems

n/a	Involved in the study
☒	☐ Antibodies
☒	☐ Eukaryotic cell lines
☒	☐ Palaeontology and archaeology
☒	☐ Animals and other organisms
☒	☐ Clinical data
☒	☐ Dual use research of concern
☒	☐ Plants

Methods

n/a	Involved in the study
☒	☐ ChIP-seq
☒	☐ Flow cytometry
☒	☐ MRI-based neuroimaging

Plants

Seed stocks	The specimens sequenced in this study were collected from seedlings raised from seeds introduced from the CATIE germplasm repository (entry numbers T.02722 and T.02739) and from stocks maintained at World Coffee Research (Geisha and ET47) as reported in Supplementary Table 10. Plant specimens were not collected from the field. The sequenced specimen of 'Bourbon' derived from the propagation of plant material originally used by Scalabrin et al (2020) A single polyploidization event at the origin of the tetraploid genome of Coffea arabica is responsible for the extremely low genetic variation in wild and cultivated germplasm. Scientific Reports 10: 4642
Novel plant genotypes	No novel plant genotype was produced.
Authentication	True-to-type-ness of seedlings and stocks from which sequenced specimens were taken was assessed by visual assessment of distinctive varietal traits by coffee agronomists at illycaffè SpA and at World Coffee Research. In addition to this, 'Geisha' samples were treated according to the work of authentication performed at World Coffee Research by Pruvot-Woehl S, Krishnan S, Solano W, Schilling T, Toniutti L, Bertrand B, Montagnon C (2020) Authentication of Coffea arabica varieties through DNA fingerprinting and its significance for the coffee sector. Journal of AOAC INTERNATIONAL 103: 325–334. WGS profiles that we obtained from the newly sequenced specimens were finally compared with GBS profiles of the specimens originating from the CATIE repository.
